# Supplementary material for: Visceral fat lipolysis by pancreatic lipases worsens heart failure
Source: Cell Rep Med. 2025 Jun 2;6(6):102147. doi: 10.1016/j.xcrm.2025.102147 (PMC12208342; doi:10.1016/j.xcrm.2025.102147)
Supplement: Document S1. Figures S1–S17 [file mmc1.pdf]

Cell Reports Medicine, Volume *6*

## Supplemental information

# Visceral fat lipolysis by

# pancreatic lipases worsens heart failure

**Nabil Smichi, Biswajit Khatua, Sergiy Kostenko, Cristiane de Oliveira, Bara El Kurdi, Kalpit Himmatbhai Devani, Shubham Trivedi, Megan Summers, Bryce McFayden, Sarah Navina, Krutika Patel, Sarah Jahangir, Marek Belohlavek, and Vijay P. Singh**

**Supplementary figures**

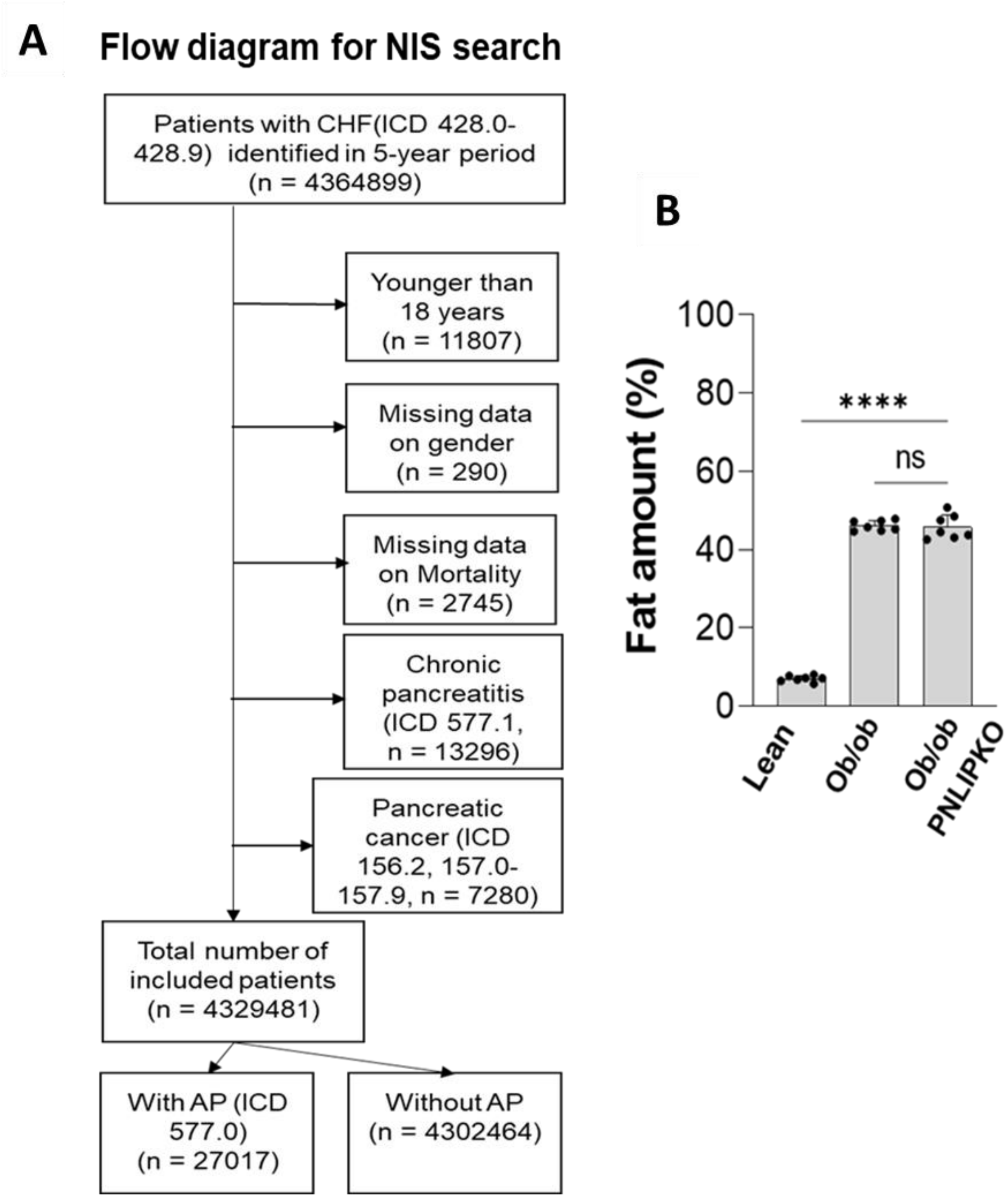


**Figure S1: (A)** The flow diagram for NIS search. **(B)** NMR data comparing the fat percentage in lean and ob/ob mice. Related to Figure 1.

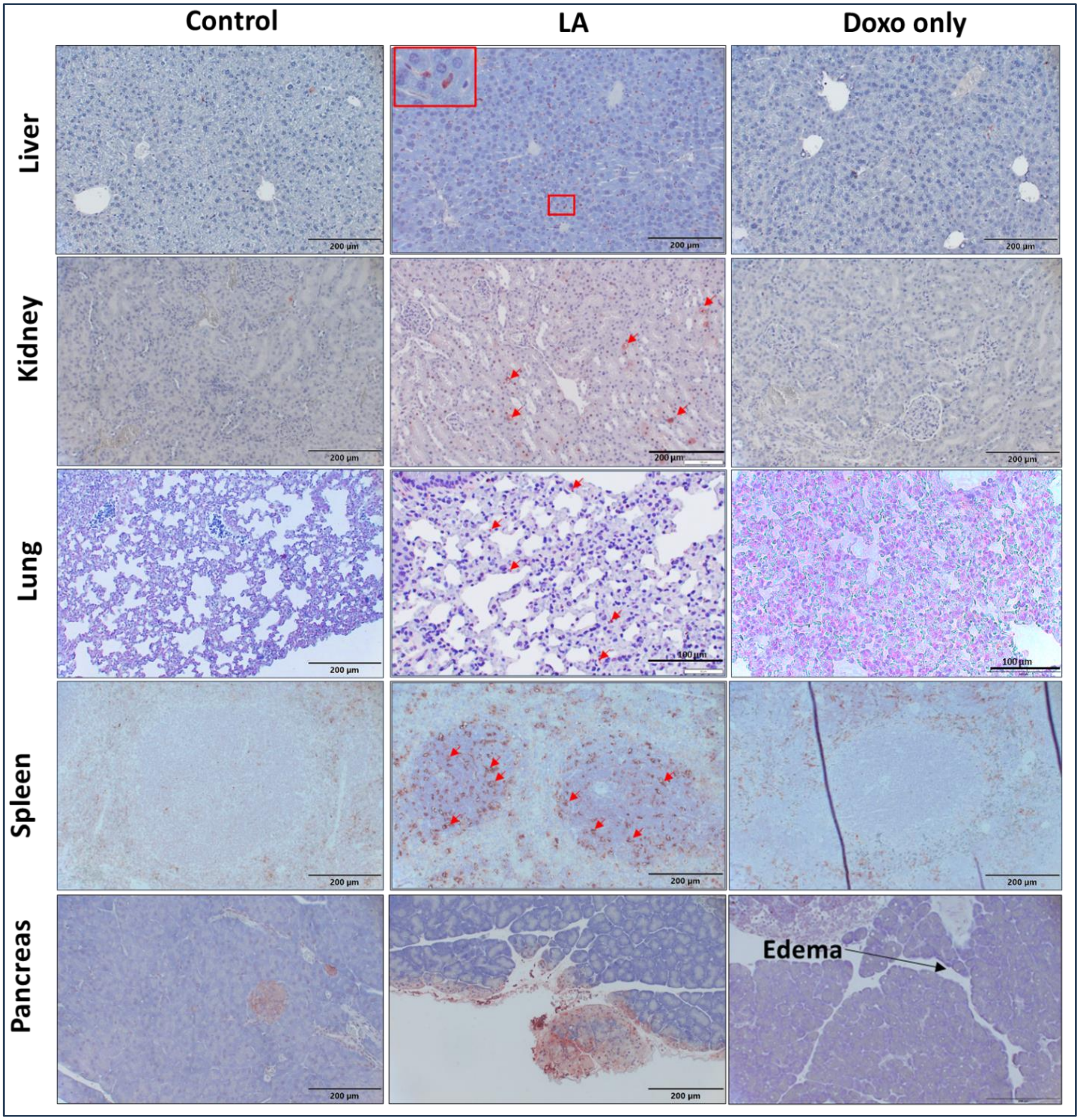


**Figure S2: TUNEL stained images of the liver, kidney, lungs, spleen, and pancreas from different groups of mice.** Related to Figure 1. The tissues represented in the image are mentioned on the left side, and the treatment group is above each column of images. The brown staining, emphasized with red arrows, shows the TUNEL positivity. Magnification was 20x.

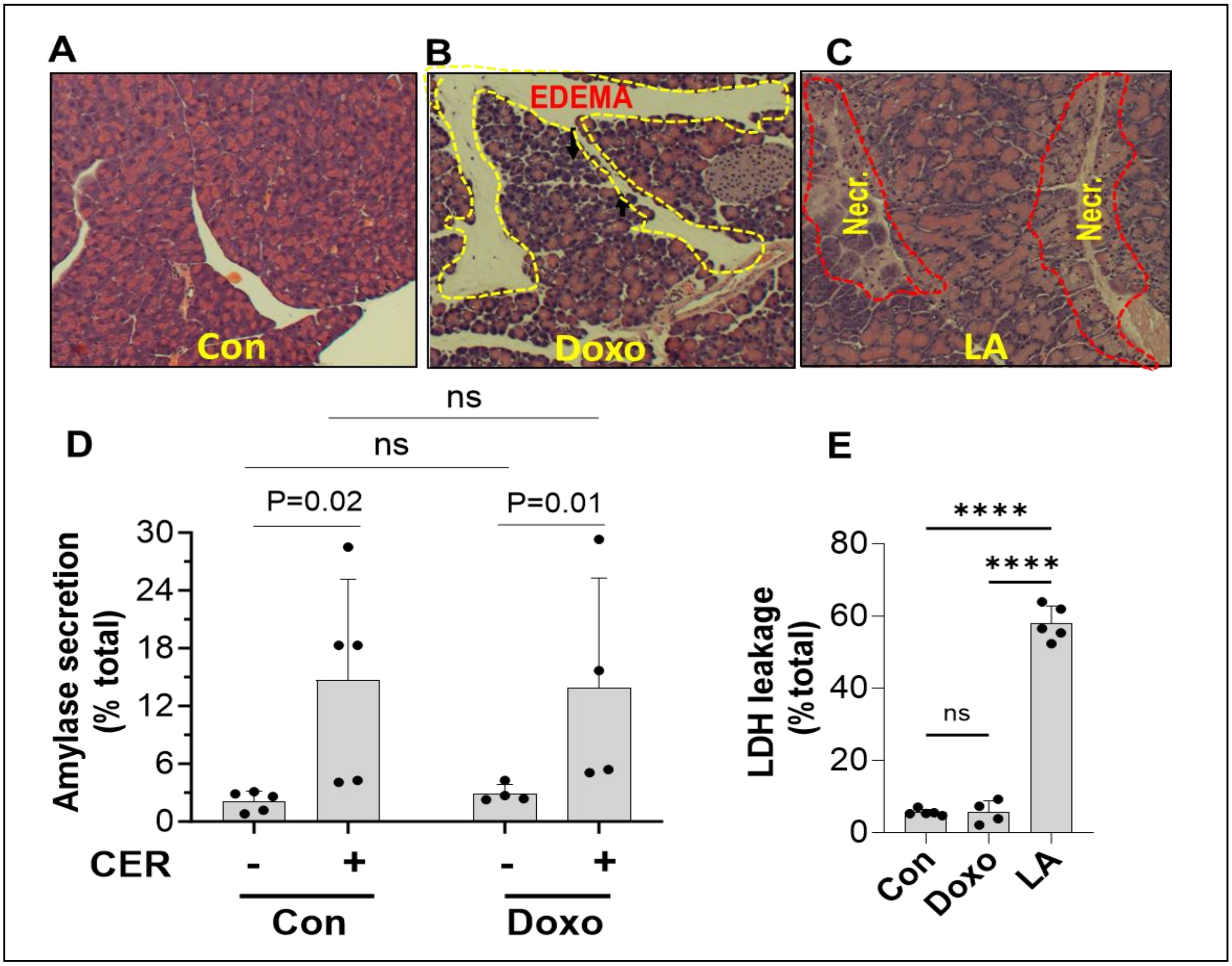


**Figure S3: Effect of doxorubicin on pancreatic injury *in-vivo* and acinar function and injury *ex-vivo*.** Related to Figure 1. Pancreas H&E staining images (10X) of untreated (**A**), Doxo (15mg/kg BW, B), and LA (0.2% BW, C) treated mice. LA-induced acinar necrosis [Necr.; Red dashed outline in (**C**)]but Doxo causes interstitial edema [yellow dashed outline; (**B**)] consistent with heart failure. (**D**) Physiological ($10^{-10}$M) caerulein (CER)-induced amylase secretion measured as % total is similar in Doxo ($1mg.mL^{-1}$) treated and untreated acini. (**E)** Doxo ($1mg.mL^{-1}$) treated acini showed similar LDH leakage compared to untreated acini over 4hrs, while LA (100mM) induced 58±5%leakage.**p<0.01, ***p<0.001; ****p<0.0001 indicate a significant difference between these groups compared to the control as determined by 1-way ANOVA. Data are represented as mean/± SD.

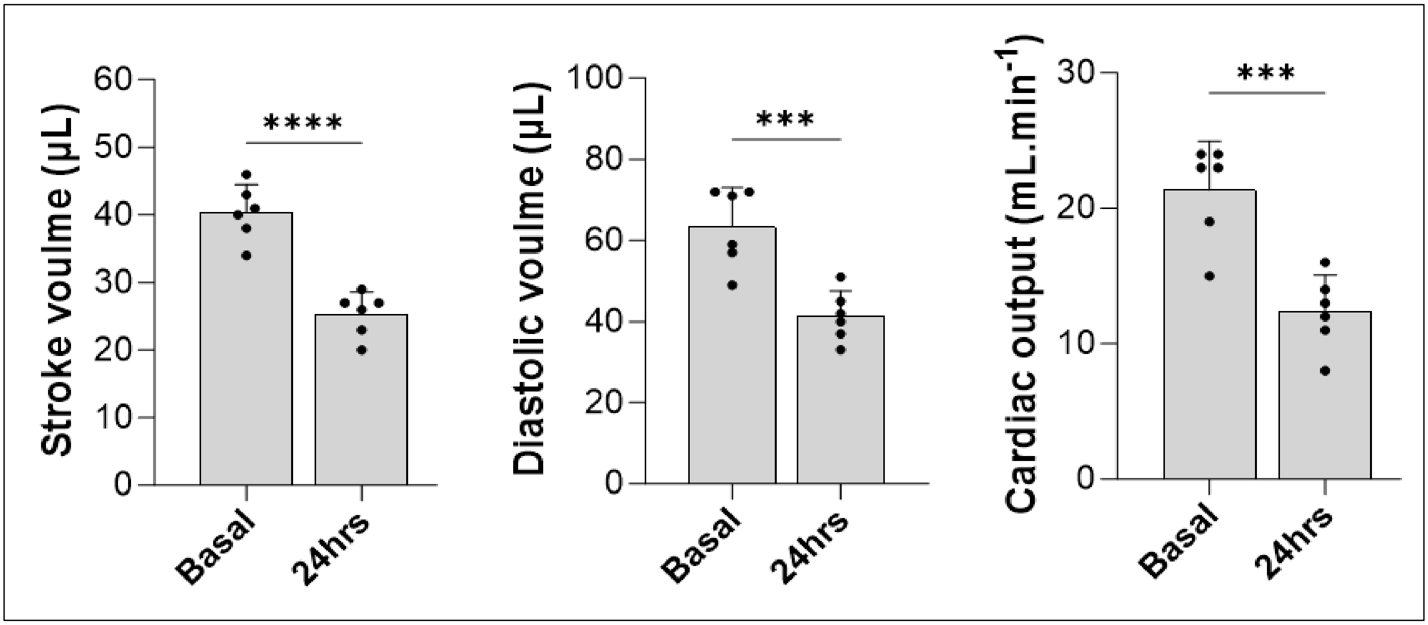


**Figure S4: Echocardiographic parameters in mice with Doxorubicin (Doxo) induced heart failure measured at baseline and 24 hours after Doxo.** Related to Figure 1. From left to right, left ventricular stroke volume (µL), end-diastolic volume (µL), and cardiac output ($mL.min^{-1}$). The ***p<0.001 and ****p<0.0001 indicate a significant reduction at 24 hours compared to the baseline. Data are represented as mean/± SD. The parameters were collected in the short-axis mode.

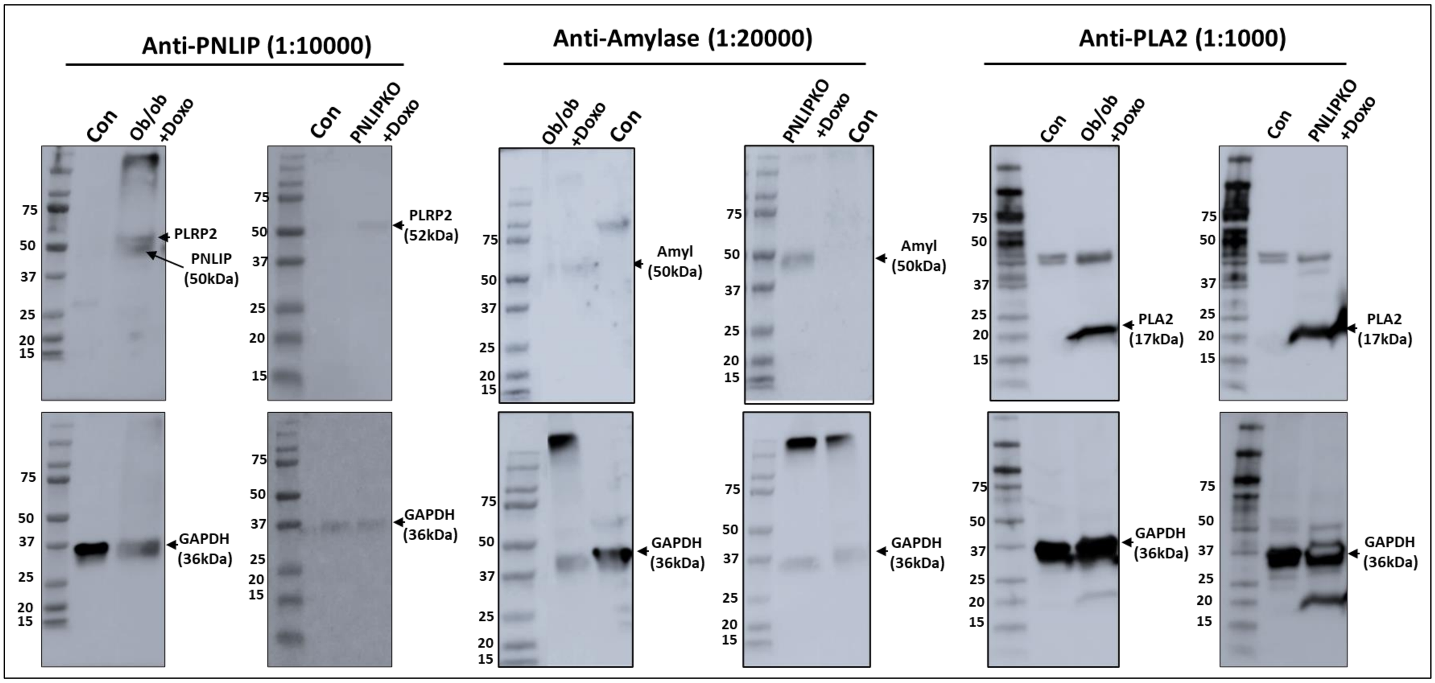


**Figure S5:** Western blot full gel images showing the leakage of PNLIP (50kDa), PNLIPRP2 (52kDa), $PLA_2$ (17kDa), and amylase (50kDa) from the pancreas into the fat pad of ob/ob and ob/ob PNLIP KO mice treated by doxorubicin (Doxo). The GAPDH (36kDa) was used as a control. Related to Figure 1.

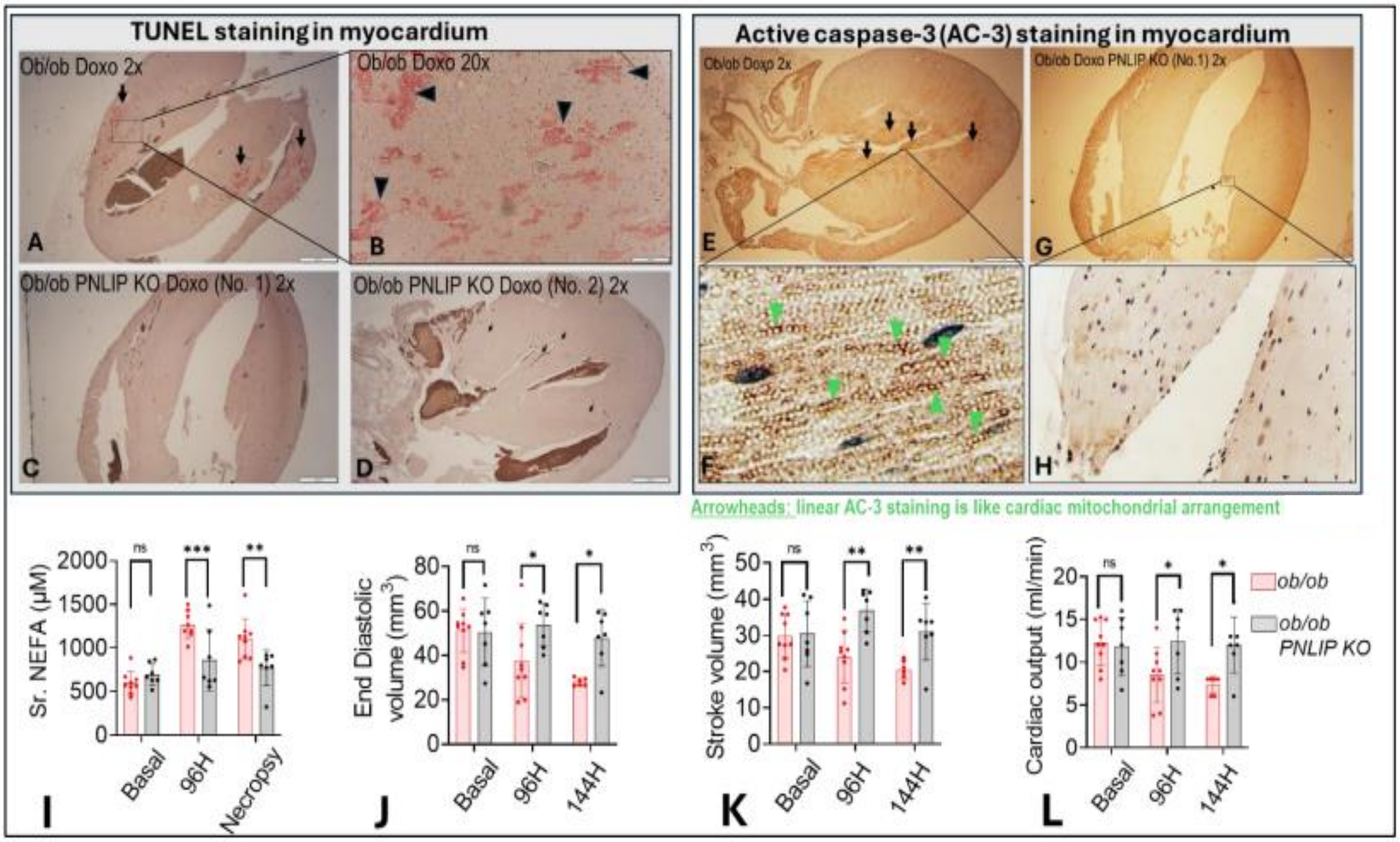


**Figure S6: Effects of genetic deletion of PNLIP (PNLIP KO) on doxorubicin (Doxo) induced Cardiac injury parameters and serum NEFA in genetically obese ob/ob mice.** Related to Figure 1. (**A-H)** Histologic images of the heart stained for TUNEL (**A-D**) or active caspase-3 (**E-H**). The treatment, genetic background and magnification are mentioned on the top. **B, F**, and **H** are zooms of the corresponding insets shown. Black arrowheads in **B** point to strong TUNEL staining in the heart of the ob/ob mouse treated with doxorubicin. Green arrows in **F** point to magnified views showing a strong linear round pattern of active caspase-3 (AC-3) staining in the heart of an ob/ob mouse treated with doxorubicin. This strong linear pattern resembles mitochondrial arrangement in mouse heart muscle, shown in the transmission electron microscopy (TEM) image below. These sections are being processed for TEM. (**I-L)** Bar graphs show a comparison of parameters for ob/ob mice (red) vs. ob/ob PNLIP KO mice (gray) at baseline (basal), day 4 (96 hrs), day 6 (144hrs) or necropsy. These are NEFA (**I**) and Echocardiography parameters: end-diastolic volume (**J**), stroke volume (**K**), and cardiac output (**L**) measured in the long-axis mode.*p<0.05, **p<0.01, ***p<0.001; indicate a significant difference between these groups compared to the control as determined by 1-way ANOVA. Data are represented as mean/± SD.

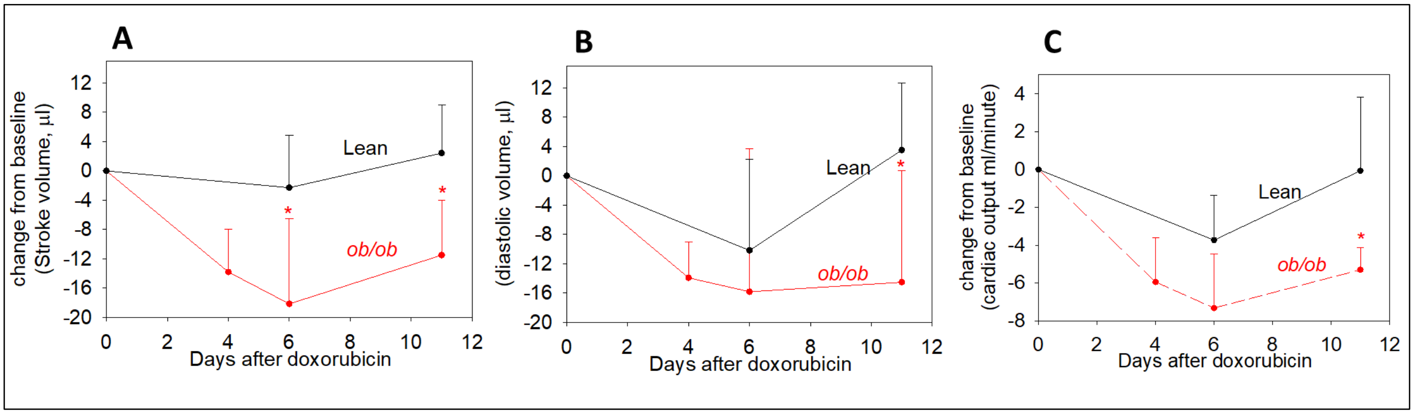


**Figure S7:** Effect of low-dose doxorubicin (2.5mg/kg/day IP) model on change in cardiac parameters (mentioned on Y-axis) in lean (C57bl/6, black color) and obese mice (ob/ob, red color). Related to Figure 1. Data are represented as mean/± SD.

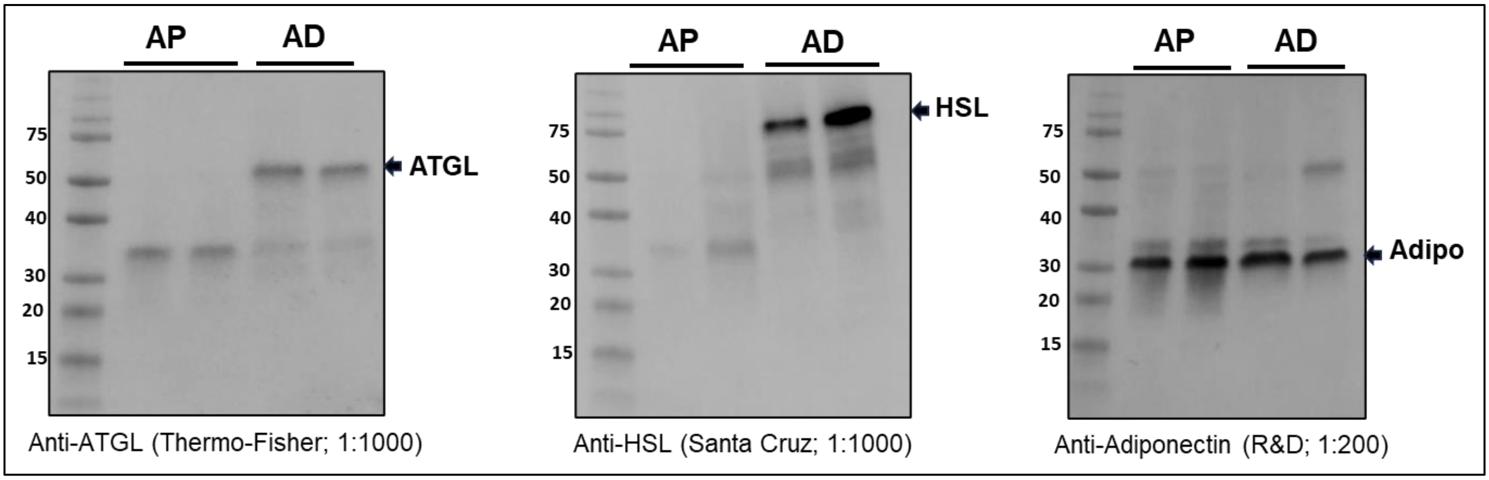


**Figure S8:** Western blot full gel images comparing detectable ATGL, HSL, and adiponectin (Adipo) bands in fat from human pancreatitis (AP) and diverticulitis (AD) samples. Related to Figure 2.

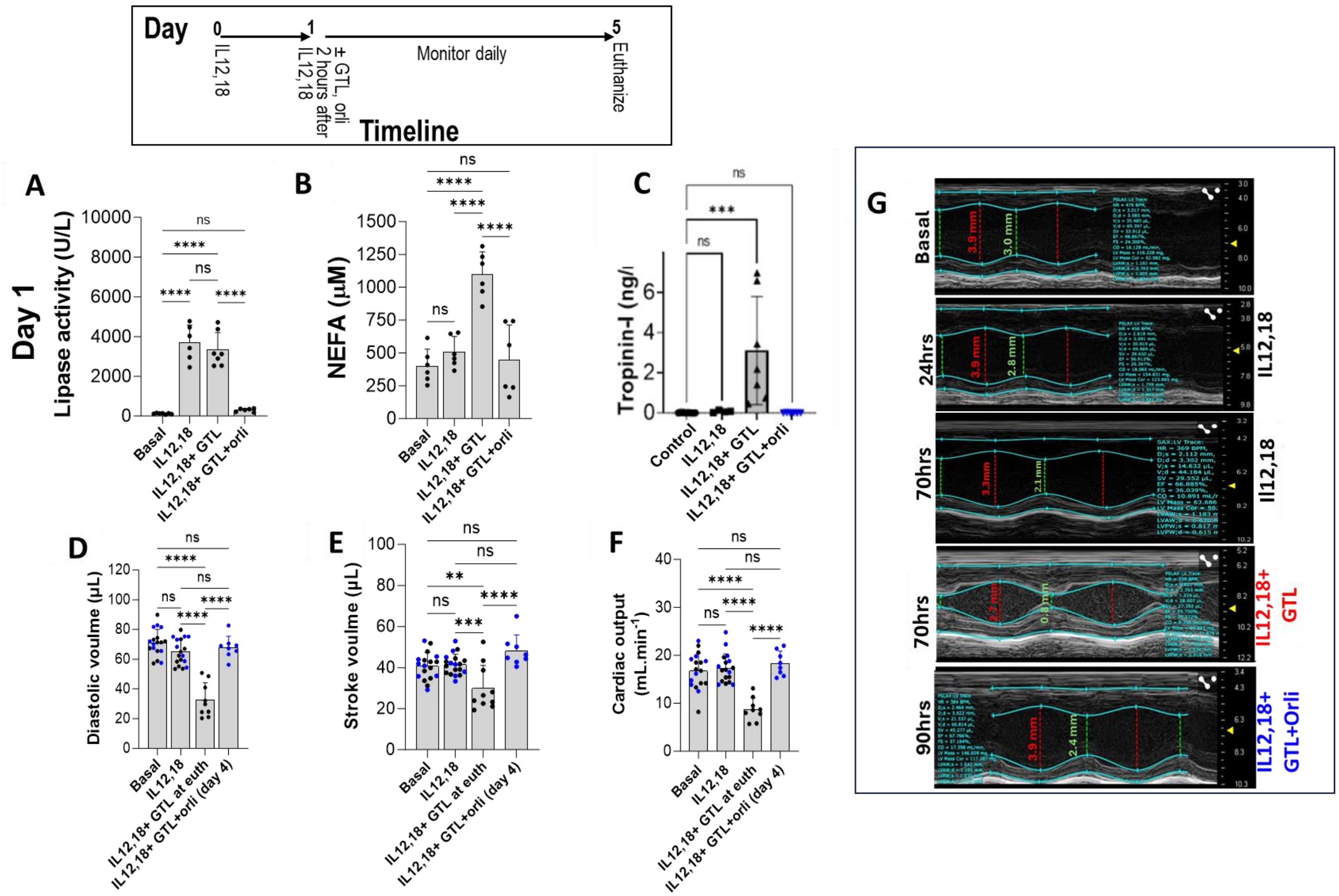


**Figure S9**: **Timeline and parameters of IL12,18 pancreatitis in lean mice.** Related to Figure 3. (**A**) Serum lipase and NEFA (**B**) in control mice (basal) compared to IL12,18, IL12,18 +GTL, and IL12,18+orlistat (Orli) ones, and IL12,18 AP. (**C**) The troponin-I levels in the tail vein prior to euthanasia of these mice. **D-F:** Show echocardiography parameters at baseline or prior to euthanasia. These are end-diastolic volume (**D**), stroke volume (**E**), and cardiac output (**F**) measured in the long-axis mode. (**G)** shows representative M-mode Echocardiographic images at baseline (top panels), and after inducing IL12, 18 pancreatitis (other panels) at time points mentioned on the left side of the corresponding treatment panel. **p<0.01, ***p<0.001; ****p<0.0001 indicate a significant difference between these groups compared to the control as determined by 1-way ANOVA. Data are represented as mean/± SD.

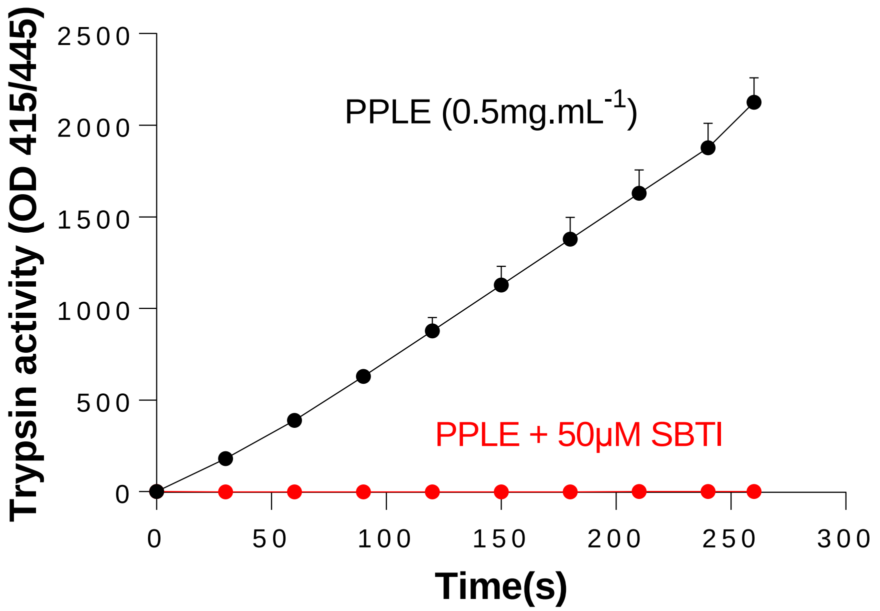


**Figure S10: Trypsin activity in the PPLE Extract and its inhibition by soybean trypsin inhibitor prior to injection into fat pads**. Related to Figure 3. The trypsin activity of 0.5 mg. $mL^{-1}$ was measured at 37°C in the presence of 50 µM of the soybean trypsin inhibitor (Trypsin inhibitor). **p<0.01. indicate a significant difference between different groups using the T-test. Data are represented as mean/± SD.

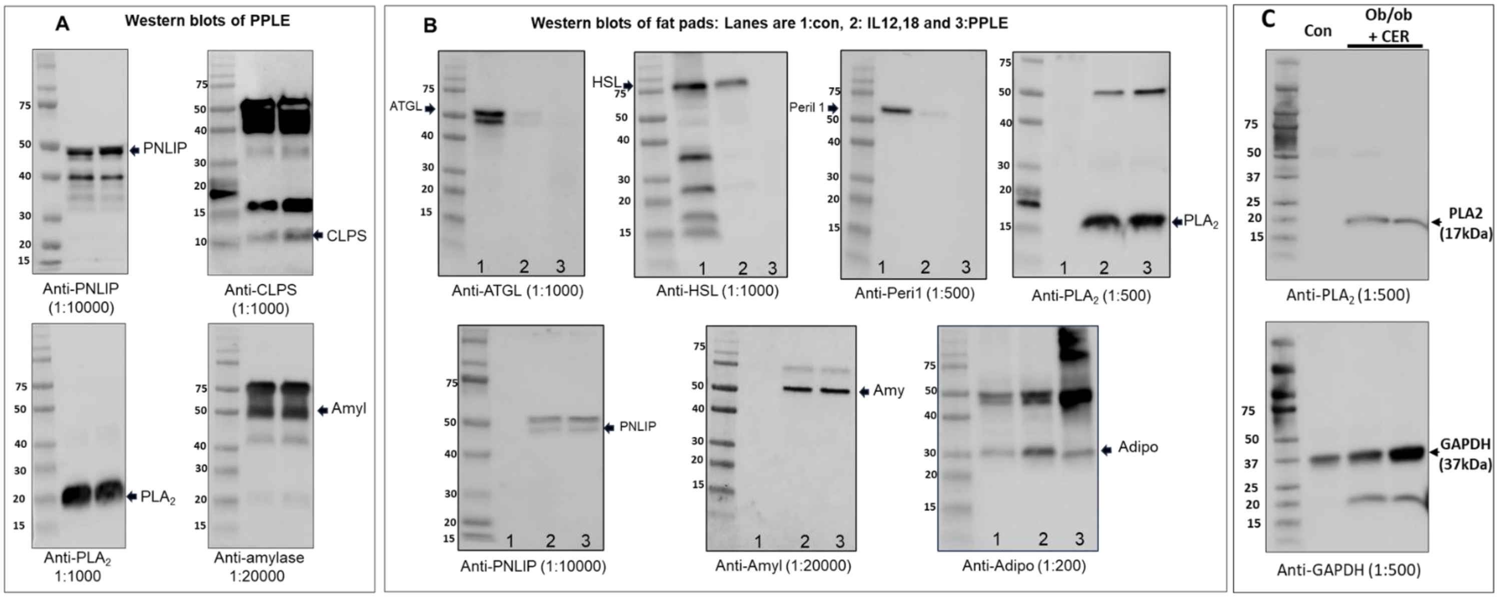


**Figure S11: Western blot full gel images showing detectable pancreatic enzymes in PPLE, fat pads of mice**. Related to Figure 3. **(A)** PNLIP, CLPS, $PLA_2$, and amylase in porcine pancreatic extract (PPLE). (**B**) Western blots for ATGL, HSL, perilipin 1 (Peri1), PNLIP, amylase (Amyl), and adiponectin (Adipo) bands in the fat pad of mice from control (Lane 1), treated with IL12;18 (Lane 2), and PPLE (Lane 3) models. (**C**) Western blot full gel image showing the leakage of $PLA_2$ into the fat pad of ob/ob treated with caerulein, the GAPDH was used as a control.

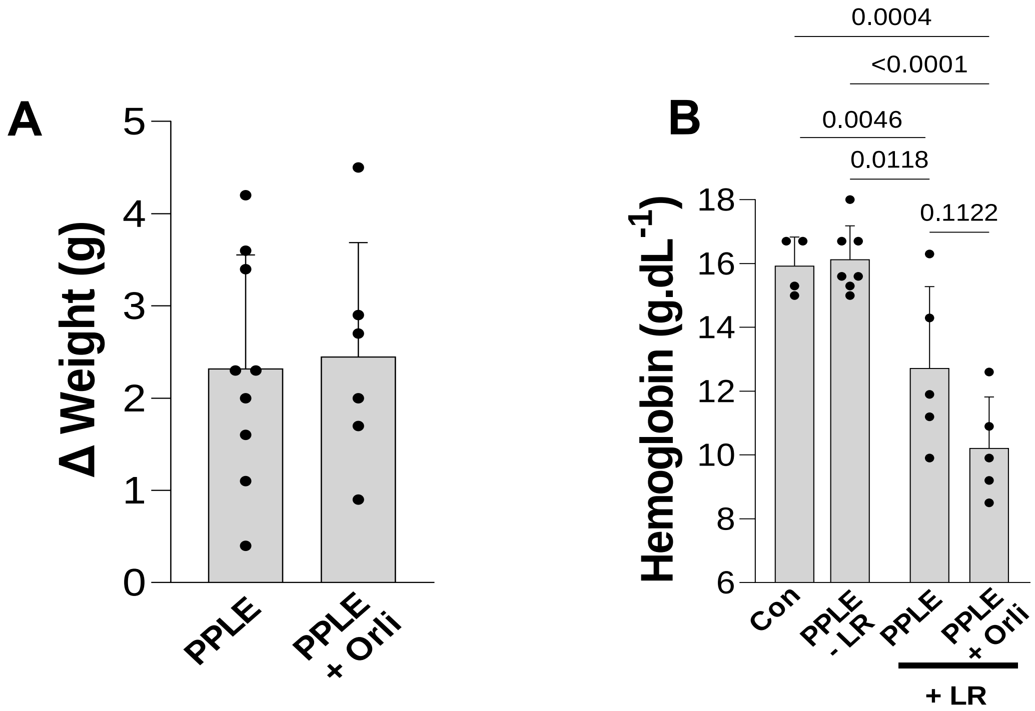


**Figure S12: Comparison of the difference in weights from baseline (A) and blood Hemoglobin at the time of necropsy (B) of the PPLE group vs. PPLE + orlistat group receiving Lactated Ringer's (LR) fluid supplementation.** Related to Figure 3. p-values show the significance between different groups. Note that both groups had a similar increase in weight and reduction in hemoglobin consistent when receiving LR, while the PPLE-LR group (B) had no reduction in hemoglobin. Thus, LR increased blood volume and caused hemodilution in both groups. Data are represented as mean/± SD.

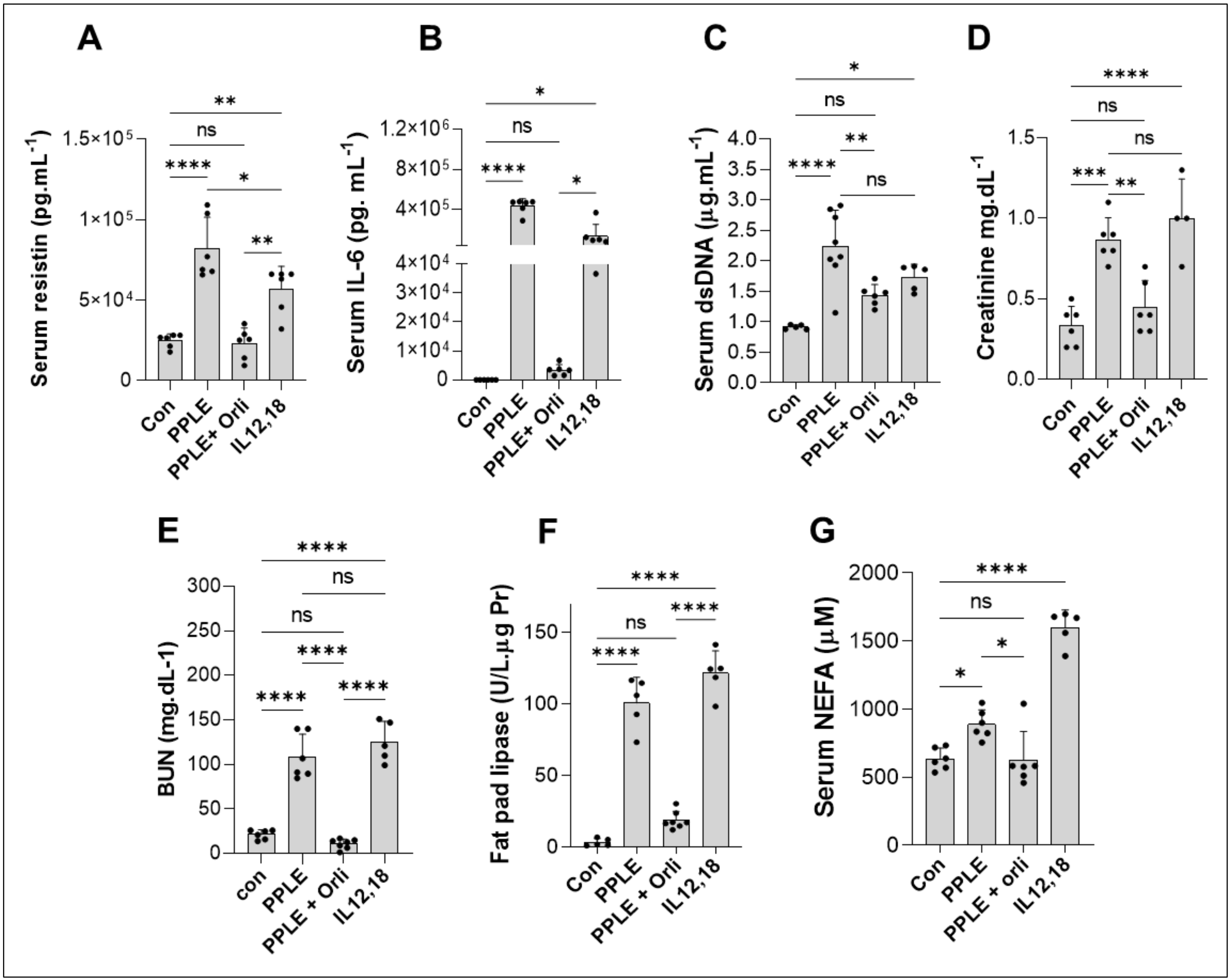


**Figure S13: (A-E) Serum biochemical parameters, cytokines, and organ failure parameters in mice given IL12/18 or PPLE (+/-orlistat).** Related to Figure 3. Serum Resistin (**A**), IL-6 (**B**), Serum dsDNA (**C**), creatinine (**D**), and BUN (**E**) in controls (con), and other mice treated by IL12/18 and PPLE (+/-orlistat). Pancreatic lipase activity (**F**) in fat pads and serum NEFA (**G**) of these mice at the time of necropsy. *p<0.05, **p<0.01, ***p<0.001; **** p<0.0001 indicate a significant difference between different groups using 1-way-ANOVA. Data are represented as mean/± SD.

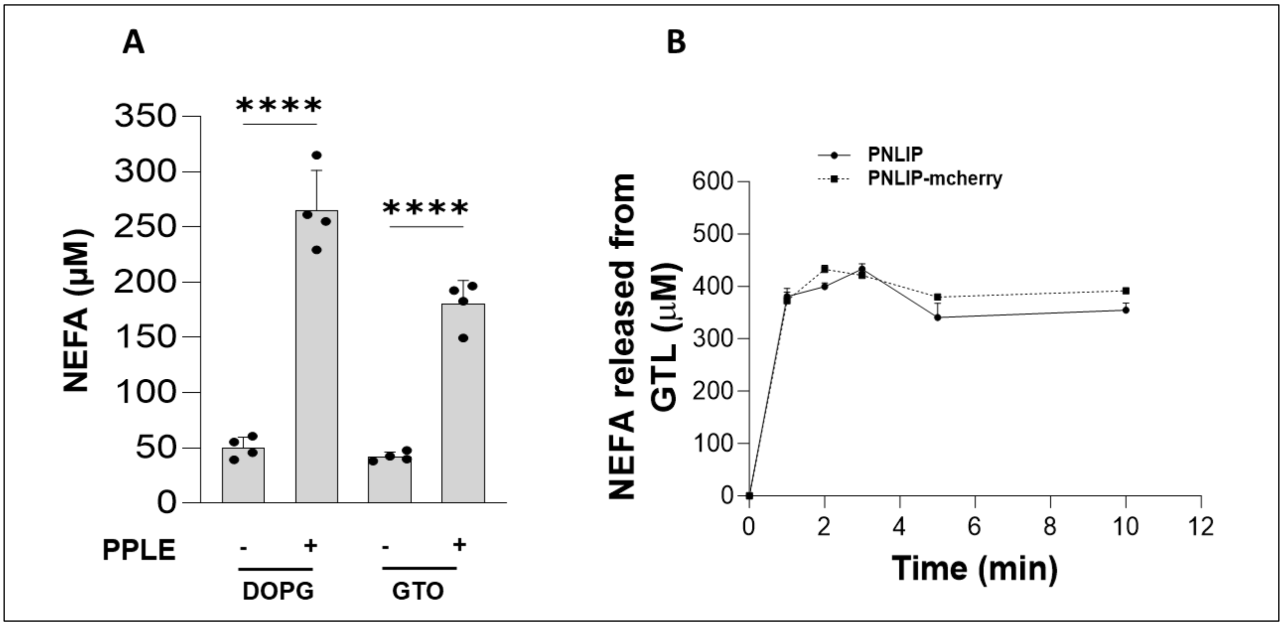


**Figure S14: NEFA release from lipids after different treatments.** Related to Figures 4 and 5. **(A)** NEFA release from 10 mM DOPG and 600μM GTO hydrolyzed by PPLE (0.5 mg.mL$^{-1}$). (**B**) Activity of PNLIP and PNLIPmcherry on GTL(600uM) as substrate. ****P<0.0001, indicates a significant difference between different treatments and significance determined by 1- way ANOVA. Data are represented as mean/± SD.

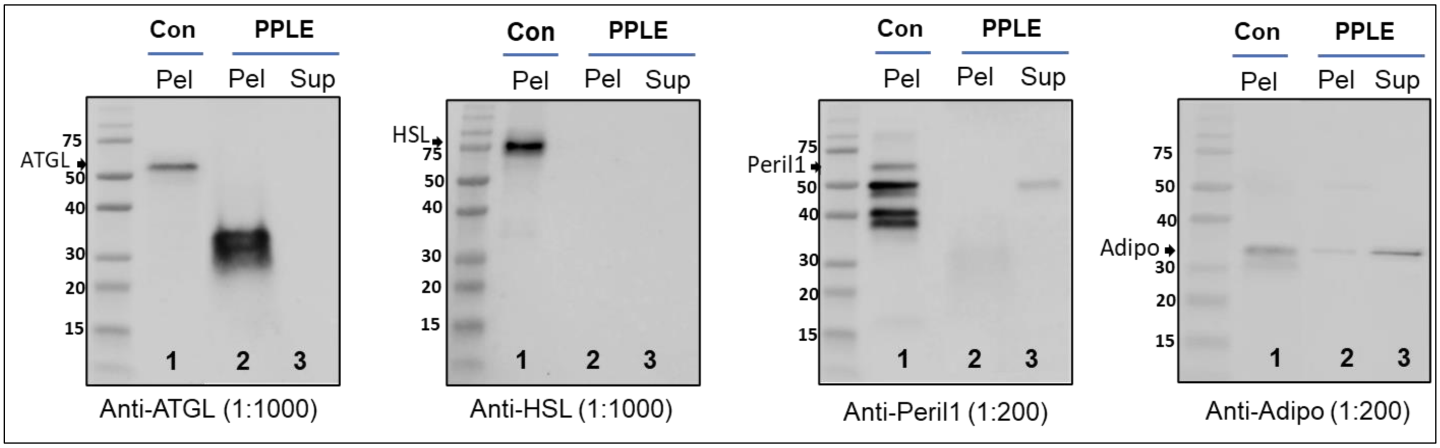


**Figure S15: Western blot (Full gel) comparing adipocyte proteins in different fractions:** Related to Figure 6. From left to right, these show ATGL, HSL, perilipin-1 (peri1), and adiponectin (Adipo) bands in 3T3-L1 pellet **(Pel)** and supernatant **(Sup).** Lane 1: cell lysate pellet, **Lane 2**: pellet of 3T3-L1 treated by PPLE, **Lane 3**: supernatant of 3T3-L1 treated by PPLE.

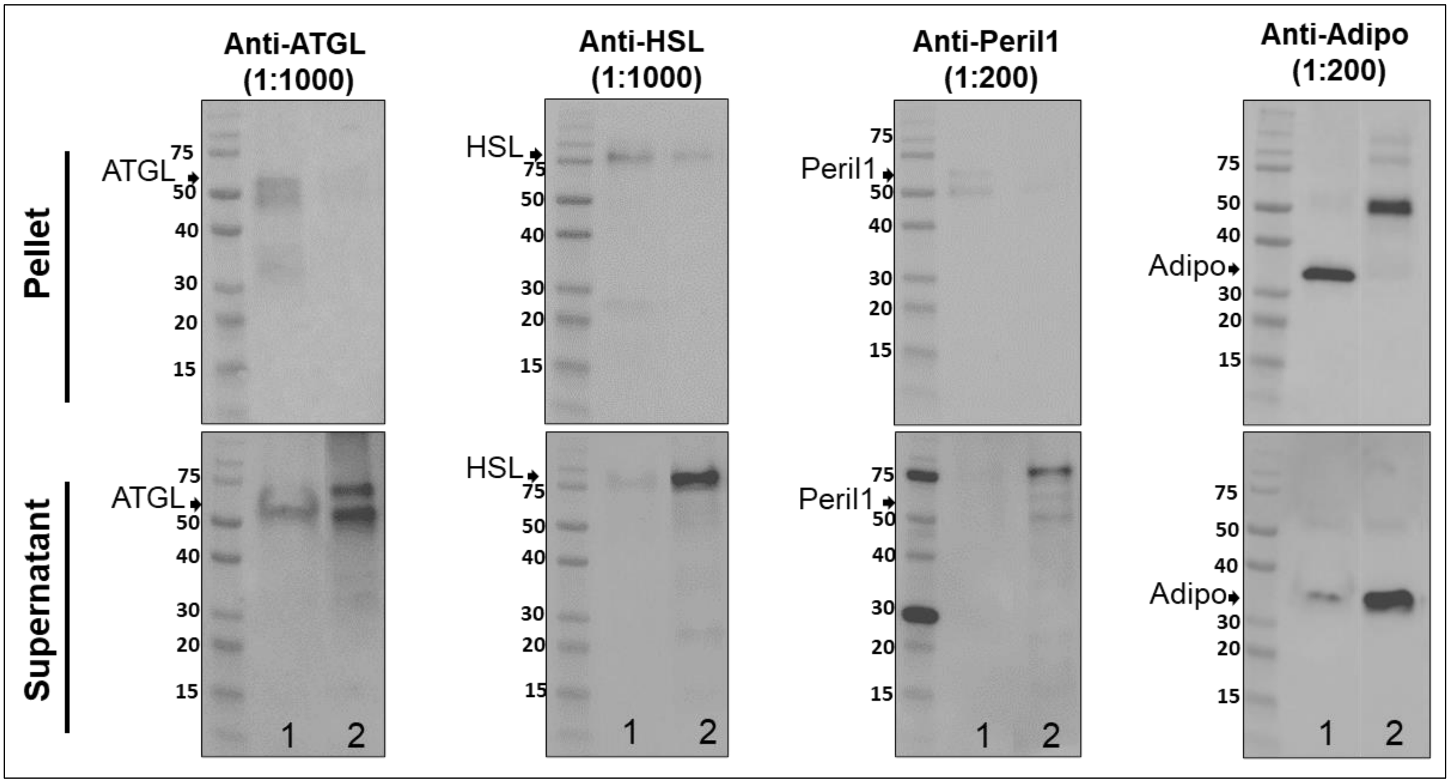


**Figure S16:** Western blot full gel images comparing detectable ATGL, HSL, Perilipin 1 (Peri-1), and adiponectin (Adipo) bands in the 3T3-L1 pellet and supernatant from 3T3-L1 control (**Lane 1**) and cells exposed to HbPLA$_2$ (**Lane 2**). Related to Figure 7. The arrows (➧) indicate the corresponding proteins.

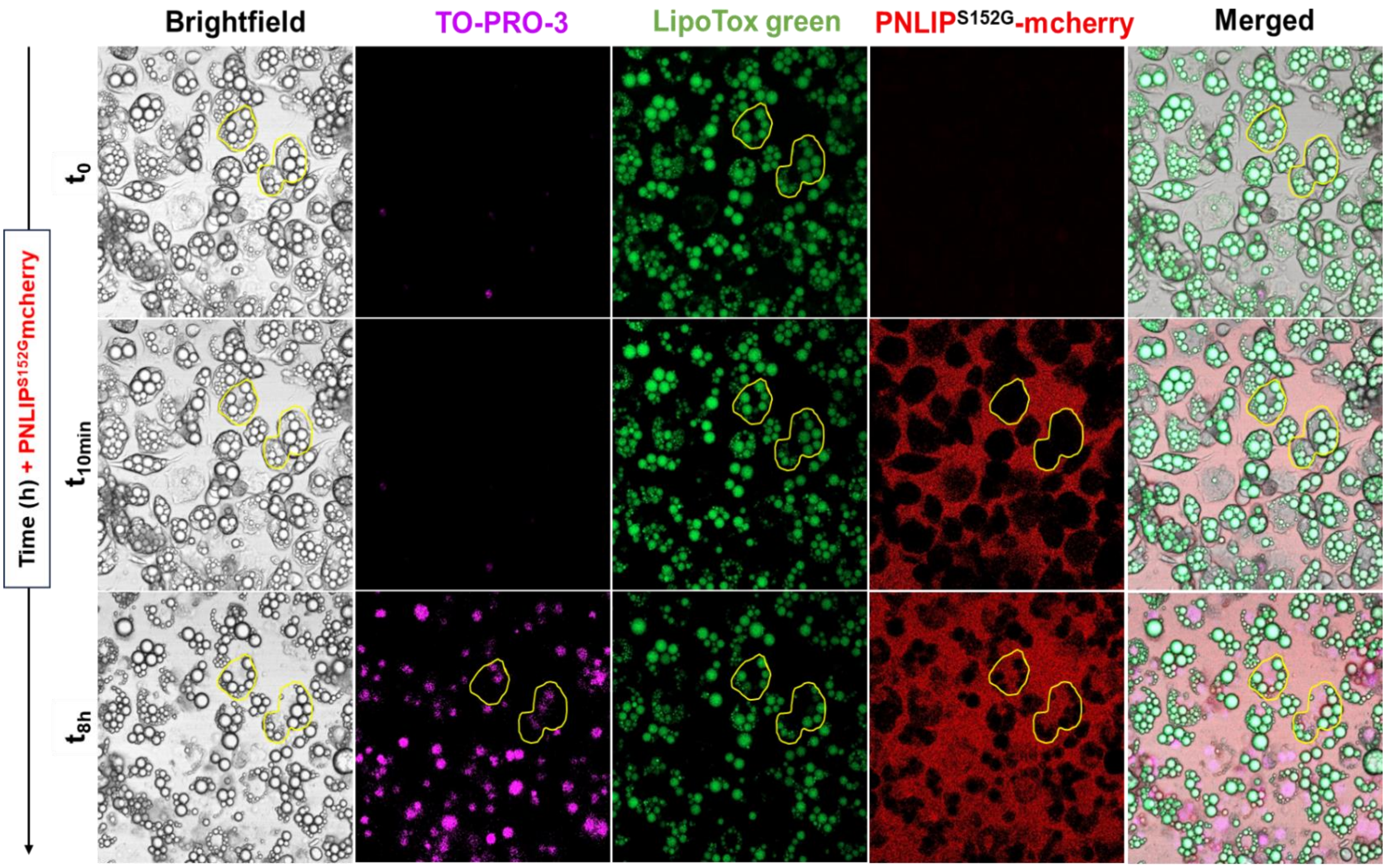


**Figure S17: Live imaging representative of 5 different experiments showing the cell death of 3T3-L1 cells by HbPLA$_2$ and the absence of lipolysis in the presence of the PNLIP inactive form (PNLIP$^{S152G}$mcherry).** Related to Figure 7. Cells were treated by these enzymes for 8h in HEPES media (pH 7.4) at 37°C.
