# Supplementary material for: Visceral fat lipolysis by pancreatic lipases worsens heart failure
Source: Cell Rep Med. 2025 Jun 2;6(6):102147. doi: 10.1016/j.xcrm.2025.102147 (PMC12208342; doi:10.1016/j.xcrm.2025.102147)
Supplement: Document S2. Article plus supplemental information [file mmc25.pdf]

Cell Reports
Medicine

Article

Visceral fat lipolysis by pancreatic lipases worsens heart failure

Graphical abstract

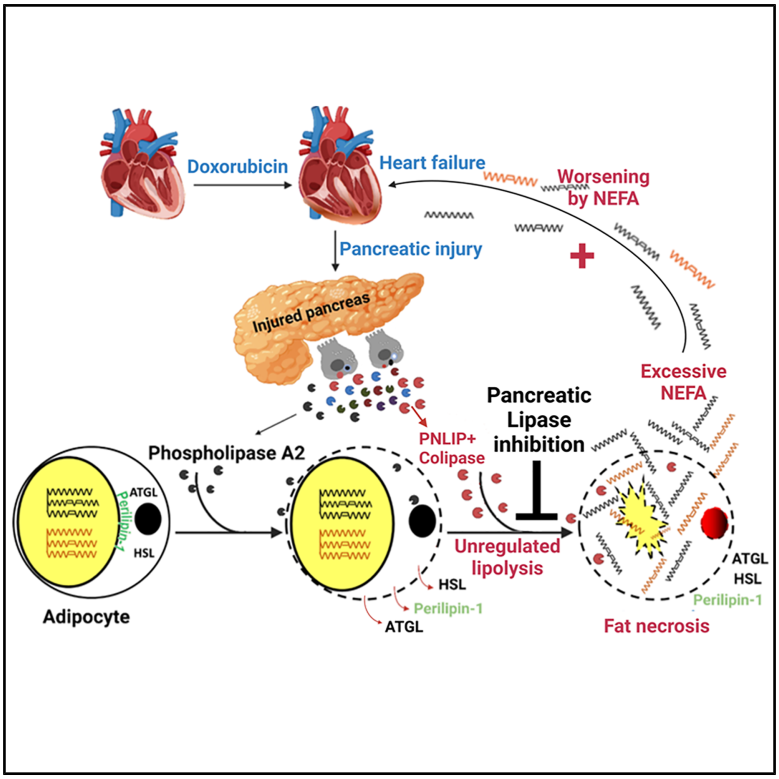

Authors

Nabil Smichi, Biswajit Khatua, Sergiy Kostenko, ..., Sarah Jahangir, Marek Belohlavek, Vijay P. Singh

Correspondence

singh.vijay@mayo.edu

In brief

Smichi et al. show that pancreatic enzymes leaked during pancreatic injury cause visceral adipocyte membrane damage and adipocyte lipase loss. The pancreatic lipase PNLIP enters adipocytes and rapidly hydrolyzes their lipid droplets, generating excessive non-esterified fatty acids that worsen heart failure. Pancreatic lipase inhibition halts this sequence and may be tested clinically.

Highlights

- Heart failure causes pancreatic injury and pancreatic enzyme leakage
- Pancreatic lipases and phospholipases damage membranes and enter adipocytes
- Excess fatty acids from unregulated lipid droplet hydrolysis worsen heart failure
- Reducing this fat necrosis by inhibiting pancreatic lipase improves heart failure

Smichi et al., 2025, Cell Reports Medicine *6*, 102147
June 17, 2025 © 2025 The Author(s). Published by Elsevier Inc.
https://doi.org/10.1016/j.xcrm.2025.102147

CellPress

Cell Reports Medicine

CellPress
OPEN ACCESS

Article

Visceral fat lipolysis by pancreatic lipases worsens heart failure

Nabil Smichi,[1] Biswajit Khatua,[1] Sergiy Kostenko,[1] Cristiane de Oliveira,[1] Bara El Kurdi,[2] Kalpit Himmatbhai Devani,[2] Shubham Trivedi,[1] Megan Summers,[1] Bryce McFayden,[1] Sarah Navina,[3,5] Krutika Patel,[1,6] Sarah Jahangir,[1,7] Marek Belohlavek,[1] and Vijay P. Singh[1,4,8,*]

[1]Department of Internal Medicine, Mayo Clinic, Scottsdale, AZ, USA
[2]Department of Medicine, East Tennessee State University, Johnson City, TN, USA
[3]Department of Pathology, University of Pittsburgh, Pittsburgh, PA, USA
[4]Department of Biochemistry and Molecular Biology, Mayo Clinic, Scottsdale, AZ, USA
[5]Present address: Clinpath Associates Arizona, Phoenix, Arizona, USA
[6]Present address: Vanderbilt University, Nashville, Tennessee, USA
[7]Present address: East Carolina University, Greenville, North Carolina, USA
[8]Lead contact
*Correspondence: singh.vijay@mayo.edu
https://doi.org/10.1016/j.xcrm.2025.102147

SUMMARY

Heart failure can be worse when associated with obesity, elevated serum pancreatic enzymes, elevated non-esterified fatty acids (NEFAs), or acute pancreatitis (AP). To understand this, here we study doxorubicin-induced heart failure, experimental AP, or pancreatic lipase-induced visceral fat necrosis in lean, genetically obese (ob/ob), or dual ob/ob pancreatic triglyceride lipase (PNLIP)-knockout mice. NEFA generation and resulting cardiac injury are measured. We note that ob/ob mice develop fat necrosis containing PNLIP and phospholipase A_2. This generates excess NEFAs that worsen cardiac injury, cause hypotension, and reduce survival. All these are prevented by PNLIP deletion or pharmacologic inhibition. Live imaging shows that phospholipase A_2 damages adipocyte membranes, resulting in PNLIP entry and leakage of adipocyte lipases. PNLIP hydrolyzes adipose triglyceride, generates NEFAs, and causes lipid droplet loss and adipocyte necrosis. Therefore, pancreatic injury can worsen antecedent heart failure by leaked PNLIP, causing excessive visceral adipose lipolysis. Inhibition of such lipolysis may improve heart failure outcomes.

INTRODUCTION

Patients with elevated serum pancreatic enzymes[1,2] or fatty acids[3,4] during heart failure or cardiac arrest[5] have worse outcomes. Recent studies show heart failure to be a cause of death in patients with acute pancreatitis (AP) after hospital discharge.[6] The pancreas is sensitive to ischemia,[7,8] with elevated serum lipase noted after aortic clamping[8] and frank pancreatitis diagnosed on autopsy in 20%–40% of cases after death from cardiac surgery or low-output heart failure.[9] Human AP universally involves fat necrosis,[10,11] and up to a third of patients with painless serum lipase elevation may have AP on imaging.[12,13] Therefore, while it is plausible, the mechanisms linking heart failure and AP are not known. Determining if pancreatic lipase inhibition can improve heart failure outcomes is important since such therapy has entered clinical trials (ClinicalTrials.gov ID NCT06080789).

Visceral fat increase in obesity is associated with increased progression[14] and worse outcomes in heart failure[15,16] and pancreatitis.[17] Human visceral fat averages 3–5 kg[18] (range 0.1–12 kg).[19] The pancreas weighs 40–200 g (average 90 g)[20] and is surrounded by visceral adipose tissue.[21] 80%–90% of an adipocyte's mass is triglyceride,[22] and 80%–90% of the pancreas' mass is exocrine,[23] which includes lipases and phospholipases.[24] Normally, the pyramid-shaped exocrine pancreatic acinar cells secrete these enzymes apically[25] via ducts into the duodenum, without contact with the basally located adipocytes[21,26,27] despite their close proximity. Similarly, under normal states, adipose triacylglycerols are stored in adipocyte lipid droplets (LDs)[28,29] without contact with pancreatic lipases. Normally, when energy demand is increased, adipocyte triglycerides are hydrolyzed in a regulated fashion into non-esterified fatty acids (NEFAs) and glycerol. LDs are cytoplasmic triglyceride-enriched organelles delimited by a monolayer of phospholipids and proteins, including perilipin1–3.[28] Over the last decade, considerable progress has been made in understanding LD biogenesis and regulated lipolysis by adipocyte lipases such as hormone-sensitive lipase (HSL) and adipocyte triglyceride lipase (ATGL).

However, during disease states like AP,[10,11,26] or low-flow states such as surgery[30] and heart failure,[9] the fat in close proximity to the pancreas may undergo rapid, uncontrolled lipolysis.[26,31] This rapid lipolysis, described as lipolytic fat necrosis, generates large amounts of NEFAs[26,32] in these collections. Such large amounts of NEFAs, when released into the

This is an open access article under the CC BY license (http://creativecommons.org/licenses/by/4.0/).

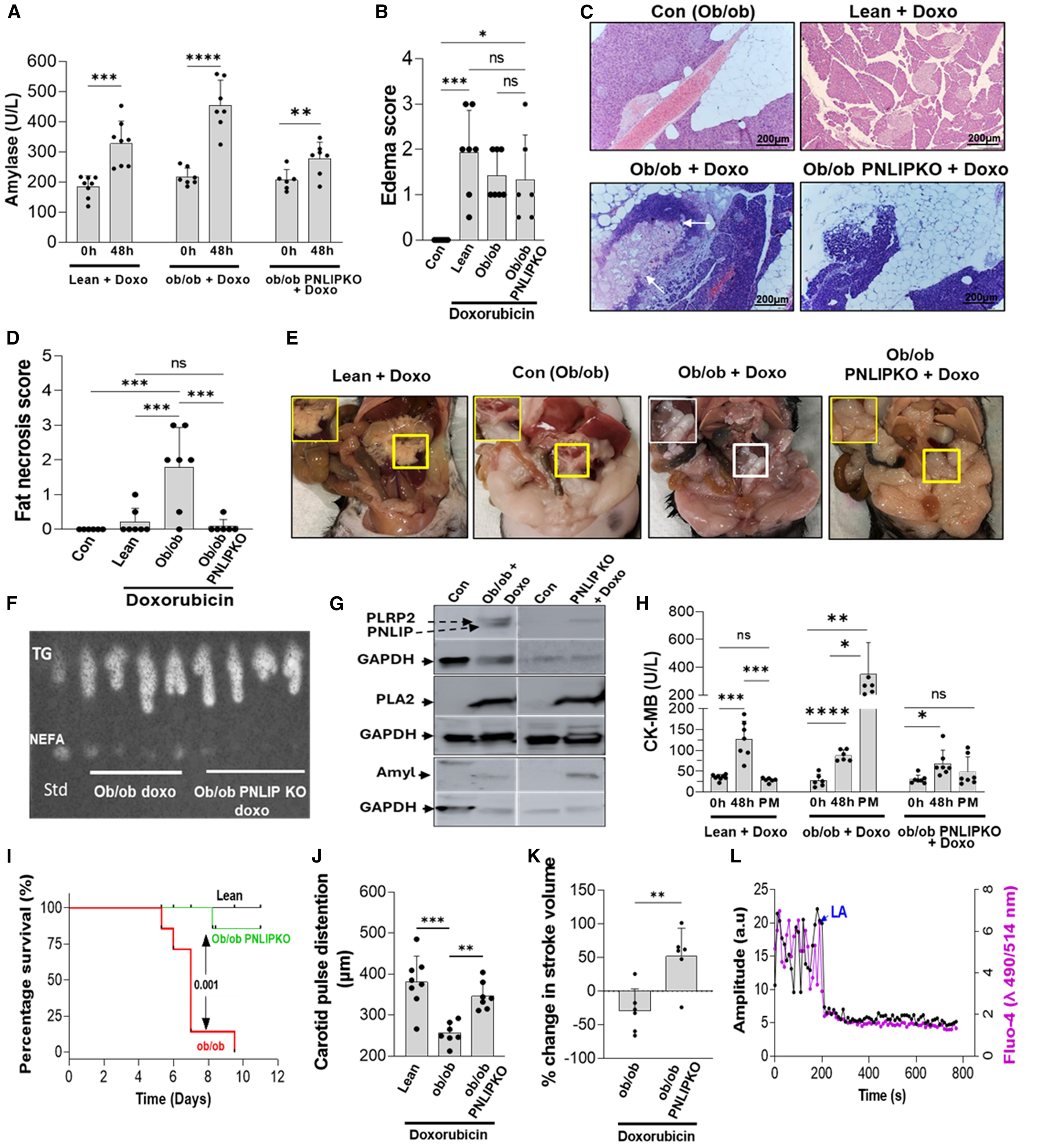

Figure 1. Pancreatic injury worsens antecedent heart failure via PNLIP-mediated fat necrosis

(A) Serum amylase was measured from lean, ob/ob, and ob/ob PNLIP-KO mice treated with doxorubicin.

(B) Edema was quantified from these mice.

(C) H&E staining of the pancreas and surrounding fat.

(D) Fat necrosis score in controls compared to lean, ob/ob, and ob/ob PNLIP-KO mice treated by doxorubicin.

(E) Gross appearance of fat pads at the time of necropsy. The fat pads of the lean mice treated by Doxo, control obese mice were compared to obese mice, and PNLIP-KO mice treated with doxorubicin (right) (*n* = 6).

(legend continued on next page)

circulation,[33,34] cause systemic inflammation and multi-system organ failure,[26,32] including acute kidney injury, shock, lung injury, and infections.[26,32,35,36]

We, thus, hypothesized that if the previously shown basolateral leakage during AP[25] occurred from pancreatic injury during heart failure,[8,37] this may necrose the surrounding visceral fat since fat necrosis is universally noted with pancreatic injury[38,39] on autopsy[10] and surgery.[11] We further hypothesized that such a leak during heart failure could cause excessive NEFA release. The pancreatic lipases[40] known to leak into fat include pancreatic triglyceride lipase (PNLIP),[32] which has ≈80% of pancreatic lipase activity,[41,42] PNLIP-related protein class 2 (PNLIPRP2),[32] and carboxyl ester lipase.[43] The latter is irrelevant since it requires high concentrations of bile acids (normally present in the duodenum) and cannot effectively hydrolyze long acyl chain triglycerides.[44] If our hypotheses were true, we aimed to understand the mechanisms by which pancreatic lipases enter adipocytes,[32] hydrolyze their triglycerides, and generate NEFAs, resulting in worse outcomes.

Before testing the hypothesis, we first verified that unexplained AP during heart failure was associated with worse outcomes in a nationwide inpatient sample (NIS) database study. To further test these hypotheses and understand the mechanisms, we induced heart failure in lean and obese mice using doxorubicin. Doxorubicin's clinical relevance lies in it being a widely used chemotherapy agent[45] for breast cancer, bone sarcoma, and leukemias. It causes cardiac dysfunction,[46–53] with cardiotoxicity unpredictably developing within a month[54] and progressing to 40% of patients without previous heart disease over 10 years.[50] Moreover, cardiotoxicity is worse in obesity[55] and is dose dependent, with >60% having echocardiographic abnormalities at the highest dose. On noting that obese mice with heart failure have an increase in pancreatic enzymes in visceral fat, fat necrosis, NEFA generation, and worsening heart failure like humans,[1,2,9] we studied the underlying mechanisms. For this, we first verified that pancreatic enzyme leak into visceral fat is indeed present in human AP (by comparing it to inflamed fat in diverticulitis) and rodent AP and that fat necrosis can cause cardiac injury irrespective of whether this fat necrosis is due to AP or pancreatic enzyme injection into fat. After noting this to be accurate, we studied how pancreatic enzymes can take over the lipolytic machinery of an adipocyte. Interestingly, we note multiple mechanisms of membrane damage (not the current focus) that cause the loss of adipocyte lipases and provide PNLIP access to the LD. These result in unregulated lipolytic fat necrosis with excessive NEFA generation, cytokine, and damage-associated molecular pattern (DAMP) release. Here, we detail the pathophysiology of these events in the context of worsening heart failure.

RESULTS

Pancreatic injury worsens antecedent heart failure via PNLIP-mediated fat necrosis

Based on the International Classification of Diseases nine codes 428.0–428.9 (see Figure S1A), between 2010 and 2014, there were 4,364,899 patients admitted with congestive heart failure (CHF) out of a total of 37,312,324 admissions in the NIS database. After exclusions (age <18 years of age, missing data on gender or mortality data, a diagnosis of chronic pancreatitis and pancreatic cancer), 4,329,481 patients with CHF remained. 27,017 of those had an additional diagnosis of AP (code 577.0). Patients with AP and CHF had higher mortality (6.9%) vs. non-AP patients with CHF (5.0%; $p < 0.0001$) despite being younger and having less diabetes mellitus, lower obesity, and a lower Charlson Comorbidity Index (Table 1). As shown in Table 2, hypertension did not contribute to mortality in this group. Using multivariate logistic regression analysis to adjust for odds ratios, as shown in Figure 1 and Table 2, mortality was more likely in patients with CHF with AP compared to patients with CHF without AP (odds ratio 1.57 [confidence interval (CI) 95% 1.54–1.61], $p < 0.0001$). We further stratified AP into AP without a clear cause, i.e., idiopathic AP (9,274 patients) and AP with known etiology (17,743 patients), and compared mortality. Mortality risk in patients with CHF with idiopathic AP was higher (OR 2.31 [CI 95% 2.24–2.38], $p < 0.0001$). Please note that the vast majority of human AP is diagnosed based on the presence of pain,[56] despite a substantial portion of AP being painless.[12] Such patients may have been missed in the NIS search. Thus, we studied if pancreatic injury could exacerbate heart failure.

Based on the aforementioned studies showing worse outcomes in heart failure with AP, and increased severity with visceral fat,[14–16] pancreatic lipase elevation,[1,2] and NEFA elevation,[3,4] we first studied an acute heart failure model in obese mice using doxorubicin, based on its rapid onset,[54] its clinical relevance,[46–53] worsening with obesity,[55] poorly understood variable outcomes, and induction without preexisting heart failure.[50]

This was induced in lean mice (C57bl/6), genetically obese (ob/ob) mice, and ob/ob mice with a genetic deletion of PNLIP (ob/ob PNLIP-KO mice), which have the same amount of visceral fat as ob/ob mice (see Figure S1B).

Doxorubicin caused isolated pancreatic edema (Figures S2 (lower panel) and S3A–S3C) in lean mice but did not cause hepatocyte, renal, lung, or splenic white pulp injury on TUNEL staining (see Figure S2). Doxorubicin also did not interfere with the secretion of amylase from pancreatic acini (see Figure S3D) or cause pancreatic acinar LDH leakage (see Figure S3E), making its direct toxic effects unlikely. Despite no direct evidence of injuring

(F) Thin-layer chromatography comparing the relative amount of NEFAs and triglyceride in these mice fat pads. Standards: TG, glyceryl trilinoleate and NEFA, linoleic acid.

(G) Western blot images comparing detectable PNLIP, PLA_2, amylase, and GAPDH bands in the fat pads of these mice.

(H) The CK-MB was measured from the serum of lean, obese, and PNLIP-KO mice at 0 h, 48 h, and postmortem.

(I) Survival percentage of ob/ob and ob/ob PNLIP-KO mice ($n = 8$) treated with doxorubicin.

(J and K) The carotid artery pulse distention at 120 h and the reduction in stroke volume (K) in these mice.

(L) Representative graph of live imaging of Fluo-4 AM-loaded human cardiomyocytes showing calcium oscillations (magenta) and contractions (black). Cardiomyocytes were treated with 150 μM linoleic acid (LA) at 200 s.

*$p < 0.05$, **$p < 0.01$, ***$p < 0.001$, and ****$p < 0.0001$ indicate a significant difference between separate groups using one-way ANOVA and t test. Data are represented as mean/± SD.

Table 1. Baseline characteristics of the NIS database patients admitted with congestive heart failure and those with CHF and AP

Variable	CHF without AP (%)	CHF with AP (%)	*p* value
Baseline characteristics			
Total patients	4,302,464	27,017	
Mean age (yrs.)	72.8	69.6	<.0001
Female sex	2,228,056 (51.8)	14,011 (51.9)	0.8074
Mean CCI	2.213	2.03	<.0001
Diabetes mellitus	1,844,872 (43.9)	11,277 (41.7)	0.0002
Hypertension	3,022,336 (70.2)	19,769 (73.2)	<.0001
Obesity	741,525 (17.2)	4,415 (16.3)	<.0001
Myocardial infarct	351,068 (8.2)	1,911 (7.1)	<.0001

Table 2. NIS data showing odds ratios of mortality in CHF associated with other risk factors, comorbidities, and acute pancreatitis

Variable	Mortality (odds ratio)	CI 95%	*p* value
Age	1.025	1.025–1.026	<.0001
Female gender	1.108	1.103–1.112	<.0001
White race	1.073	1.068–1.078	<.0001
CCI 1	1.111	1.104–1.119	<.0001
CCI 2	1.288	1.28–1.297	<.0001
CCI 3	1.615	1.605–1.625	<.0001
Known AP	1.573	1.54–1.607	<.0001
Idiopathic AP	2.31	2.24–2.382	<.0001
Hypertension	0.646	0.643–0.648	<.0001

CCI, Charlson Comorbidity Index.

the exocrine pancreas, doxorubicin caused a similar increase in serum amylase (Figure 1A) and pancreatic edema (Figures 1B and 1C) at 48 h in all three mouse strains. This was preceded by a reduction in stroke volume, left ventricular end-diastolic volume, and cardiac output at 24 h (which was transient in lean mice; see Figure S4) and associated with cardiac injury noted as higher serum levels of creatine kinase-MB isoform (CK-MB) (Figure 1H). These findings suggested that pancreatic edema and amylase leakage resulted from the preceding cardiac injury. At the time of necropsy, ob/ob mice given doxorubicin also had evidence of fat necrosis adjacent to the pancreas (Figures 1C and 1D). This was seen microscopically as a diffuse amorphous appearance in adipocytes (white arrows Figure 1C). Grossly, this fat necrosis appeared as white deposits in the pancreas abutting visceral fat of ob/ob mice given doxorubicin (Figure 1E; white rectangle) but not in ob/ob PNLIP-KO mice. Thin-layer chromatography showed NEFA generation in the visceral fat of ob/ob mice given doxorubicin, which was reduced in ob/ob PNLIP-KO mice (Figure 1F). This fat necrosis was paralleled with a smaller increase in serum NEFA in doxorubicin-given PNLIP-KO mice (510 ± 254 vs. 88 ± 288 mM, $p < 0.01$; data not graphed). Since these findings are consistent with lipolysis of triglycerides to fatty acids during fat necrosis, we examined the fat pads for pancreatic enzymes using western blotting (Figures 1G and S5). Fat necrosis of ob/ob mice given doxorubicin had phospholipase A_2 (PLA_2), amylase, PNLIP, and PNLIPRP2 (all of which are enriched in the pancreas), while fat pads of ob/ob PNLIP-KO mice did not have PNLIP (50 kDa band below 52 kDa band of PNLIPRP2; Figure 1G) as expected.

Doxorubicin-induced early cardiac injury in all groups was noted as increased serum CK-MB activity at 48 h (Figure 1H). CK-MB normalized by day 12 in all groups except in the ob/ob mice, in whom it increased further till they were moribund (Figure 1H), requiring euthanasia (mean 168 ± 39 h; red line, Figure 1I). Lean mice were electively sacrificed after 288 h, as were the 6/7 ob/ob PNLIP-KO mice given doxorubicin, only one of whom required early euthanasia (green line Figure 1I). Only Ob/ob mice given doxorubicin also had persistently reduced carotid artery pulse distention, reduced cardiac stroke volume (Figures 1J and 1K), and cardiac output (4.6 ± 2.9 mL/min lower than the baseline of 11.7 ± 2.9 mL/min, $p < 0.01$) till necropsy. These were normalized in ob/ob PNLIP-KO mice, who also had a 1.8 ± 3.6 mL/min cardiac output increase from baseline at the time of necropsy ($p < 0.01$). Figure S6 shows details of NEFA elevation, histologic evidence of cardiac injury, and echocardiographic parameters in ob/ob mice vs. other groups. A lower dose of doxorubicin (2.5 mg/kg/day × 6 days)[57–59] also showed reductions in stroke volume, end-diastolic volume, and cardiac output over 2 weeks in obese mice only (see Figures S7A–S7C) but did not require euthanasia.

To verify the cardiotoxic effects of NEFA, we exposed cardiomyocytes loaded with the cytosolic calcium-sensitive dye Fluo-4-AM to 150 μM linoleic acid (LA; Figure 1L), which is equivalent to previously noted LA concentrations *in vivo*.[36,60] This LA dose, as shown in the middle row of Figure S2, caused lung, kidney, liver, spleen (white pulp), and pancreatic injury. LA promptly stopped both the normal contractions (black line) and cytoplasmic calcium oscillations (purple line) in cardiomyocytes, supporting NEFA's role in the *in vivo* cardiac injury noted earlier. Since the cardiac injury in ob/ob mice was related to fat necrosis generating NEFA, we next looked for the enzymes that may mediate human fat necrosis in patients with pancreatitis.

Human fat necrosis is enriched in NEFA and pancreatic lipases in the absence of adipocyte lipases

Fat necrosis is universal in human pancreatitis.[10,61,62] Figures 2A and 2B are examples of computed tomography (CT) scans done 4 weeks apart showing the progression of normal visceral fat (Figure 2A) to fat necrosis (Figure 2B) in close proximity to the pancreas. The pancreas normally weighs 40–200 g (blue outline, Figure 2A), and the large area of fat necrosis (yellow outline, Figure 2B) is consistent with a substantial proportion of visceral fat (averaging 3–5 kg)[18,19] being necrosed. Histologically, on hematoxylin and eosin staining, fat necrosis in pancreatitis was surrounded by inflammation and stained an amorphous pink blush, which was positive for calcium (brown) on von Kossa staining (Figures 2C and 2D), consistent with lipolytically generated fatty acids binding calcium. This is unlike the normal triglyceride-containing round-oval adipocytes that are empty and von Kossa negative, as described previously.[61,63–65] On

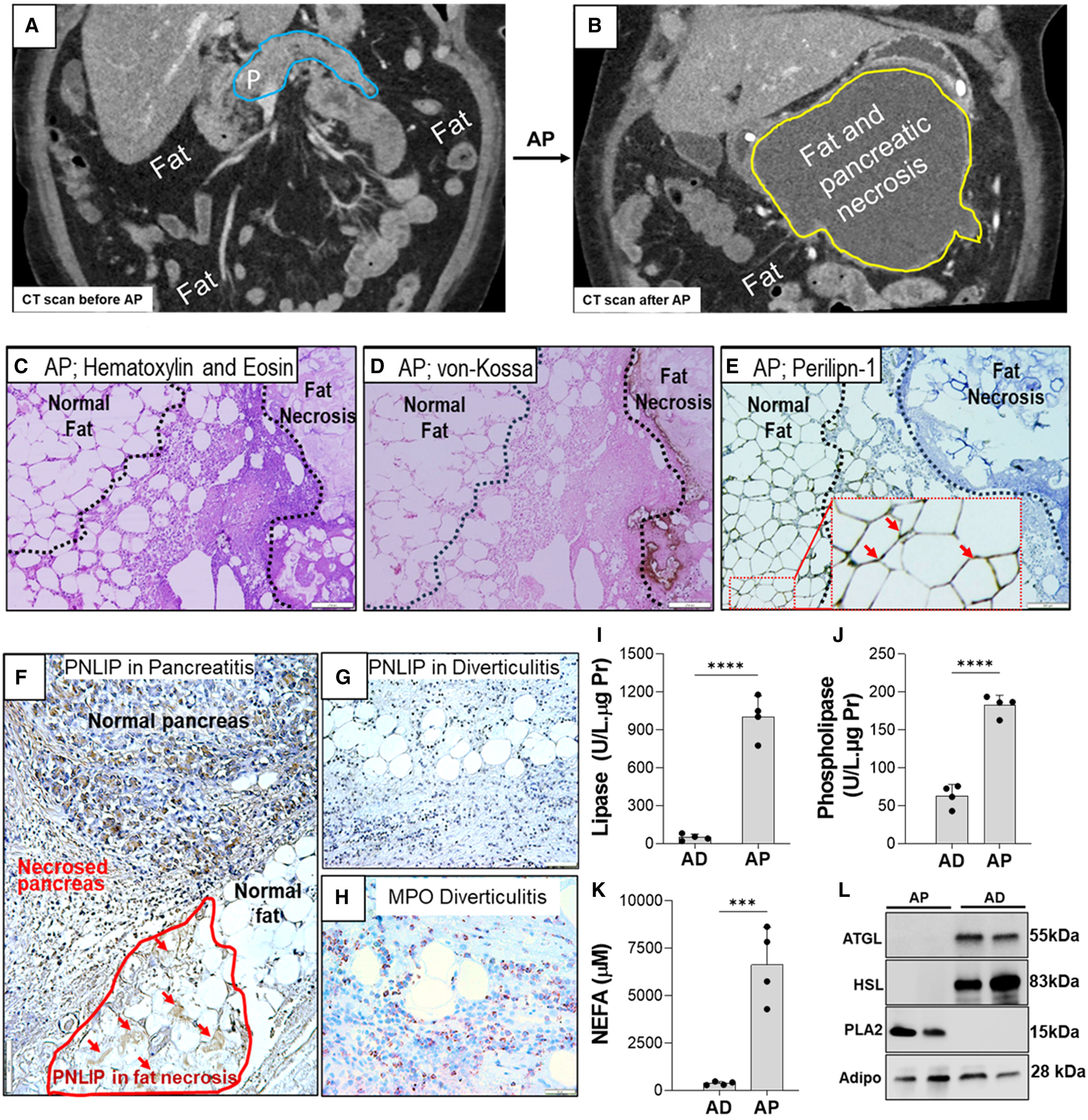

Figure 2. Human fat necrosis is enriched in NEFA and pancreatic lipases in the absence of adipocyte lipases

(A and B) Representative examples of cross-sectional CT scan images of the same patient before pancreatitis (A) and (B) of fat necrosis involving the pancreas that developed after AP (blue outline).

(C–E) Formalin-fixed paraffin-embedded sections of visceral fat of humans with acute pancreatitis showing normal fat (left side), necrosed fat (right side) stained with H&E (C), von Kossa for calcium (brown) (D), and immunohistochemistry (IHC) for perilipin-1 (E). Note that the amorphous-appearing fat necrosis has positive brown von Kossa staining and loss of perilipin-1 staining.

(F) IHC for PNLIP in human pancreatitis. PNLIP-positive brown areas showing fat necrosis are shown by red arrows, which are absent in normal fat.

(G) IHC for PNLIP in human diverticulitis.

(H) A representative example of human tissue stained for myeloperoxidase during acute diverticulitis.

(I–K) (I) Lipase, (J) phospholipase activities, and (K) NEFAs were measured in human acute diverticulitis (AD) and acute pancreatitis (AP) samples ($n = 5$).

(L) Western blot images comparing detectable adipose triacylglycerol lipase (ATGL), hormone-sensitive lipase (HSL), and adiponectin (Adipo) bands in these samples.

***$p < 0.001$, and ****$p < 0.0001$ indicate a significant difference between separate groups using the t test. Data are represented as mean/± SD.

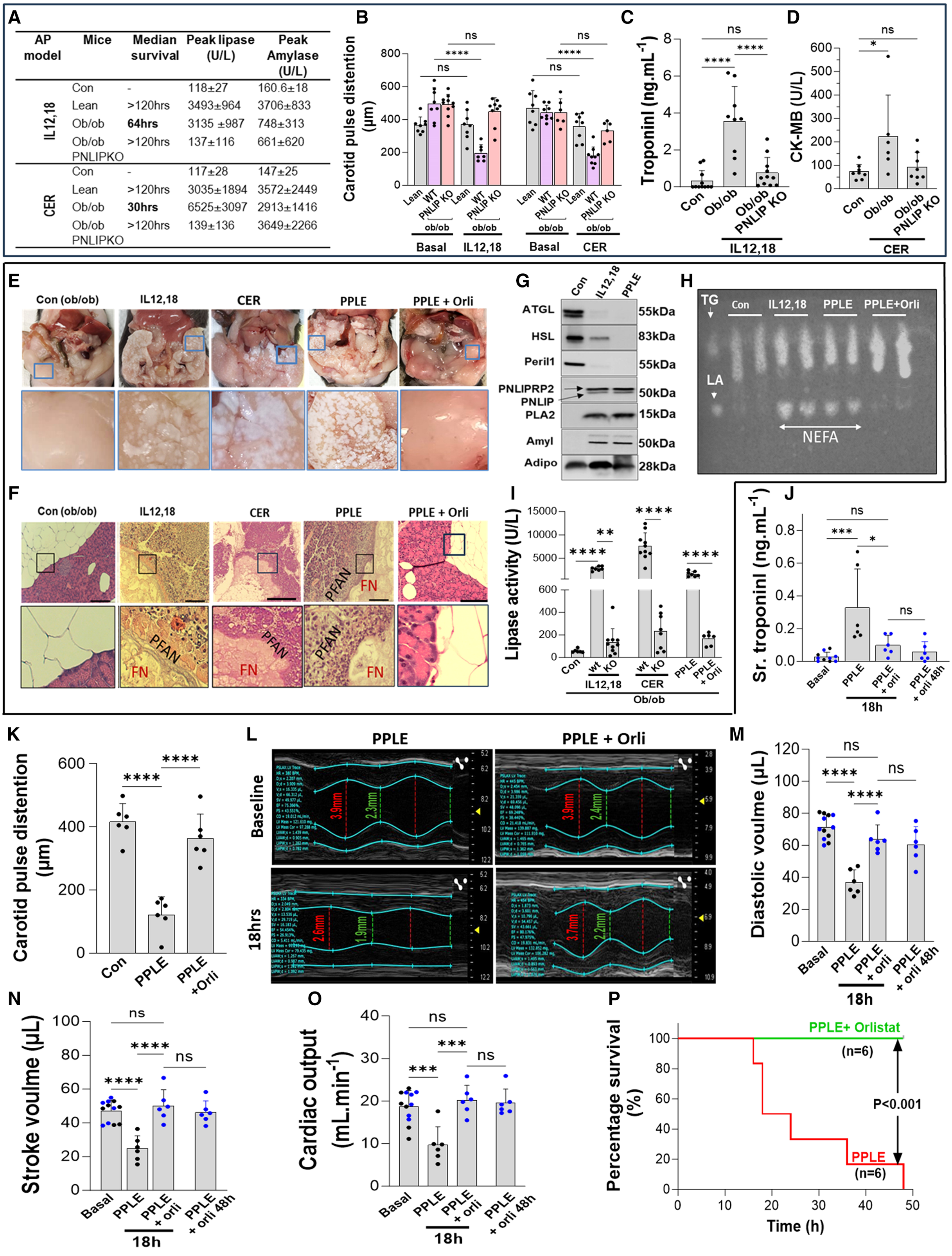

(legend on next page)

immunohistochemistry, fat necrosis stained negative for perilipin-1, unlike normal adipocytes, which stained brown, signifying positivity of intact LDs (red arrows Figure 2E, inset). As shown previously,[61,63,64] fat necrosis began at the borders of pancreatic necrosis (Figure 2F), likely due to leaked pancreatic enzymes being in contact with the adipose tissue, which is then necrosed. Unlike normal adipose, fat necrosis stained positive for pancreatic lipase (PNLIP; red outline, Figure 2F).[32] However, in acute diverticulitis, the adipocytes appeared normal and stained negative for PNLIP (Figure 2G) despite adipose inflammation (myeloperoxidase; MPO-positive red cells in Figure 2H). Therefore, during pancreatitis, unlike diverticulitis, there was histologic evidence of PNLIP leakage into visceral necrotic fat. We then compared pancreatic enzyme activity in the involved fat. As shown in Figures 2I and 2J, fat necrosis in AP contained significantly higher activity of pancreatic lipase and PLA_2 compared with diverticulitis and also had higher NEFA concentrations (6,636 ± 1,973 μM; Figure 2K). Interestingly, on western blotting, necrotic fat contained PLA_2 (Figures 2L and S8) but not endogenous adipocyte lipases ATGL and HSL, which were present in diverticulitis (Figure 2L). The adipocyte marker adiponectin was present in both diseases. Therefore, leakage of pancreatic lipases and phospholipases into visceral fat during fat necrosis in pancreatitis seemed to cause loss of adipocyte proteins while increasing triglyceride lipolysis and NEFA generation independent of adipocyte lipases.

Pancreatic lipase leakage into adipose causes fat necrosis and NEFA generation that causes cardiac injury

We next looked for evidence of PNLIP-mediated fat necrosis exacerbating cardiac injury in two previous well-established, published models of pancreatitis in obese and lean mice, i.e., cerulein pancreatitis and IL12,18-induced pancreatitis.[32] Inducing pancreatitis increased serum amylase in all mouse strains, in both models (Figure 3A). However, only ob/ob mice had reduced carotid pulse distention (red bars, Figure 3B) and increased cardiac injury biomarkers troponin-I or CK-MB in these models at necropsy (Figures 3C and 3D), with 0% survival at 5 days, unlike lean mice. AP, as shown previously,[32] occurred with fat necrosis containing PNLIP on western blotting and generating NEFAs (Figures 3E–3H). All these were prevented in PNLIP-KO ob/ob mice who had normal survival and did not increase serum lipase, consistent with PNLIP deletion (Figure 3A). To directly study the role of triglyceride lipolysis to NEFA, irrespective of baseline metabolic state, we induced IL12,18 AP in lean C57Bl6 mice with the triglyceride of LA (glyceryl trilinoleate, GTL; see Figure S9) alone or with orlistat. While AP caused a similar increase in lipase before GTL administration (see Figure S9A), GTL increased NEFAs and troponin-I levels and resulted in echocardiographic evidence of heart failure (see Figures S9B–S9H). All these were prevented by orlistat, supporting the role of NEFAs in worsening heart failure.

To further isolate pancreatic lipases' role in visceral fat necrosis irrespective of model, we introduced porcine pancreatic lipase extract (PPLE) into the gonadal fat pads of obese mice after neutralizing their trypsin activity (see Figure S10) and confirmed that the resulting necrosis contained relevant enzymes by western blotting. PPLE contained PNLIP, pancreatic colipase (CLPS), PLA_2, and amylase (see Figure S11A). Injecting visceral fat with PPLE caused fat necrosis similar to IL12,18 and cerulein pancreatitis,[26,32] which was noted as round white areas in the visceral fat (Figure 3E blue inset in the middle panel). These were prevented by the lipase inhibitor orlistat (Figures 3E and H). Histologically, on hematoxylin and eosin staining (Figure 3F), both PPLE and pancreatitis showed adipose inflammation and fat necrosis that appeared amorphous, chalky blue (Figure 3F) as observed previously.[26,27] These were similar to doxorubicin-treated ob/ob mice (Figures 1C and E) and necrosed the adjacent pancreas as described[26,27,32] (Figure 3F). Therefore, PPLE injections into fat pads caused fat necrosis similar to AP. We then evaluated if these were also biochemically similar to human pancreatitis.

After IL12,18 or PPLE treatment, western blotting of the fat pads showed that ATGL and perilipin-1 were undetectable, and HSL was only partially detectable (Figures 3G and S11B), similar to human fat necrosis (Figures 2E and 2L). Unlike

Figure 3. Pancreatic lipase leakage into adipose causes fat necrosis and NEFA generation that causes cardiac injury

(A and B) Median survival, peak lipase, amylase, and (B) carotid pulse distention from AP (IL12,18 and cerulein [CER]) in lean, ob/ob, and ob/ob PNLIP-KO mice (n = 6–10/group).

(C) Serum troponin I and (D) CK-MB of controls (Con) vs. ob/ob and ob/ob PNLIP KO with AP at necropsy.

(E) Representative gross appearance of fat pads (blue rectangle) at necropsy. The fat pads of the control obese mice (left panel) were compared to those given IL12,18, CER, PPLE, and PPLE + orlistat (right).

(F) Representative H&E-stained images of the pancreas and surrounding fat. Note the replacement of clear adipocytes in controls by amorphous bluish-pink deposits consistent with fat necrosis (FN) in ob/ob treated by IL12,18, cerulein, PPLE, and PPLE + orlistat. Also, note pancreas necrosis adjacent to FN, termed "peri-fat" acinar necrosis (PFAN). Scale bars: 200 μm.

(G) Western blot images comparing ATGL, HSL, perilipin-1 (Peri-1), PNLIP, PLA_2, amylase (Amyl), and adiponectin (Adipo) bands in ob/ob fat pads.

(H) Thin-layer chromatography comparing NEFA and triglycerides in the mice fat pads. Standards: TG, glyceryl trilinoleate; NEFA, linoleic acid.

(I) Serum lipase in control mice (con), compared to PPLE and PPLE + orlistat (Orli) ones, and treated by IL12,18 and CER AP.

(J) Troponin I in tail vein serum samples collected at the indicated times from PPLE and PPLE + orlistat-treated mice.

(K) Carotid pulse distention in control mice vs. PPLE and PPLE + orlistat-treated mice.

(L) M-mode echocardiographic views of the heart showing the outline of the endocardial and epicardial borders (blue) of the left ventricle. Dashed lines show numerical diameter in diastole (red) and systole (green).

(M–O) (M) Diastolic and (N) stroke volumes (μL) and (O) cardiac output in mice before and after the PPLE and PPLE + orlistat injections.

(P) Percentage survival in PPLE and PPLE + orlistat-treated mice (n = 6/group).

$^{**}p < 0.01$, $^{***}p < 0.001$, and $^{****}p < 0.0001$ indicate a significant difference between these groups compared to the control as determined by one-way ANOVA. Data are represented as mean/± SD.

adipocyte enzymes, PNLIP and PNLIPRP2,[66] PLA_2, and amylase were present in all three treatments and absent in controls (Figures 3G, S11B, and S11C). On thin-layer chromatography of fat pads, the normal adipose triglycerides (Figure 3H) were hydrolyzed to NEFAs after PPLE or IL12,18 treatment (Figure 3H), similar to doxorubicin-treated ob/ob mice (Figure 1F). Consistent with gross and histologic protection from fat necrosis by orlistat (Figures 3E and 3F), it also prevented the generation of NEFA (Figure 3H). PPLE-induced fat necrosis also increased serum lipase (Figure 3I)[32] as shown previously and was prevented by orlistat. PPLE increased troponin-I in tail vein samples before euthanasia (Figure 3J). PPLE reduced carotid artery pulse distention by >50% reduction (Figure 3K). On echocardiography (Figures 3L–3O; Video S1 [baseline], Video S2 [PPLE at 18 h]), PPLE reduced left ventricular end-diastolic volumes, stroke volumes, and cardiac output/minute. This cardiac injury occurred despite adequate fluid supplementation with Lactated Ringer's, as seen in both PPLE alone and PPLE + orlistat-treated mice, which had a similar weight gain of 1–3 g and hemodilution (see Figures S12A and S12B). All these were again normalized by orlistat (Figures 3J–3O; see Video S3 [baseline], Video S4 [PPLE + orlistat, 18 h]). Therefore, hypotension and cardiac failure occurred despite sufficient fluids and blood volume and were not solely due to vascular leak. Overall, this PPLE-induced cardiac injury and hypotension significantly reduced survival, all of which were prevented by orlistat (Figures 3K–3P). Both PPLE and IL12,18-induced AP models also caused systemic inflammation and organ failure (Figures S13A–S13E) as also shown with cerulein previously.[32] Both models increased fat pad lipase activity and NEFA release into the serum (Figures S13F and S13G) and were prevented by orlistat or genetically deleting PNLIP, as shown previously.[32] Therefore, since NEFA generation from fat necrosis from three different modes—doxorubicin, AP, and PPLE injection—worsened cardiac injury and function, we next studied how pancreatic enzymes enter adipocytes and cause fat necrosis and NEFA generation.

Phospholipases hydrolyze membrane phospholipids causing cell injury

We next studied the roles of phospholipase and lipase during fat necrosis *in vitro*. Hydrolysis of the phospholipid substrate dioleoyl-phosphatidylcholine (DOPC) by equimolar recombinant honeybee venom PLA_2 ($HbPLA_2$) was 3–4 times more than PLA_2 group 1B (PLA_2, Figure 4A). PLA_2, present in PPLE, was completely inhibited by varespladib (var-Na; Figure 4A). Since several pancreatic enzymes have PLA_2 activity,[67,68] we subsequently used the more potent $HbPLA_2$.

We then compared the specificity of $HbPLA_2$ to recombinant human pancreatic lipase (PNLIP) protein fused to mCherry (PNLIPmCherry; described in the methods section under Proteins). Please note that mCherry does not affect PNLIP's activity (see Figure S14B). Consistent with their roles, 1 μM $HbPLA_2$ hydrolyzed DOPC >10 times more than 1 μM PNLIP + CLPS (Figure 4A), while the latter hydrolyzed the triglyceride of LA (GTL; 600 μM) more (Figure 4B). The same was noted for oleic acid's triglyceride (GTO; data not shown). PNLIPmCherry was completely inhibited by orlistat but not by the phospholipase inhibitor var-Na (Figure 4B). We thus studied these recombinant enzymes, the S152G inactive mutant of PNLIP or PPLE ± pharmacologic inhibitors in cellular systems.

We first examined membrane injury in non-LD-containing HeLa cells. Live imaging showed that $HbPLA_2$ increased TO-PRO-3 uptake, consistent with membrane permeabilization from $HbPLA_2$ phospholipase activity (Figure 4C; see Videos S5 (control) and S6; blue line, Figure 4E). However, PNLIPmCherry + CLPS (further referred to as "lipase" in figures; Figure 4D) addition increased red fluorescence around the cells immediately (Figure 4D, downward arrow; see Video S7). After 3 h, this minimally increased TO-PRO-3 uptake compared to untreated cells (red vs. black line, Figure 4E).

Consistent with the aforementioned findings, $HbPLA_2$ increased lactate dehydrogenase (LDH) leakage to 45% ± 5% above controls (Figure 4F), vs. 9% ± 7% leakage with lipase (Figure 4F). Thin-layer chromatography (Figure 4G) showed complete lipolysis of membrane phospholipids by $HbPLA_2$, while lipase did so weakly (Figure 4G). Furthermore, $HbPLA_2$ increased double-stranded DNA (dsDNA) release from cells more than lipase treatment (Figure 4H). Overall, these suggest that, unlike PNLIP + CLPS (which has minimal phospholipase activity), $HbPLA_2$ efficiently hydrolyzes HeLa cell membrane phospholipids, thus, allowing leakage of macromolecules like LDH and dsDNA.

Lipolytic and phospholipase activity in PPLE causes cell injury

Pancreatic necrosis and PPLE (Figures 2, 3G, and 5A) both contain lipases and phospholipases, including PLA_2, PNLIP, and PNLIPRP2, which also has phospholipase activity. PPLE hydrolyzed both phospholipids [DOPC and DOPG; 1,2-dioleoyl-*sn*-glycero-3-phospho-(1′-rac-glycerol); Figures 5A andS14A] and triglycerides (GTL and GTO) (Figures 5B and S14A). The PLA_2 inhibitor (var-Na) inhibited DOPC and GTL hydrolysis by 77% ± 1% and 15 ± 0.6% (Figures 5A and 5B). The lipase inhibitor, orlistat, inhibited DOPC and GTL hydrolysis by 39% ± 2% and 67% ± 0.4%, respectively (Figures 5A and 5B). These findings are consistent with PPLE containing dual lipase and phospholipase activity, like pancreatic PNLIPRP2.[69,70]

On thin-layer chromatography of HeLa cells, PPLE reduced glycerophospholipids with all head groups, including glycerol (PG), ethanolamine (PE), serine (PS), and choline (PC) (Figure 5C), suggesting significant membrane phospholipid hydrolysis. Compared to untreated cells (see Video S8), PPLE (0.5 μg mL^{-1}) increased TO-PRO-3 uptake on imaging (Figures 5D and 5E; Video S9) along with loss of membrane integrity and morphology (merged images, lower row; Figure 5E), consistent with membrane phospholipids hydrolysis. While var-Na reduced TO-PRO-3 uptake by 24%, orlistat reduced TO-PRO-3 uptake by 53% (Figures 5D–5F and 5G; Videos S10 and S11). Thus, the lipase, PNLIPRP2, and phospholipase activities in PPLE may have redundant roles in damaging cell membranes. This is further supported by partial reductions in LDH leakage from 29% ± 0.8% to 17% ± 4% by var-Na and to 12% ± 2% by orlistat (Figure 5H), which parallel dsDNA release (Figure 5I).

PPLE mediates adipocyte fat necrosis *in vitro*

To simulate fat necrosis *in vitro*, 3T3-L1 adipocytes were exposed to PPLE for 7 h. PPLE released ATGL and HSL from

CellPress
OPEN ACCESS

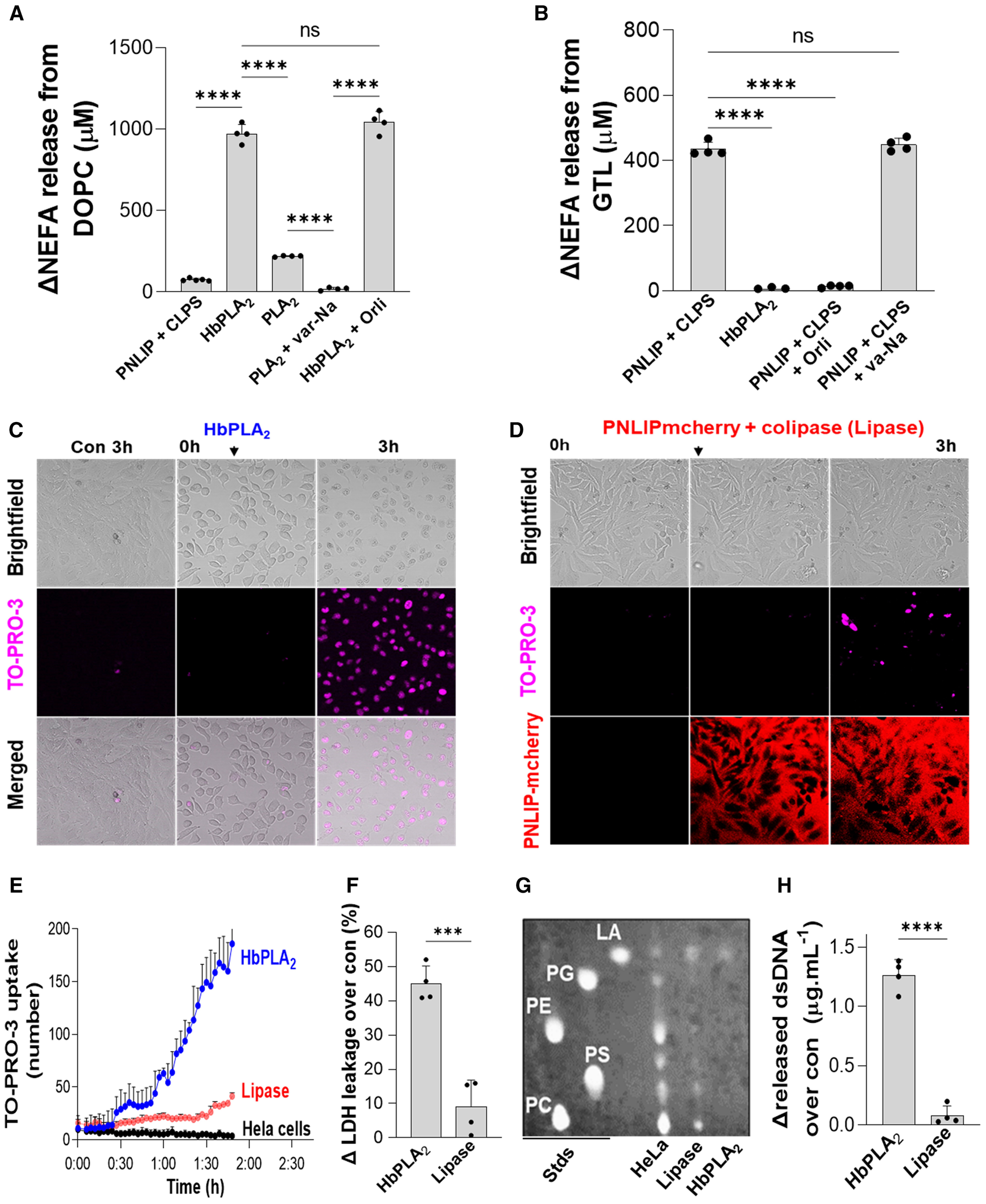

Figure 4. Effects of recombinant PNLIPmCherry and $HbPLA_2$ proteins on triglyceride, phospholipid hydrolysis, and HeLa cell injury
(A and B) The NEFA release by pure PNLIPmCherry (1 μM) with colipase, $HbPLA_2$ (0.6 μM), and PLA_2 (0.6 μM) was determined by hydrolysis of DOPC (10 mM) ($n = 4$) (A) and GTL (600 μM) (B). Statistical analysis was performed using one-way ANOVA.
(C and D) Live imaging of HeLa cells showing the effect of $HbPLA_2$ (C) and PNLIPmCherry + CLPS (referred to as "lipase" later) (D) on TO-PRO-3 uptake.
(E and F) Quantification of TO-PRO-3 uptake (E) and LDH leakage (F) is shown.
(G) TLC shows the breakdown of phospholipids from the HeLa cell membrane treated by lipase and $HbPLA_2$. Standards: NEFA (linoleic acid), and the diloeyl forms of PG (phospathidyl glycerol), PE (phosphatidyl ethanolamine), PS (phosphatidyl serine), and PC (phosphatidyl choline).
(H) The dsDNA released in the medium from HeLa treated by $HbPLA_2$ and lipase were determined ($n = 4$).
$^{***}p < 0.001$ and $^{****}p < 0.0001$ indicate a significant difference between these groups determined by one-way ANOVA and t test. Data are represented as mean/± SD.

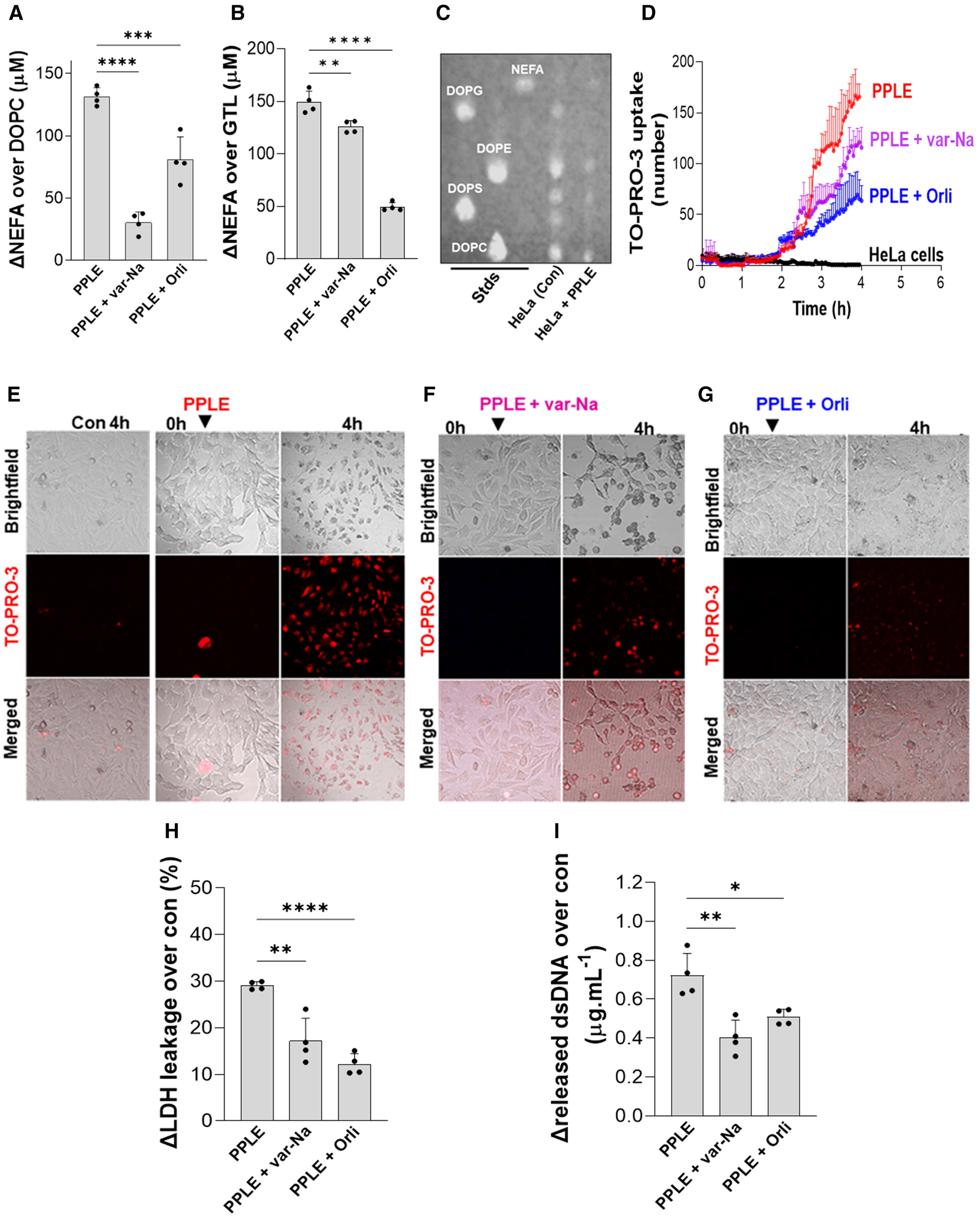

Figure 5. Lipolytic and phospholipase activity in PPLE causes HeLa cell injury

(A and B) PPLE (0.5 mg mL^{-1}) induced hydrolysis of DOPC (10 mM) (A), and GTL (600 μM) ($n = 4$) (B) was determined by measuring the increase of NEFA in the medium.

(C and D) TLC shows the breakdown of phospholipids from the HeLa cell membrane treated by lipase and $HbPLA_2$. Standards: NEFA (linoleic acid), DOPG, DOPE, DOPS, and DOPC. TO-PRO-3 uptake (D) of HeLa treated by PPLE, PPLE + var-Na, and PPLE + orlistat ($n = 4$).

(E–G) Live imaging ($n = 4$) showing the effect of PPLE, (F) PPLE + var-Na, and (G) PPLE + orlistat on HeLa cell death.

(H and I) LDH leakage (H) and the dsDNA (I) released in the medium from HeLa treated by PPLE, PPLE + var-Na, and PPLE + orlistat ($n = 4$) was also determined. *$p < 0.05$, **$p < 0.01$, **$p < 0.001$, and ****$p < 0.0001$ indicate a significant difference between separate groups using one-way ANOVA. Data are represented as mean/± SD.

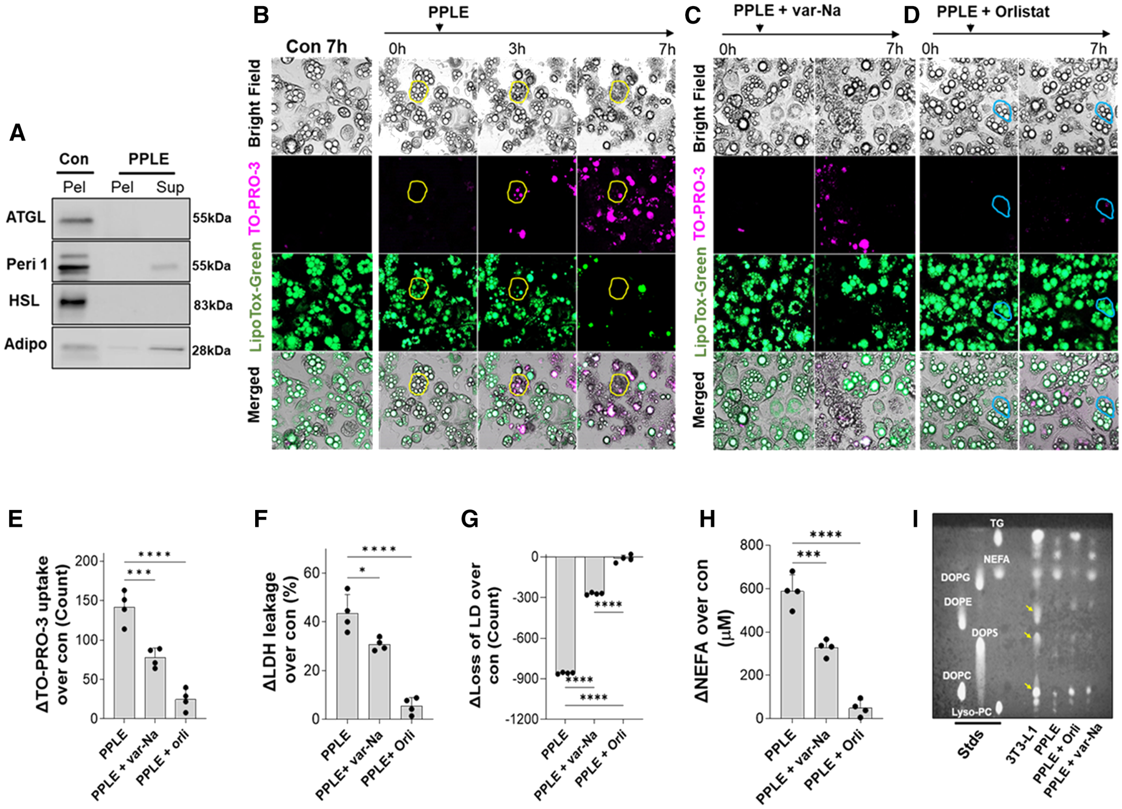

Figure 6. PPLE mediates adipocyte fat necrosis *in vitro*

(A) Western blot images comparing detectable ATGL, HSL, perilipin-1 (Peri-1), and adiponectin (Adipo) bands in the 3T3-L1 pellet (Pel) and supernatant (Sup) treated by PPLE.

(B) Live imaging of 3T3-L1 treated by PPLE, showing cell death (yellow outline), TO-PRO-3 uptake, and LD evolution.

(C–H) Effect of var-Na and orlistat (blue ovals) (D) on lipolysis of 3T3-L1 LD ($n = 4$). TO-PRO-3 uptake was quantified using ImageJ (E), and the LDH leakage (F) from 3T3-L1 treated by PPLE was also determined. LD lipolysis was quantified (G), and the NEFA release (H) was measured.

(I) TLC showing the breakdown of phospholipids from 3T3-L1 cells treated by PPLE. Standards: NEFA (linoleic acid), DOPG, DOPE, DOPS, DOPC, and Lyso-PC. *$p < 0.05$, ***$p < 0.001$, and ****$p < 0.0001$ indicate a significant difference between separate groups ($n = 4$ per group) using one-way ANOVA. Data are represented as mean/± SD.

cells (Figures 6A and S15), which were degraded, whereas perilipin-1 and adiponectin were released intact into the media (Figure 6A), similar to human and mouse fat necrosis (Figures 2L and 3G). We then analyzed the TO-PRO-3 uptake in 3T3-L1 cells (Figure 6B middle row; Videos S12 (control) and S13). TO-PRO-3 uptake started after 3 h and increased with time to reach its maximum at 7 h (Figure 6B; Video S13). The loss of LDs (yellow outline, Figure 6B) was seen as the disappearance of LipidTOX green (Figure 6B, third lane), which paralleled the TO-PRO-3 uptake, LDH leakage, and NEFA release (Figures 6B and 6E–6H). Var-Na reduced PPLE-induced TO-PRO-3 uptake (Figures 6C and 6E; Video S14) and LD loss (Figure 6G), consistent with a phospholipase in PPLE contributing to fat necrosis.

Orlistat provided greater protection from PPLE (Figure 6D; Video S15), with only a few cells showing TO-PRO-3 uptake (blue outline, Figure 6D) while profoundly reducing LDH leakage, LD loss, NEFA generation (Figures 6E–6H), and PPLE-induced conversion of triglycerides into NEFA on thin-layer chromatography (Figure 6I). Therefore, while var-Na inhibits LD loss by reducing membrane damage, unlike orlistat, it does not prevent lipolysis of the LA triglycerides by the invading lipase or consequent worsening of fat necrosis by NEFA as shown previously[32] (Figure 6H). On thin-layer chromatography, orlistat and var-Na partly reduced phospholipid hydrolysis by PPLE (Figure 6I). These findings again support the redundant roles of phospholipases and lipases in damaging cell membranes.

Recombinant pancreatic lipases along with phospholipase activity replicate fat necrosis *in vitro*

PNLIP contributes to 80%–90% of the pancreas's lipolytic activity.[71,72] Previous studies suggest that both PNLIP and PNLIPRP2 may access adipocyte LDs during fat necrosis.[32] We studied this in a stepwise manner, first using $HbPLA_2$ (which cannot hydrolyze triglyceride; Figure 4B), followed by PNLIP + CLPS (lipase), which causes negligible phospholipid hydrolysis (Figure 4A). We, therefore, treated 3T3-L1 cells with recombinant phospholipase $HbPLA_2$, followed by lipase. On western blotting (Figures 7A and S16), $HbPLA_2$ released ATGL, HSL, perilipin-1, and adiponectin from 3T3-L1 cells into the supernatant (Figure 7A), which was not

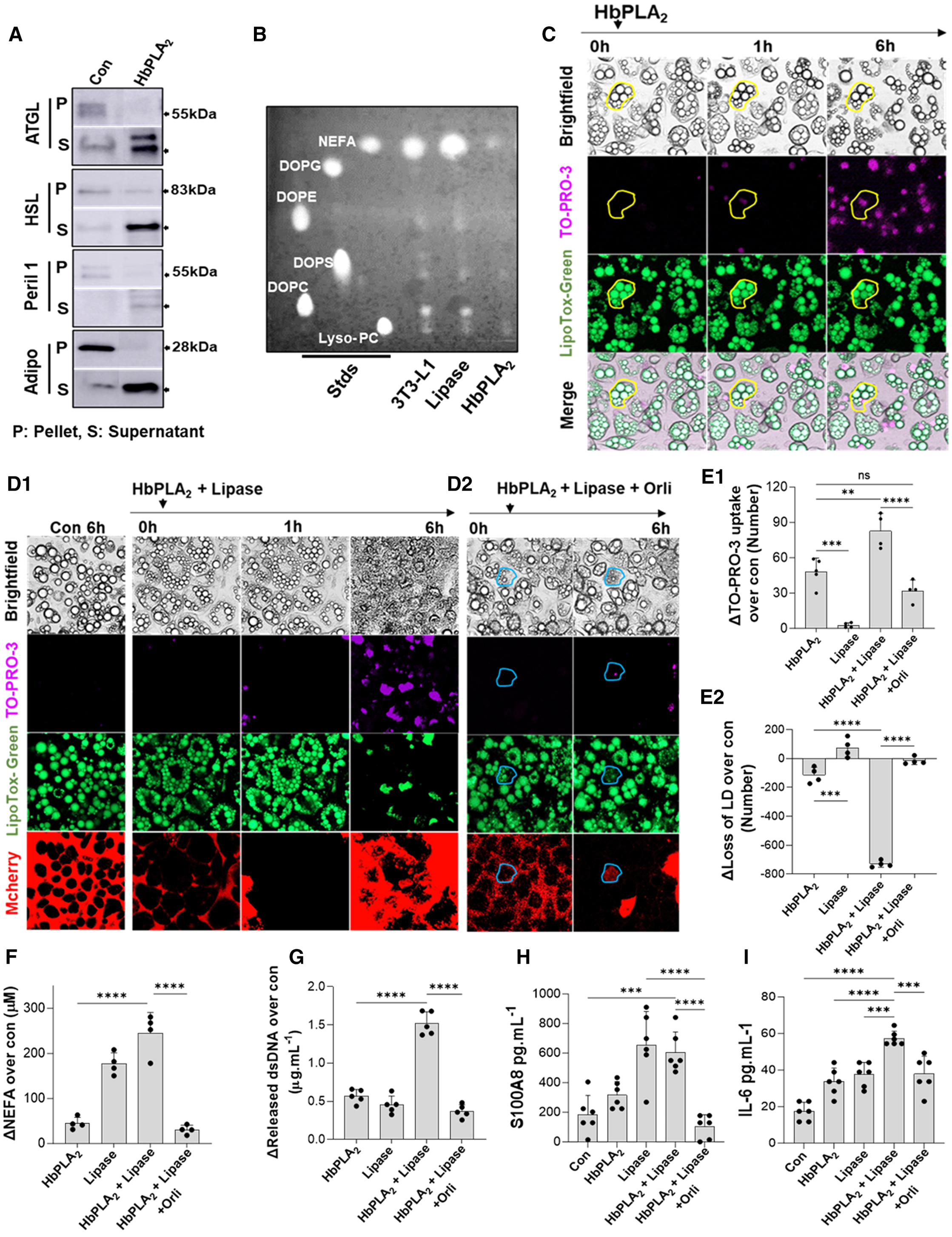

Figure 7. Recombinant pancreatic lipases along with phospholipase activity replicate fat necrosis *in vitro*

(A) Western blot images comparing detectable ATGL, HSL, perilipin-1 (Peri-1), and adiponectin (Adipo) bands in the 3T3-L1 pellet (P) and supernatant (S) treated by pure enzymes.

(B) TLC shows the breakdown of phospholipids from the 3T3-L1 cell membrane treated by lipase and $HbPLA_2$. Standards: NEFA (linoleic acid), DOPG, DOPE, DOPS, DOPC, and Lyso-PC.

(C) Live imaging of 3T3-L1 treated by $HbPLA_2$ alone, showing cell death (yellow outline), TO-PRO-3 uptake, and LD evolution.

(D1 and D2) The lipolysis process of 3T3-L1 LD-triglycerides was performed using lipase and $HbPLA_2$, and in the presence of orlistat (D2, blue ovals).

(legend continued on next page)

enhanced by lipase (data not shown). This loss of ATGL and HSL is similar to human and mouse fat necrosis (Figure 2L) and is consistent with PLA_2-mediated membrane damage causing loss of adipocyte proteins. This was verified on thin-layer chromatography (Figure 7B), which showed $HbPLA_2$ to hydrolyze all phospholipids, whereas lipase treatment appeared similar to controls. On live imaging, 3T3-L1 cells exposed to $HbPLA_2$ progressively lost membrane integrity starting at 1 h (indicated by yellow ovals, Figure 7C), noted as TO-PRO-3 uptake (Figures 7C, second row, and 7E1); however, LDs and lipolysis were minimally affected (Figures 7E and 7F; Videos S16 and S17, merged and TO-PRO-3 channels). Interestingly, cells exposed to $HbPLA_2$ and lipase developed rapidly progressive fat necrosis (Figure 7D1). At baseline, PNLIPmCherry bordered the 3T3-L1 cells, and LDs were discrete and intact. Fat necrosis, triggered by PNLIPmCherry entry into 3T3-L1 cells, led to loss of LDs (Figure 7D1; Videos S18, S19, S20, S21, and S22) and increased TO-PRO-3 uptake (Figures 7D1, second row, and 7E1), with loss of LDs (Figures 7D1, third row, and 7E2) and PNLIPmCherry spreading through the cytoplasm (Figure 7D, bottom row). Treating 3T3-L1 cells with the inactive $PNLIP^{S152G}$mCherry mutant in the presence of $HbPLA_2$ did not result in LD hydrolysis (yellow ovals, see Figure S17), despite TO-PRO-3 uptake and the $PNLIP^{S152G}$mCherry entry. Thus, active PNLIP is required for hydrolyzing triglycerides in LDs and mediating fat necrosis.

This loss of LDs and fat necrosis was associated with increased NEFA release due to hydrolysis of their triglycerides (Figure 7F). Fat necrosis also caused DAMP release, noted as an increase in dsDNA and S100A8, and also released IL-6 (Figures 7G–7I) along with increasing histone-DNA complexes and LDH leakage (data not shown). All these were prevented by orlistat. Live imaging showed that pretreatment with orlistat significantly reduced cell injury and loss of LDs (Figures 7D2, 7E1, and 7E2; Video S23). The rare cells that were TO-PRO-3 positive and took up the lipase still harbored intact LDs (blue ovals, Figure 7D2). Therefore, excessive LD lipolysis due to the entry of exogenous pancreatic lipase causes fat necrosis of adipocytes, which rapidly generates excess NEFA in a manner unregulated by endogenous adipocyte lipases.

DISCUSSION

Here, we note that pancreatic injury may worsen heart failure from excess fatty acids generated by pancreatic lipases during fat necrosis and that pancreatic lipase inhibition or genetic deletion prevents such an exacerbation. This is clinically relevant since a therapy that inhibits pancreatic lipases has entered clinical trials with a goal to prevent organ failure (ClinicalTrials.gov ID NCT06080789) and could be tested in clinical trials of heart failure.

In our study of the 4 million heart failure patients in the NIS database (Figure 1; Tables 1 and 2), those with unexplained AP had higher mortality despite a lower prevalence of detrimental risk factors compared to non-AP patients. Please note that a substantial proportion of heart failure patients with painless AP[12] may not have been diagnosed as AP, and thus, AP's role may have been underestimated in the database. Pancreatic injury during heart failure can result in the spillage of pancreatic enzymes into the surrounding visceral fat. The phospholipase activity of these enzymes can injure cell membranes and allow pancreatic lipases, principally PNLIP, to enter adipocytes. PNLIP, along with CLPS, can rapidly hydrolyze the LD and generate excessive NEFA in an unregulated fashion. Such excessive NEFA generation from lipolytic fat necrosis can then impair cardiomyocyte function, worsen cardiac injury, and exacerbate heart failure. Fat necrosis is also associated with the release of DAMPs from the injured cells, and an inflammatory response, along with organ failure. PNLIP alone is unlikely to cause these, since lipase increase in lean mice did not increase troponin-I levels or cause echocardiographic abnormalities (see Figure S9).

Adipocyte injury[40] or inflammation without evidence of pancreatic involvement is usually benign,[40,73,74] as seen in diverticulitis (Figure 2E), which, unlike pancreatitis, is rarely associated with organ failure. Our study explains the lipolytic mechanisms that result in fat necrosis by the closeness of the enzyme-rich pancreas to the several kilograms of visceral fat containing its substrate triglycerides. The dominantly exocrine pancreas' mass (40–200 g)[20] in proportion to the typical 3–5 kg of visceral fat[18,19] exceeds the typical enzyme-to-substrate ratios of $1{:}10^5$–10^6 even if individual enzymes formed 0.1% of pancreatic mass. This high enzyme-substrate ratio may underlie the explosive lipolytic release of NEFAs that worsen heart failure.[3,4]

Pancreatic enzymes, including PLA_2, CLPS, and lipases,[40,43] have previously been noted in fat necrosis during AP.[40,43] Previous studies show that Src,[25] actin-myosin,[75] and Munc-18c[76] contribute to basolateral leakage of these enzymes (which are normally secreted apically into the ductal system) during AP. Moreover, pancreatic ischemia during the disease[37,77,78] has been noted previously as painless lipase elevation with pancreatic injury.[12]

We note that fat pad injections of PPLE, which contains group 1B phospholipase, PNLIP, PNLIPRP2, amylase, and CLPS (Figures 3E and 3F), mimic the phenotype and fat necrosis due to doxorubicin-induced heart failure, IL12,18 pancreatitis[26,32] (Figures 3E and 3F), and cerulein pancreatitis shown previously.[32,79] The resulting PPLE-induced NEFA generation (Figures 3E, 3F, and 3H) caused cardiac injury and heart failure. Both PPLE injection and pancreatitis also elevated serum troponin-I, consistent with cardiac injury, and reduced carotid pulse distention, along with reducing survival. Please note that cardiac injury, heart failure, and hypotension progressed after PPLE injections (Figures 3J–3O) despite fluid supplementation being sufficient to cause hemodilution, reduce hemoglobin,

(E1–G) TO-PRO-3 uptake (E1), loss of LD (E2), released NEFA (F), and dsDNA leakage (G) from 3T3-L1 cells treated by different combinations of pure enzymes were determined.
(H and I) Released S100A8 (H) and IL-6 (I) were determined from the supernatant of 3T3-L1 cells treated by recombinant proteins.
$^{***}p < 0.001$ and $^{****}p < 0.0001$ indicate a significant difference between different groups ($n = 6$) using one-way ANOVA. Data are represented as mean/± SD.

and cause weight gain. This supports the idea that cardiac dysfunction had a bigger role than a vascular leak in the hypotension that developed. Additionally, this hemodilution and weight gain were similar in the orlistat group, who had normal troponin-I, echocardiographic parameters, carotid pulse distention, renal parameters, and outcomes (Figures 3J–3O). Therefore, pharmacologic inhibition of lipase prevented fat necrosis, NEFA generation, and the resulting cardiac injury and dysfunction. The genetic deletion of PNLIP did not affect doxorubicin-induced pancreatic injury (seen as a similar increase in serum amylase and pancreatic edema with doxorubicin; Figures 1A and 1B) but prevented fat necrosis and NEFA generation (Figures 1C–1F), along with cardiac injury, seen as normalization of serum CK-MB, carotid pulse distention, cardiac stroke volume, and improved survival (Figures 1H–1K). This is consistent with the reduction in cardiac injury markers and improvement in cardiac function by pharmacologic inhibition of PPLE-induced fat necrosis by orlistat (Figures 3J–3O and 3B–3D), and also genetic deletion of PNLIP normalizing troponin-I elevation and carotid pulse distention, in both IL12,18 and cerulein pancreatitis (Figures 3B–3D).

Interestingly, we note the absence of adipocyte lipases and perilipin-1 in both human (Figures 2E and 2L) and mouse fat necrosis (Figure 3G). Mechanistically, phospholipase activity causes adipocyte membrane damage and the release of adipocyte proteins, including ATGL, HSL, and perilipin-1, into the medium (Figure 7A). This is similar to PPLE (Figure 6A) and phospholipase (Figure 7A). In some cases, we note ATGL and HSL degradation, which may be due to the presence of trypsin in necrotic fat.[32] Whether pancreatic lipases also have proteolytic activity remains to be studied.

We note that the phospholipase activity of both PPLE and $HbPLA_2$ causes membrane damage in HeLa cells (without LDs; Figures 4A–4C, 4F, 4G, and 5C–5E) and 3T3-L1 cells (Figures 6B–6E, 6F, 6I, 7A–7C, and 7E1). This is via the ability of PLA_2 to hydrolyze membrane phospholipids such as PC and PG (Figures 4G, 5A, and S14A) and is verified by the intracellular entry of TO-PRO-3 (purple fluorescence in Figure 7C) and loss of adipocyte membranes (Figure 7C, TO-PRO-3 uptake and merged Videos S16 and S17) without loss of LDs. Phospholipase activity, therefore, allows the entry of the proteins PNLIPmCherry (78 kDa) and its cofactor CLPS (10 kDa) into adipocytes, which hydrolyze the triglyceride-rich LDs in 3T3-L1 cells, causing their disappearance (Figures 7D1 and 7E2), while generating NEFA and releasing DAMPs during fat necrosis. It is important to note that fat necrosis is, therefore, distinct from adipocyte death (Figures 7C and 7E1) or death of HeLa cells (Figures 4C, 4E, and 4F), both of which can be caused solely by the phospholipase activities contained in PPLE and $HbPLA_2$ (Figures 4A and 5A). However, this phospholipase activity, while allowing the entry of TO-PRO-3 into cells and showing loss of cell membranes, does not result in the loss of LDs, NEFA generation, or release of DAMPs like dsDNA or S100A8.

This is unlike the lipolytic activity of PNLIP and CLPS, which generates proinflammatory NEFA, cytokines, and DAMPs, resulting in organ failure and systemic inflammation.[26,32]

A clue to the redundant role of PNLIPRP2 in fat necrosis comes from orlistat significantly reducing the phospholipase activity of PPLE (Figure 5A) while not affecting $HbPLA_2$ (Figure 4A), which is a pure phospholipase. This may explain the partial protection from PPLE-induced membrane damage provided by varespladib (Figure 6C). At the same time, orlistat has a more pronounced effect (Figures 6D–6H), and this is consistent with PNLIPRP2 having dual phospholipase and lipase activity.[69,70] Our study is currently limited by not evaluating the role of PNLIPRP2 in lipolytic fat necrosis. Previous studies have established the role of PNLIP as the principal triglyceride lipase responsible for fat necrosis.[32] The exact phospholipase(s) and role of PNLIPRP2 in fat necrosis will need to be evaluated in future studies. Moreover, the potential redundancy of PNLIPRP2 and multiple pancreatic enzymes with PLA_2 activity[67,68] led to using a more potent (Figure 4A) but non-mammalian $HbPLA_2$ to study membrane damage, and also the use of pharmacologic inhibition with class-specific agents, since genetically deleting specific phospholipases is unlikely to achieve a protective phenotype. Our use of recombinant enzyme proteins, however, does support their roles in fat necrosis. While the use of doxorubicin for inducing heart failure is also a potential limiting factor, our data in mice agree with its published effects of causing acute cardiac dysfunction in humans[50] without preexisting heart failure and worsening with obesity.[55]

These studies also support that fat necrosis resulting from unregulated lipolysis (thus generating excessive NEFA) is mechanistically distinct from fat involvement without lipolysis, such as diverticulitis, fat infarction resulting from impaired blood supply (e.g., mesenteric infarction),[73] trauma,[80] or incidental involvement of fat in inflammation of adjacent viscera, such as in epiploic appendagitis,[74] that do not affect the disease course.

Our findings thus explain the worse clinical outcomes noted with pancreatic enzyme and NEFA elevation in heart failure and cardiac arrest and are consistent with autopsy evidence of pancreatitis reported in heart failure. Interventions to prevent such unregulated lipolysis and fat necrosis by pancreatic enzyme leak may thus be a novel approach to prevent the worsening of the clinical course of heart failure.

Limitations of the study

The clinical NIS data depend on diagnostic coding to diagnose heart failure and AP. While being broad and including over 4 million patients, this may have missed patients with painless lipase elevation and heart failure, thus underestimating the recently shown deleterious role of pancreatic lipase,[81] which hydrolyzes triglycerides and worsens organ failure.[81] While pharmacologic inhibition with orlistat was protective in the current study, it remains to be seen whether the lipase inhibitor RABI-767, which is currently in phase 2 clinical trials for preventing organ failure in pancreatitis (https://clinicaltrials.gov/study/NCT06080789), can prevent cardiac injury or worsening of heart failure. Additionally, we did not look for concurrent evidence of hypocalcemia or hypoalbuminemia, which results from excess fatty acids released from fat necrosis,[36,82] or study if replacing them provides additional protection.[83]

In animal studies, we measured the total fatty acids released from fat necrosis. This is a limitation, since several studies have shown unsaturated triglycerides to be more prone to lipolysis and unsaturated fatty acids to be more lipotoxic than

CellPress
OPEN ACCESS

saturated ones by virtue of their aqueous stability[79] and resulting amphipathic liponecrosis.[84] While using triglyceride or LA (Figure S9) and non-esterified LA in the cardiomyocyte studies partly addressed this, saturated fatty acids would need to be controlled for.

The doxorubicin model, while widely used,[45] was the only heart failure model studied. We did not find the non-invasive isoproterenol model to cause cardiac injury with isoproterenol alone, despite attempting both subcutaneous[85] and intraperitoneal routes.[86] Future studies in reproducible models of heart failure are needed, wherein the model itself induces clear cardiac injury and pancreatic enzyme elevation similar to humans,[1,2] along with mild reductions in left ventricular end-diastolic volume and stroke volume. Such a model would allow for the deleterious effects of fat necrosis to be tested more rigorously.

Lastly, basolateral leakage from pancreatic acini[25] may release numerous enzymes. Future studies are needed to compare their synergistic and/or redundant roles, e.g., how lipases and phospholipases interact to cause fat necrosis. We used $HbPLA_2$ in this initial study; however, the pancreas has at least 2 enzymes, i.e., PLA2G1B and PNLIPRP2 (which has dual lipase and phospholipase activity), that can damage adipocyte membranes. Moreover, PAF-AH (PLA2G7) is also active in pancreatitis, and secretory phospholipase PLA2G2A, which is present in inflammatory cells, can be released into fat during inflammatory conditions.[87,88] The roles of these enzymes will need to be determined in future studies.

RESOURCE AVAILABILITY

Lead contact

Further information and requests for resources and reagents should be directed to and will be fulfilled by the lead contact, Dr. Vijay P. Singh (singh.vijay@mayo.edu).

Material availability

This study did not generate any new materials or reagents.

Data and code availability

- All data reported in this paper will be shared by the lead contact upon request
- This paper does not report the original code
- Any additional information required to reanalyze the data reported in this paper is available from the lead contact upon request

ACKNOWLEDGMENTS

This project was supported by grant number FP00126724 from NPF (N.S.), RO1DK092460, R01DK119646, and R01AA031257 from the NIDDK, and PR191945 under W81XWH-20-1-0400 and PR240750 under W81XWH-16-1-0668 from the DOD (V.P.S.). Early stages of this project received funding from UL1 RR024153 from the National Center for Research Resources (NCRR), a component of the National Institutes of Health (NIH) and NIH Roadmap for Medical Research (V.P.S. and S.N.), and the Clinical Translational Science Institute supported by the NIH through grants UL1 RR024153 and UL1 TR000005. The contents of the manuscript are solely the responsibility of the authors and do not necessarily represent the official view of the NCRR or NIH. Information on the NCRR is available at http://www.ncrr.nih.gov/. Information on Re-engineering the Clinical Research Enterprise is available at http://nihroadmap.nih.gov/clinicalresearch/overview-translational.asp. We thank the histology core, including Jenny Pettengill, Stephen Lesueur, and Boyd Palmer, for the tissue processing and special stains (e.g., von Kossa). We also thank the Biospecimens Accessioning and Processing (BAP) core at the Mayo Clinic for the transport of human samples. We would like to thank Ms. Amanda Richards for her help with the acquisition of echocardiographic data in the initial stages of the study.

AUTHOR CONTRIBUTIONS

V.P.S. designed, supervised, and conceptualized the study. Acquisition of data was facilitated and carried out by N.S. (cell, biochemical, protein, and *in vivo* studies), B.K. (*in vivo* and echocardiographic studies), C.d.O. (*in vivo* pancreatitis studies), B.E.K. (NIS database), K.H.D. (NIS database), B.M. (lipidomic), M.S. (tunnel assays), S.K. (cardiomyocyte studies), S.N. (human tissue histology), K.P. (*in vivo* studies), and S.J. (clinical literature review). Analysis and interpretation of respective data were done by N.S., B.K., S.K., S.T. (live imaging), B.M., S.J., and V.P.S., who also helped in the critical evaluation of the manuscript. The manuscript was drafted by N.S. and V.P.S. Statistical analysis was done by N.S., B.E.K., K.H.D., and V.P.S.

DECLARATION OF INTERESTS

V.P.S. is the inventor of RABI-767, for which he holds multiple patents. He received grant funding and royalties and is a shareholder of Arrivo BioVentures.

STAR★METHODS

Detailed methods are provided in the online version of this paper and include the following:

- KEY RESOURCES TABLE
- EXPERIMENTAL MODEL AND STUDY PARTICIPANT DETAILS
  - Animal studies
  - *In vitro* studies
  - Cell lines
- METHOD DETAILS
  - Nationwide inpatient sample (NIS) data source studies
  - Study population
  - Definitions Of variables
  - Statistical analyses
  - CT imaging analysis
  - Doxorubicin (Doxo) induced heart failure
  - Acute pancreatitis models
  - CK-MB and troponin I assay
  - Echocardiography
  - Histology and immunohistochemical studies
  - Injury parameters
  - Terminal deoxynucleotidyl transferase dUTP nick end labeling (TUNEL)
  - Cell lines culture and use
  - Confocal microscopy
  - Calcium oscillation and contractions of cardiomyocytes
  - Cell injury markers and LD loss
  - Luminex assays
  - Western blot analysis
  - Lipid extraction and thin-layer chromatography
  - Materials
  - Lipids
  - Proteins
  - Enzymatic activities
  - Pharmacological inhibition
- QUANTIFICATION AND STATISTICAL ANALYSIS

SUPPLEMENTAL INFORMATION

Supplemental information can be found online at https://doi.org/10.1016/j.xcrm.2025.102147.

Received: March 25, 2024
Revised: January 22, 2025
Accepted: April 30, 2025
Published: June 2, 2025

REFERENCES

1. Courelli, V., Ahmad, A., Ghassemian, M., Pruitt, C., Mills, P.J., and Schmid-Schönbein, G.W. (2021). Digestive Enzyme Activity and Protein Degradation in Plasma of Heart Failure Patients. Cell. Mol. Bioeng. *14*, 583–596. https://doi.org/10.1007/s12195-021-00693-w.
2. Parissis, J.T., Adamopoulos, S.N., Venetsanou, K.F., Karas, S.M., and Kremastinos, D.T. (2003). Elevated plasma amylase levels in advanced chronic heart failure secondary to ischemic or idiopathic dilated cardiomyopathy: correlation with circulating interleukin-6 activity. J. Interferon Cytokine Res. *23*, 329–333. https://doi.org/10.1089/107999003766628179.
3. Degoricija, V., Trbušić, M., Potočnjak, I., Radulović, B., Pregartner, G., Berghold, A., Scharnagl, H., Stojakovic, T., Tiran, B., and Frank, S. (2019). Serum concentrations of free fatty acids are associated with 3-month mortality in acute heart failure patients. Clin. Chem. Lab. Med. *57*, 1799–1804. https://doi.org/10.1515/cclm-2019-0037.
4. Yu, Y., Jin, C., Zhao, C., Zhu, S., Meng, S., Ma, H., Wang, J., and Xiang, M. (2021). Serum Free Fatty Acids Independently Predict Adverse Outcomes in Acute Heart Failure Patients. Front. Cardiovasc. Med. *8*, 761537. https://doi.org/10.3389/fcvm.2021.761537.
5. Park, S.Y., Kim, M.J., Park, I., Kim, H.Y., Lee, M., Park, Y.S., and Chung, S.P. (2022). Predisposing Factors and Neurologic Outcomes of Patients with Elevated Serum Amylase and/or Lipase after Out-of-Hospital Cardiac Arrest: A Retrospective Cohort Study. J. Clin. Med. *11*, 1426. https://doi.org/10.3390/jcm11051426.
6. Czapari, D., Varadi, A., Farkas, N., Nyari, G., Marta, K., Vancsa, S., Nagy, R., Teutsch, B., Bunduc, S., Eross, B., et al. (2023). Detailed Characteristics of Post-discharge Mortality in Acute Pancreatitis. Gastroenterology *165*, 682–695. https://doi.org/10.1053/j.gastro.2023.05.028.
7. Warshaw, A.L., and O'Hara, P.J. (1978). Susceptibility of the pancreas to ischemic injury in shock. Ann. Surg. *188*, 197–201. https://doi.org/10.1097/00000658-197808000-00012.
8. Gullo, L., Cavicchi, L., Tomassetti, P., Spagnolo, C., Freyrie, A., and D'Addato, M. (1996). Effects of ischemia on the human pancreas. Gastroenterology *111*, 1033–1038. https://doi.org/10.1016/s0016-5085(96)70072-0.
9. Haas, G.S., Warshaw, A.L., Daggett, W.M., and Aretz, H.T. (1985). Acute pancreatitis after cardiopulmonary bypass. Am. J. Surg. *149*, 508–515. https://doi.org/10.1016/s0002-9610(85)80048-9.
10. Kloppel G, D.T. (1984). Pathomorphology of Acute Pancreatitis. Analysis of 367 Autopsy Cases and 3 Surgical Specimens. Amsterdam, the Netherlands, New York, NY, Oxford.
11. Nordback, I., and Lauslahti, K. (1986). Clinical pathology of acute necrotising pancreatitis. J. Clin. Pathol. *39*, 68–74. https://doi.org/10.1136/jcp.39.1.68.
12. Chaffin, H., Trivedi, S., and Singh, V.P. (2022). Impact of abdominal imaging on the diagnosis of acute pancreatitis in patients with painless lipase elevation. Pancreatology *22*, 547–552. https://doi.org/10.1016/j.pan.2022.04.013.
13. Manjuck, J., Zein, J., Carpati, C., and Astiz, M. (2005). Clinical significance of increased lipase levels on admission to the ICU. Chest *127*, 246–250.
14. Oguntade, A.S., Islam, N., Malouf, R., Taylor, H., Jin, D., Lewington, S., and Lacey, B. (2023). Body Composition and Risk of Incident Heart Failure in 1 Million Adults: A Systematic Review and Dose-Response Meta-Analysis of Prospective Cohort Studies. J. Am. Heart Assoc. *12*, e029062. https://doi.org/10.1161/JAHA.122.029062.
15. Seki, Y., Obokata, M., Harada, T., Kagami, K., Sorimachi, H., Saito, Y., Kato, T., and Ishii, H. (2023). Adiposity and clinical outcomes in East Asian patients with heart failure and preserved ejection fraction. Int J Cardiol Heart Vasc *44*, 101162. https://doi.org/10.1016/j.ijcha.2022.101162.
16. Jamaly, S., Carlsson, L., Peltonen, M., Andersson-Assarsson, J.C., and Karason, K. (2021). Heart failure development in obesity: underlying risk factors and mechanistic pathways. ESC Heart Fail *8*, 356–367. https://doi.org/10.1002/ehf2.13081.
17. Tan, R., Ng, Z.Q., Misur, P., and Wijesuriya, R. (2023). Relationship of computed tomography quantified visceral adiposity with the severity and complications of acute pancreatitis: a systematic review. Jpn. J. Radiol. *41*, 1104–1116. https://doi.org/10.1007/s11604-023-01430-1.
18. Kobayashi, J., Tadokoro, N., Watanabe, M., and Shinomiya, M. (2002). A novel method of measuring intra-abdominal fat volume using helical computed tomography. Int. J. Obes. Relat. Metab. Disord. *26*, 398–402. https://doi.org/10.1038/sj.ijo.0801921.
19. Choh, A.C., Demerath, E.W., Lee, M., Williams, K.D., Towne, B., Siervogel, R.M., Cole, S.A., and Czerwinski, S.A. (2009). Genetic analysis of self-reported physical activity and adiposity: the Southwest Ohio Family Study. Public Health Nutr. *12*, 1052–1060. https://doi.org/10.1017/s1368980008003583.
20. Innes, J.T., and Carey, L.C. (1994). Normal pancreatic dimensions in the adult human. Am. J. Surg. *167*, 261–263. https://doi.org/10.1016/0002-9610(94)90088-4.
21. Rosso, E., Casnedi, S., Pessaux, P., Oussoultzoglou, E., Panaro, F., Mahfud, M., Jaeck, D., and Bachellier, P. (2009). The role of "fatty pancreas" and of BMI in the occurrence of pancreatic fistula after pancreaticoduodenectomy. J. Gastrointest. Surg. *13*, 1845–1851.
22. Garaulet, M., Hernandez-Morante, J.J., Lujan, J., Tebar, F.J., and Zamora, S. (2006). Relationship between fat cell size and number and fatty acid composition in adipose tissue from different fat depots in overweight/obese humans. Int. J. Obes. *30*, 899–905. https://doi.org/10.1038/sj.ijo.0803219.
23. Pandiri, A.R. (2014). Overview of exocrine pancreatic pathobiology. Toxicol. Pathol. *42*, 207–216. https://doi.org/10.1177/0192623313509907.
24. Aoki, J., Inoue, A., Makide, K., Saiki, N., and Arai, H. (2007). Structure and function of extracellular phospholipase A1 belonging to the pancreatic lipase gene family. Biochimie *89*, 197–204. https://doi.org/10.1016/j.biochi.2006.09.021.
25. Singh, V.P., and McNiven, M.A. (2008). Src-mediated cortactin phosphorylation regulates actin localization and injurious blebbing in acinar cells. Mol. Biol. Cell *19*, 2339–2347.
26. Navina, S., Acharya, C., DeLany, J.P., Orlichenko, L.S., Baty, C.J., Shiva, S.S., Durgampudi, C., Karlsson, J.M., Lee, K., Bae, K.T., et al. (2011). Lipotoxicity causes multisystem organ failure and exacerbates acute pancreatitis in obesity. Sci. Transl. Med. *3*, 107ra110. https://doi.org/10.1126/scitranslmed.3002573.
27. Acharya, C., Cline, R.A., Jaligama, D., Noel, P., Delany, J.P., Bae, K., Furlan, A., Baty, C.J., Karlsson, J.M., Rosario, B.L., et al. (2013). Fibrosis Reduces Severity of Acute-on-Chronic Pancreatitis in Humans. Gastroenterology *145*, 466–475. https://doi.org/10.1053/j.gastro.2013.05.012.
28. Monson, E.A., Crosse, K.M., Duan, M., Chen, W., O'Shea, R.D., Wakim, L.M., Carr, J.M., Whelan, D.R., and Helbig, K.J. (2021). Intracellular lipid droplet accumulation occurs early following viral infection and is required for an efficient interferon response. Nat. Commun. *12*, 4303. https://doi.org/10.1038/s41467-021-24632-5.
29. Yang, A., and Mottillo, E.P. (2020). Adipocyte lipolysis: from molecular mechanisms of regulation to disease and therapeutics. Biochem. J. *477*, 985–1008. https://doi.org/10.1042/bcj20190468.
30. Fernández-del Castillo, C., Harringer, W., Warshaw, A.L., Vlahakes, G.J., Koski, G., Zaslavsky, A.M., and Rattner, D.W. (1991). Risk factors for pancreatic cellular injury after cardiopulmonary bypass. N. Engl. J. Med. *325*, 382–387. https://doi.org/10.1056/nejm199108083250602.
31. Noel, P., Patel, K., Durgampudi, C., Trivedi, R.N., de Oliveira, C., Crowell, M.D., Pannala, R., Lee, K., Brand, R., Chennat, J., et al. (2016).

Peripancreatic fat necrosis worsens acute pancreatitis independent of pancreatic necrosis via unsaturated fatty acids increased in human pancreatic necrosis collections. Gut *65*, 100–111. https://doi.org/10.1136/gutjnl-2014-308043.

32. de Oliveira, C., Khatua, B., Noel, P., Kostenko, S., Bag, A., Balakrishnan, B., Patel, K.S., Guerra, A.A., Martinez, M.N., Trivedi, S., et al. (2020). Pancreatic triglyceride lipase mediates lipotoxic systemic inflammation. J. Clin. Investig. *130*, 1931–1947. https://doi.org/10.1172/jci132767.
33. Wolfe, R.R., Herndon, D.N., Jahoor, F., Miyoshi, H., and Wolfe, M. (1987). Effect of severe burn injury on substrate cycling by glucose and fatty acids. N. Engl. J. Med. *317*, 403–408. https://doi.org/10.1056/nejm198708133170702.
34. Sztefko, K., and Panek, J. (2001). Serum free fatty acid concentration in patients with acute pancreatitis. Pancreatology *1*, 230–236.
35. Kostenko, S., Khatua, B., Trivedi, S., Pillai, A.N., McFayden, B., Morsy, M., Rajalingamgari, P., Sharma, V., Noel, P., Patel, K., et al. (2023). Amphipathic liponecrosis impairs bacterial clearance and causes infection during sterile inflammation. Gastroenterology *165*, 999–1015. https://doi.org/10.1053/j.gastro.2023.05.034.
36. Cartin-Ceba, R., Khatua, B., El-Kurdi, B., Trivedi, S., Kostenko, S., Imam, Z., Smith, R., Snozek, C., Navina, S., Sharma, V., et al. (2022). Evidence Showing Lipotoxicity Worsens Outcomes in Covid-19 Patients and Insights About the Underlying Mechanisms. iScience *25*, 104322. https://doi.org/10.1016/j.isci.2022.104322.
37. Krejci, V., Hiltebrand, L., Banic, A., Erni, D., Wheatley, A.M., and Sigurdsson, G.H. (2000). Continuous measurements of microcirculatory blood flow in gastrointestinal organs during acute haemorrhage. Br. J. Anaesth. *84*, 468–475.
38. Bakker, O.J., van Santvoort, H., Besselink, M.G., Boermeester, M.A., van Eijck, C., Dejong, K., van Goor, H., Hofker, S., Ahmed Ali, U., Gooszen, H.G., and Bollen, T.L. (2013). Extrapancreatic necrosis without pancreatic parenchymal necrosis: a separate entity in necrotising pancreatitis? Gut *62*, 1475–1480. https://doi.org/10.1136/gutjnl-2012-302870.
39. Spanier, B.W., Nio, Y., van der Hulst, R.W., Tuynman, H.A., Dijkgraaf, M.G., and Bruno, M.J. (2010). Practice and yield of early CT scan in acute pancreatitis: a Dutch Observational Multicenter Study. Pancreatology *10*, 222–228. https://doi.org/10.1159/000243731.
40. Aho, H.J., Sternby, B., and Nevalainen, T.J. (1986). Fat necrosis in human acute pancreatitis. An immunohistological study. Acta Pathol Microbiol Immunol Scand A *94*, 101–105. https://doi.org/10.1111/j.1699-0463.1986.tb02970.x.
41. De Caro, J., Sias, B., Grandval, P., Ferrato, F., Halimi, H., Carriere, F., and De Caro, A. (2004). Characterization of pancreatic lipase-related protein 2 isolated from human pancreatic juice. Biochim. Biophys. Acta *1701*, 89–99. https://doi.org/10.1016/j.bbapap.2004.06.005.
42. Lowe, M.E. (2002). The triglyceride lipases of the pancreas. Journal of lipid research *43*, 2007–2016.
43. Aho, H.J., Sternby, B., Kallajoki, M., and Nevalainen, T.J. (1989). Carboxyl ester lipase in human tissues and in acute pancreatitis. Int. J. Pancreatol. *5*, 123–134.
44. Khatua, B., Trivedi, R.N., Noel, P., Patel, K., Singh, R., de Oliveira, C., Trivedi, S., Mishra, V., Lowe, M., and Singh, V.P. (2019). Carboxyl Ester Lipase May Not Mediate Lipotoxic Injury during Severe Acute Pancreatitis. Am. J. Pathol. *189*, 1226–1240. https://doi.org/10.1016/j.ajpath.2019.02.015.
45. Roca-Alonso, L., Pellegrino, L., Castellano, L., and Stebbing, J. (2012). Breast cancer treatment and adverse cardiac events: what are the molecular mechanisms? Cardiology *122*, 253–259. https://doi.org/10.1159/000339858.
46. Gilladoga, A.C., Manuel, C., Tan, C.T., Wollner, N., Sternberg, S.S., and Murphy, M.L. (1976). The cardiotoxicity of adriamycin and daunomycin in children. Cancer *37*, 1070–1078. https://doi.org/10.1002/1097-0142(197602)37:2+<1070::aid-cncr2820370814>3.0.co;2-6.
47. Lipshultz, S.E., Colan, S.D., Gelber, R.D., Perez-Atayde, A.R., Sallan, S.E., and Sanders, S.P. (1991). Late cardiac effects of doxorubicin therapy for acute lymphoblastic leukemia in childhood. N. Engl. J. Med. *324*, 808–815. https://doi.org/10.1056/nejm199103213241205.
48. Lipshultz, S.E., Lipsitz, S.R., Sallan, S.E., Dalton, V.M., Mone, S.M., Gelber, R.D., and Colan, S.D. (2005). Chronic progressive cardiac dysfunction years after doxorubicin therapy for childhood acute lymphoblastic leukemia. J. Clin. Oncol. *23*, 2629–2636. https://doi.org/10.1200/jco.2005.12.121.
49. Praga, C., Beretta, G., Vigo, P.L., Lenaz, G.R., Pollini, C., Bonadonna, G., Canetta, R., Castellani, R., Villa, E., Gallagher, C.G., et al. (1979). Adriamycin cardiotoxicity: a survey of 1273 patients. Cancer Treat Rep. *63*, 827–834.
50. Steinherz, L.J., Steinherz, P.G., Tan, C.T., Heller, G., and Murphy, M.L. (1991). Cardiac toxicity 4 to 20 years after completing anthracycline therapy. JAMA *266*, 1672–1677.
51. Swain, S.M., Whaley, F.S., and Ewer, M.S. (2003). Congestive heart failure in patients treated with doxorubicin: a retrospective analysis of three trials. Cancer *97*, 2869–2879. https://doi.org/10.1002/cncr.11407.
52. van Dalen, E.C., van der Pal, H.J., Kok, W.E., Caron, H.N., and Kremer, L.C. (2006). Clinical heart failure in a cohort of children treated with anthracyclines: a long-term follow-up study. Eur. J. Cancer *42*, 3191–3198. https://doi.org/10.1016/j.ejca.2006.08.005.
53. Von Hoff, D.D., Layard, M.W., Basa, P., Davis, H.L., Jr., Von Hoff, A.L., Rozencweig, M., and Muggia, F.M. (1979). Risk factors for doxorubicin-induced congestive heart failure. Ann. Intern. Med. *91*, 710–717. https://doi.org/10.7326/0003-4819-91-5-710.
54. Chitadze, T., Sharashidze, N., Rukhadze, T., Lomia, N., and Saatashvili, G. (2024). Evaluation of left ventricular systolic function in postmenopausal women with breast cancer receiving adjuvant anthracycline and trastuzumab therapy: a 2-year follow-up study. Georgian Med. News, 284–293.
55. Guenancia, C., Lefebvre, A., Cardinale, D., Yu, A.F., Ladoire, S., Ghiringhelli, F., Zeller, M., Rochette, L., Cottin, Y., and Vergely, C. (2016). Obesity As a Risk Factor for Anthracyclines and Trastuzumab Cardiotoxicity in Breast Cancer: A Systematic Review and Meta-Analysis. J. Clin. Oncol. *34*, 3157–3165. https://doi.org/10.1200/jco.2016.67.4846.
56. Banks, P.A., Bollen, T.L., Dervenis, C., Gooszen, H.G., Johnson, C.D., Sarr, M.G., Tsiotos, G.G., and Vege, S.S. (2013). Classification of acute pancreatitis–2012: revision of the Atlanta classification and definitions by international consensus. Gut *62*, 102–111. https://doi.org/10.1136/gutjnl-2012-302779.
57. Liu, X., Wang, X., Zhang, X., Xie, Y., Chen, R., and Chen, H. (2012). C57BL/6 mice are more appropriate than BALB/C mice in inducing dilated cardiomyopathy with short-term doxorubicin treatment. Acta Cardiol. Sin. *28*, 236–240.
58. Yalçin, E., Oruç, E., Cavuşoğlu, K., and Yapar, K. (2010). Protective role of grape seed extract against doxorubicin-induced cardiotoxicity and genotoxicity in albino mice. J. Med. Food *13*, 917–925. https://doi.org/10.1089/jmf.2009.0162.
59. Krishnamurthy, B., Rani, N., Bharti, S., Golechha, M., Bhatia, J., Nag, T.C., Ray, R., Arava, S., and Arya, D.S. (2015). Febuxostat ameliorates doxorubicin-induced cardiotoxicity in rats. Chem. Biol. Interact. *237*, 96–103. https://doi.org/10.1016/j.cbi.2015.05.013.
60. Guo, M., Fan, X., Tuerhongjiang, G., Wang, C., Wu, H., Lou, B., Wu, Y., Yuan, Z., and She, J. (2021). Targeted metabolomic analysis of plasma fatty acids in acute myocardial infarction in young adults. Nutr Metab Cardiovasc Dis *31*, 3131–3141. https://doi.org/10.1016/j.numecd.2021.06.024.
61. Kloppel, G., Dreyer, T., Willemer, S., Kern, H.F., and Adler, G. (1986). Human acute pancreatitis: its pathogenesis in the light of immunocytochemical and ultrastructural findings in acinar cells. Virchows Arch. *409*, 791–803.

62. Nordback, I., and Lauslahti, K. (1986). Clinical pathology of acute necrotising pancreatitis. J. Clin. Pathol. *39*, 68–74.

63. Kloppel G, v.G.R., Dreyer T. (1984). Pathomorphology of acute pancreatitis. Analysis of 367 Autopsy Cases and 3 Surgical Specimens.

64. Kloppel, G., and Maillet, B. (1991). Pseudocysts in chronic pancreatitis: a morphological analysis of 57 resection specimens and 9 autopsy pancreata. Pancreas *6*, 266–274.

65. Klöppel, G. (1995). Pathomorphology of acute pancreatitis. Ann. Ital. Chir. *66*, 149–154.

66. Xiao, X., Ross, L.E., Miller, R.A., and Lowe, M.E. (2011). Kinetic properties of mouse pancreatic lipase-related protein-2 suggest the mouse may not model human fat digestion. Journal of lipid research *52*, 982–990. https://doi.org/10.1194/jlr.M014290.

67. Dennis, E.A. (1994). Diversity of group types, regulation, and function of phospholipase A2. J. Biol. Chem. *269*, 13057–13060.

68. Nagai, H., Henrich, H., Wünsch, P.-H., Fischbach, W., and Mössner, J. (1989). Role of pancreatic enzymes and their substrates in autodigestion of the pancreas: In vitro studies with isolated rat pancreatic acini. Gastroenterology *96*, 838–847. https://doi.org/10.1016/0016-5085(89)90910-4.

69. Jennens, M.L., and Lowe, M.E. (1995). Rat GP-3 is a pancreatic lipase with kinetic properties that differ from colipase-dependent pancreatic lipase. Journal of lipid research *36*, 2374–2382.

70. Withers-Martinez, C., Carriere, F., Verger, R., Bourgeois, D., and Cambillau, C. (1996). A pancreatic lipase with a phospholipase A1 activity: crystal structure of a chimeric pancreatic lipase-related protein 2 from guinea pig. Structure *4*, 1363–1374. https://doi.org/10.1016/s0969-2126(96)00143-8.

71. De Caro, J., Sias, B., Grandval, P., Ferrato, F., Halimi, H., Carrière, F., and De Caro, A. (2004). Characterization of pancreatic lipase-related protein 2 isolated from human pancreatic juice. Biochim. Biophys. Acta *1701*, 89–99. https://doi.org/10.1016/j.bbapap.2004.06.005.

72. Thompson, W., Oslund, R.C., Bollinger, J., Ewing, H., and Gelb, M.H. (2012). High-throughput assay of secreted phospholipases A_2 inhibitors. Methods Mol. Biol. *861*, 149–158. https://doi.org/10.1007/978-1-61779-600-5_10.

73. Tonerini, M., Calcagni, F., Lorenzi, S., Scalise, P., Grigolini, A., and Bemi, P. (2015). Omental infarction and its mimics: imaging features of acute abdominal conditions presenting with fat stranding greater than the degree of bowel wall thickening. Emerg. Radiol. *22*, 431–436. https://doi.org/10.1007/s10140-015-1302-0.

74. Pereira, J.M., Sirlin, C.B., Pinto, P.S., Jeffrey, R.B., Stella, D.L., and Casola, G. (2004). Disproportionate fat stranding: a helpful CT sign in patients with acute abdominal pain. Radiographics *24*, 703–715. https://doi.org/10.1148/rg.243035084.

75. Torgerson, R.R., and McNiven, M.A. (1998). The actin-myosin cytoskeleton mediates reversible agonist-induced membrane blebbing. J. Cell Sci. *111*, 2911–2922.

76. Gaisano, H.Y., Lutz, M.P., Leser, J., Sheu, L., Lynch, G., Tang, L., Tamori, Y., Trimble, W.S., and Salapatek, A.M. (2001). Supramaximal cholecystokinin displaces Munc18c from the pancreatic acinar basal surface, redirecting apical exocytosis to the basal membrane. J. Clin. Investig. *108*, 1597–1611.

77. Fish, R.E., Lang, C.H., and Spitzer, J.A. (1986). Regional blood flow during continuous low-dose endotoxin infusion. Circ. Shock *18*, 267–275.

78. Hiltebrand, L.B., Krejci, V., Banic, A., Erni, D., Wheatley, A.M., and Sigurdsson, G.H. (2000). Dynamic study of the distribution of microcirculatory blood flow in multiple splanchnic organs in septic shock. Crit. Care Med. *28*, 3233–3241.

79. Khatua, B., El-Kurdi, B., Patel, K., Rood, C., Noel, P., Crowell, M., Yaron, J. R., Kostenko, S., Guerra, A., Faigel, D.O., et al. (2021). Adipose saturation reduces lipotoxic systemic inflammation and explains the obesity paradox. Sci. Adv. *7*, eabd6449. https://doi.org/10.1126/sciadv.abd6449.

80. PARSONS, W.B., Jr. (1924). TRAUMATIC FAT NECROSIS. Journal of the American Medical Association *83*, 1756–1757. https://doi.org/10.1001/jama.1924.02660220032010.

81. Rajalingamgari, P., Khatua, B., Summers, M.J., Kostenko, S., Chang, Y. H., Elmallahy, M., Anand, A., Narayana Pillai, A., Morsy, M., Trivedi, S., et al. (2024). Prospective observational study and mechanistic evidence showing lipolysis of circulating triglycerides worsens hypertriglyceridemic acute pancreatitis. J. Clin. Investig. *135*, e184785. https://doi.org/10.1172/jci184785.

82. Singh, V.P., Khatua, B., El-Kurdi, B., and Rood, C. (2020). Mechanistic basis and therapeutic relevance of hypocalcemia during severe COVID-19 infection. Endocrine *70*, 461–462. https://doi.org/10.1007/s12020-020-02530-y.

83. Khatua, B., Yaron, J.R., El-Kurdi, B., Kostenko, S., Papachristou, G.I., and Singh, V.P. (2020). Ringer's Lactate Prevents Early Organ Failure by Providing Extracellular Calcium. J. Clin. Med. *9*, 263. https://doi.org/10.3390/jcm9010263.

84. Kostenko, S., Khatua, B., Trivedi, S., Pillai, A.N., McFayden, B., Morsy, M., Rajalingamgari, P., Sharma, V., Noel, P., Patel, K., et al. (2023). Amphipathic Liponecrosis Impairs Bacterial Clearance and Causes Infection During Sterile Inflammation. Gastroenterology *165*, 999–1015.

85. Chen, Y., Guo, X., Zeng, Y., Mo, X., Hong, S., He, H., Li, J., Steinmetz, R., and Liu, Q. (2023). Ferroptosis contributes to catecholamine-induced cardiotoxicity and pathological remodeling. Free Radic. Biol. Med. *207*, 227–238. https://doi.org/10.1016/j.freeradbiomed.2023.07.025.

86. Forte, E., Panahi, M., Baxan, N., Ng, F.S., Boyle, J.J., Branca, J., Bedard, O., Hasham, M.G., Benson, L., Harding, S.E., et al. (2021). Type 2 MI induced by a single high dose of isoproterenol in C57BL/6J mice triggers a persistent adaptive immune response against the heart. J. Cell Mol. Med. *25*, 229–243. https://doi.org/10.1111/jcmm.15937.

87. Murakami, M., Yamamoto, K., Miki, Y., Murase, R., Sato, H., and Taketomi, Y. (2016). The Roles of the Secreted Phospholipase A(2) Gene Family in Immunology. Adv. Immunol. *132*, 91–134. https://doi.org/10.1016/bs.ai.2016.05.001.

88. Kuefner, M.S., Stephenson, E., Savikj, M., Smallwood, H.S., Dong, Q., Payré, C., Lambeau, G., and Park, E.A. (2021). Group IIA secreted phospholipase A2 (PLA2G2A) augments adipose tissue thermogenesis. Faseb j *35*, e21881. https://doi.org/10.1096/fj.202002481RR.

89. Huggins, K.W., Camarota, L.M., Howles, P.N., and Hui, D.Y. (2003). Pancreatic Triglyceride Lipase Deficiency Minimally Affects Dietary Fat Absorption but Dramatically Decreases Dietary Cholesterol Absorption in Mice. J. Biol. Chem. *278*, 42899–42905. https://doi.org/10.1074/jbc.M303422200.

90. Weidner, M., Taupp, M., and Hallam, S.J. (2010). Expression of recombinant proteins in the methylotrophic yeast Pichia pastoris. J. Vis. Exp. *36*, 1862. https://doi.org/10.3791/1862.

91. Gukovskaya, A.S., Gukovsky, I., Zaninovic, V., Song, M., Sandoval, D., Gukovsky, S., and Pandol, S.J. (1997). Pancreatic acinar cells produce, release, and respond to tumor necrosis factor-alpha. Role in regulating cell death and pancreatitis. J. Clin. Investig. *100*, 1853–1862. https://doi.org/10.1172/jci119714.

92. Quan, H., Li, B., Couris, C.M., Fushimi, K., Graham, P., Hider, P., Januel, J.M., and Sundararajan, V. (2011). Updating and validating the Charlson comorbidity index and score for risk adjustment in hospital discharge abstracts using data from 6 countries. Am. J. Epidemiol. *173*, 676–682. https://doi.org/10.1093/aje/kwq433kwq433.

93. Cao, Y., Ruan, Y., Shen, T., Huang, X., Li, M., Yu, W., Zhu, Y., Man, Y., Wang, S., and Li, J. (2014). Astragalus polysaccharide suppresses doxorubicin-induced cardiotoxicity by regulating the PI3k/Akt and p38MAPK pathways. Oxid. Med. Cell. Longev. *2014*, 674219. https://doi.org/10.1155/2014/674219.

94. Pei, X.M., Tam, B.T., Sin, T.K., Wang, F.F., Yung, B.Y., Chan, L.W., Wong, C. S., Ying, M., Lai, C.W., and Siu, P.M. (2016). S100A8 and S100A9 Are

CellPress
OPEN ACCESS

Associated with Doxorubicin-Induced Cardiotoxicity in the Heart of Diabetic Mice. Front. Physiol. *7*, 334. https://doi.org/10.3389/fphys.2016.00334.

95. Hermansen, K., and Wassermann, K. (1986). The effect of vitamin E and selenium on doxorubicin (Adriamycin) induced delayed toxicity in mice. Acta Pharmacol. Toxicol. *58*, 31–37.

96. Argun, M., Uzum, K., Sonmez, M.F., Ozyurt, A., Derya, K., Cilenk, K.T., Unalmis, S., Pamukcu, O., Baykan, A., Narin, F., et al. (2016). Cardioprotective effect of metformin against doxorubicin cardiotoxicity in rats. Anatol. J. Cardiol. *16*, 234–241. https://doi.org/10.5152/akd.2015.6185.

97. Spivak, M., Bubnov, R., Yemets, I., Lazarenko, L., Timoshok, N., Vorobieva, A., Mohnatyy, S., Ulberg, Z., Reznichenko, L., Grusina, T., et al. (2013). Doxorubicin dose for congestive heart failure modeling and the use of general ultrasound equipment for evaluation in rats. Longitudinal in vivo study. Med Ultrason *15*, 23–28.

98. Ding, S.P., Li, J.C., and Jin, C. (2003). A mouse model of severe acute pancreatitis induced with caerulein and lipopolysaccharide. World J. Gastroenterol. *9*, 584–589. https://doi.org/10.3748/wjg.v9.i3.584.

99. Patel, K., Trivedi, R.N., Durgampudi, C., Noel, P., Cline, R.A., DeLany, J. P., Navina, S., and Singh, V.P. (2015). Lipolysis of visceral adipocyte triglyceride by pancreatic lipases converts mild acute pancreatitis to severe pancreatitis independent of necrosis and inflammation. Am. J. Pathol. *185*, 808–819. https://doi.org/10.1016/j.ajpath.2014.11.019.

100. Grune, T., Ott, C., Häseli, S., Höhn, A., and Jung, T. (2019). The "MYOCYTER" – Convert cellular and cardiac contractions into numbers with ImageJ. Sci. Rep. *9*, 15112. https://doi.org/10.1038/s41598-019-51676-x.

101. Schindelin, J., Arganda-Carreras, I., Frise, E., Kaynig, V., Longair, M., Pietzsch, T., Preibisch, S., Rueden, C., Saalfeld, S., and Schmid, B. (2012). Fiji: an open-source platform for biological-image analysis. Nat Methods *9*, 676–682.

102. Folch, J., Lees, M., and Sloane Stanley, G.H. (1957). A simple method for the isolation and purification of total lipides from animal tissues. J. Biol. Chem. *226*, 497–509.

103. Giller, T., Buchwald, P., Blum-Kaelin, D., and Hunziker, W. (1992). Two novel human pancreatic lipase related proteins, hPLRP1 and hPLRP2. Differences in colipase dependence and in lipase activity. J. Biol. Chem. *267*, 16509–16516.

104. Loffredo, S., Borriello, F., Iannone, R., Ferrara, A.L., Galdiero, M.R., Gigantino, V., Esposito, P., Varricchi, G., Lambeau, G., Cassatella, M.A., et al. (2017). Group V Secreted Phospholipase A2 Induces the Release of Proangiogenic and Antiangiogenic Factors by Human Neutrophils. Front. Immunol. *8*, 443.

105. Kawabata, S., Miura, T., Morita, T., Kato, H., Fujikawa, K., Iwanaga, S., Takada, K., Kimura, T., and Sakakibara, S. (1988). Highly sensitive peptide-4-methylcoumaryl-7-amide substrates for blood-clotting proteases and trypsin. Eur. J. Biochem./FEBS *172*, 17–25.

106. Abraham, E., Naum, C., Bandi, V., Gervich, D., Lowry, S.F., Wunderink, R., Schein, R.M., Macias, W., Skerjanec, S., Dmitrienko, A., et al. (2003). Efficacy and safety of LY315920Na/S-5920, a selective inhibitor of 14-kDa group IIA secretory phospholipase A2, in patients with suspected sepsis and organ failure. Crit. Care Med. *31*, 718–728. https://doi.org/10.1097/01.Ccm.0000053648.42884.89.

STAR★METHODS

KEY RESOURCES TABLE

REAGENT or RESOURCE	SOURCE	IDENTIFIER
Antibodies		
ATGL	ThermoFisher Scientific	Cat#PA5-17436
Perilipin-1	Cell Signaling	Cat#D418
Adiponectin	R&D Systems	Cat#MAB10652
HSL	Cell signaling	Cat#4107S
GAPDH	Cell signaling	Cat#2118S
PLA_2	Proteintech	Cat#15843-1-AP
CLPS	St John’s	Cat#STJ28447
PNLIP	Sigma-Millipore	Cat#ABS547
Rabbit polyclonal	Abcam	Cat#ab208670
Horseradish peroxidase-conjugated	Millipore Corp	
Biological samples		
Mice ob/ob (B6. Lep ob/J)	LAB study	
cells	LAB study	
Human	Mayo Clinic	
Human	University of Pittsburgh	
Chemicals, peptides, and recombinant proteins		
Varespladib sodium (LY315920NA/S-5920)	Chemietek	
Orlistat	Cayman Chemical	
Complete	Roche	
1,2-dioleoyl-*sn*-glycerol-3-phosphocholine	Avanti lipids	
1,2-dioleoyl-*sn*-glycero-3-phospho-(1′-rac-glycerol) (sodium salt)	Avanti lipids	
Trilinoleate	FISHER HEALTHCARE	Cat#T1388
Triolein	FISHER HEALTHCARE	
BODIPY(R) FL dye-labeled acyl chain	ThermoFisher	
Isoflurane USP	Piramal Critical Care	
$CaCl_2$	Sigma-Aldrich	
Tris–HCl	Sigma-Aldrich	
imidazole	Sigma-Aldrich	
Triton X-100	Sigma-Aldrich	
Tween 20	Sigma-Aldrich	
SDS	Sigma-Aldrich	
Boc-Gln-Ala-Arg-MCA	Peptides International	
Honeybee venom PLA2	Sigma-Aldrich	
IL-12	PeproTech	Cat#:210-12
IL-18	R&D Systems	Cat#9139-IL-010
Caerulein	Bachem	Cat#H-3220.0001BA
PLA2	Production in Lab	
CLPS	Production in Lab	
PNLIPmcherry	Production in Lab	
$PNLIP^{S152G}$mcherry	Production in Lab	
PPLE	Sigma-Aldrich	

(*Continued on next page*)

Continued

REAGENT or RESOURCE	SOURCE	IDENTIFIER
Critical commercial assays		
Chem8	Abbott Vascular Inc	Cat#09P31-26
Lipase kit	Pointe scientific	Cat#3029590322
NEFA kit	Fujifilm	Cat#991-34891
Amylase assay	Pointe scientific	Cat# A7564-120
PLA2 assay	ThermoFisher	
LDH	Roche Applied Sciences	
CtnI kit	Abbott Vascular Inc	Cat#03P90-25
CK-MB	Abbott Vascular Inc	
Pico green	ThermoFisher	
Pierce ECL2	ThermoFisher Scientific	
MILLIPLEX MAP Magnetic Bead Panel assay	Millipore	Cat#32132
Deposited data		
Raw and analyzed data	This paper	
Experimental models: Cell lines		
HeLa Cells	ATCC	
3T3-L1	ATCC	
EXPi293T	ThermoFisher	
Experimental models: Organisms/strains		
Mice: Ob/ob-C57BL6	Jax Lab	
Mice: Lean C57BL6	Jax Lab	
Recombinant DNA		
PNLIPmcherry plasmid	Vector builder	VB180328-1041npk
CLPS plasmid	Vector builder	VB210120-1163efc)
PNLIPS^{152}G-mcherry plasmid	Vector builder	VB210209-1179yxs)
PLA2 plasmid	Vector builder	VB200813-1694tng
Software and algorithms		
Mouse OX	Starr Life Science	
GraphPad Prism 9	GraphPad Software	
ImageJ Fiji		
xPONENT software	Luminex software	
Zen	confocal microscope (LSM 800 ZEISS	
Vevo 3100	FUJIFILM Visual Sonics Inc	
Microsoft's Visual C++	CT scan software	

EXPERIMENTAL MODEL AND STUDY PARTICIPANT DETAILS

Human studies: All studies were approved by the institutional review board of the Mayo Clinic Foundation. Clinical residual samples were collected and processed as described previously.[32] The samples (October 2015 through January 2020) were residual necrotic material from the human pancreatic collections of patients with severe pancreatitis or diverticulitis based on electronic health record review. These are typically emergent samples of sick patients requiring surgery and are acquired unpredictably. Since there is no preliminary data, the sample size is based on emergent procedure sample availability. The samples are characterized or allocated by the diagnosis requiring the procedure. The material was immediately transported to the laboratory, aliquoted, and frozen at −80° for NEFAs, lipase, and phospholipase activities after a single freeze-thaw, boiled in Laemmli buffer for western blotting, or preserved in neutral buffered formalin and processed for histology.

Animal studies

All procedures were approved by the Institutional Animal Care and Use Committee (IACUC) of the Mayo Clinic Foundation. 10–12 weeks ob/ob (B6. Lep ob/J), male and female PNLIP KO[89] mice or C57bl6 and C57BL6 lean mice (Jackson Laboratories, USA) were used. All animals were housed with a 12-h light/dark cycle at 21°–25°C, fed standard laboratory chow, and allowed to

drink *ad libitum*. These were housed for at least 1 week to acclimate before experimentation. Three different groups were treated with doxorubicin, and the obese mice were treated with IL12/18mice, Caerulein, and PPLE. The mice's blood samples and fat were used for biochemical assays, and the live mice were used for echocardiography.

In vitro studies

Expi293 cells (Thermo Fisher Scientific, A14635) were used to produce recombinant Human PNLIPmcherry, Human PNLIPS152Gmcherry, and human CLPS. Cells were cultured in Expi293 expression medium and transfected with different plasmids. *Pichia pastoris* was used as a heterologous system for PLA2 expression. Yeast was cultured in BMGY media, and protein production was done in BMMY.[90] Cells were obtained unauthenticated from the manufacturer, and the mycoplasma was not studied. Further details are provided under Proteins in the method details section below.

Cell lines

3T3-L1 cells were obtained from the American Type Culture Collection (ATCC, CCL-2). Cells were differentiated and were maintained in adipocyte maintenance medium for 3–4 days until the desired LD formed. These cells were used to dissect the role of recombinant proteins and PPLE *in vitro*. HeLa cells (ATCC) were cultured in DMEM and were exposed to different proteins (50µg.mL-1). Pellets and supernatants were used for western blotting, LDH, and FFA measurements. Further details are provided under Cell Line Culture and use in the method details section below.

METHOD DETAILS

Nationwide inpatient sample (NIS) data source studies

For the retrospective part of the study, we utilized the Nationwide Inpatient Sample (NIS) database (NIS) for the years 2010–2014. NIS is part of databases developed for the Healthcare Cost and Utilization Project (HCUP) through a Federal-State-Industry partnership sponsored by the Agency for Healthcare Research and Quality (AHRQ), HCUP data inform decision-making at the national, state, and community levels.[91] It is the largest publicly available all-payer inpatient healthcare database in the United States, yielding national estimates of hospital inpatient stays. Data is collected from all the states participating in HCUP and represents more than 97% of the United States Population. The NIS includes clinical and non-clinical variables for each hospital stay, including up to 25 discharge diagnoses and 15 procedures using the International Classification of Diseases, Ninth Revision, and Clinical Modification/Procedure Coding System (ICD-9-CM/PCS). Since NIS is de-identified data that is publicly available, it is exempt from the Institutional Review Board review and approval.

Study population

Patients with congestive heart failure were identified using ICD9-CM diagnosis codes 428.0–428.9 from all listed discharge diagnoses. Patients younger than 18, missing information on age, gender, and inpatient mortality were excluded. Patients with acute pancreatitis (AP) were also identified using ICD9-CM code 577.0. We further divided the patient population into two groups, with and without acute pancreatitis (AP). Patients with a diagnosis of pancreatic cancer (ICD156.2, 157.0–157.9) and chronic pancreatitis (ICD157.1) were also excluded from the analysis.

Definitions Of variables

The Nationwide Readmissions Database pre-defined variables were used to identify each patient's age (in years) and gender (male or female). The Charlson comorbidity index (CCI) was used to assess the comorbidity burden since co-morbid conditions are known to influence hospitalization outcomes negatively. It has been a widely used index to measure the severity of comorbidity burden from administrative databases.[92] The CCI score was modified by excluding CHF-related conditions from the calculation since the entire study population included in the study was CHF. The higher score indicates a more substantial burden of co-morbidity. Clinical variables that can affect CHF such as diabetes mellitus (ICD9-CM 250–250.7), Hypertension (ICD9-CM 401–405.99, 437.2), Acute Myocardial infarction (AMI; ICD9-CM 410–410.9) were identified. We also identified potential etiologies of AP, such as gallstones, associated cholecystitis, cholangitis, alcohol abuse, and hyperlipidemia. The rest of the patients without obvious etiology were classified as idiopathic AP. Mortality was also identified using the ICD9-CM diagnosis code.

Statistical analyses

Descriptive statistics were used to describe the study population, with categorical variables reported as percentages and numbers and continuous variables reported as mean. The prevalence of AP was calculated and was also compared with other conditions, such as AMI, without excluding non-CHF patients from all the discharges recorded in the years 2010–2014. Bivariate group comparisons were made between hospitalized CHF patients with AP and those without AP using chi-square tests and t-tests for categorical and continuous variables, respectively. Multivariate logistic regression analysis was used to adjust for odds ratios for inpatient mortality.

CT imaging analysis

CT scans for fat involvement were acquired with multidetector CT scanners (4–16 detectors) with a slice thickness of 5 mm from patients who had abdominal CT scans during the disease. CT images were retrieved from the institutional Q reads (Windows application developed using Microsoft's Visual C++) and identified by a single reader who analyzed the images using in-house software on a Windows workstation.

Doxorubicin (Doxo) induced heart failure

10-12-week-old obese male C57BL/6 (Jackson Laboratories, USA) mice were used for this experiment. Three different groups were treated with doxorubicin: lean mice, ob/ob, and ob/ob PNLIPKO. There were 7 mice in each group. A single dose of 15 mg/kg body weight Doxo (Pfizer Labs, NY, USA) was injected intraperitoneally to induce heart failure based on previous studies.[93–97] Carotid Pulse distension was measured using a Mouse oximeter (Starr Life Science, Pittsburgh, PA) at baseline and every 24 h until seven days. Mice were monitored for seven days or euthanized when they were moribund, whichever came first. Serums were collected to measure the Creatine Kinase-myocardial band (CK-MB), troponin I and gonadal fat pads used for western blots and TLC.

Acute pancreatitis models

The caerulein and IL12, 18 models were as previously described.[79] The carotid artery pulse distention and survival curves shown are from the two previously published models. The troponin-I levels were measured on the stored sera collected at the time of necropsy. IL12, 18 model: Briefly, IL-12 (PeproTech, 150ng/30g) and IL-18 (R&D Systems, 750 ng/30g) were given intraperitoneally. Each was dissolved in saline and given at time 0 and 24 h. Caerulein model: This was done by giving hourly injections of caerulein (50 mg/kg, in 0.1mL saline) for 12 h on two consecutive days as previously.[79] Animals were followed for 3 days or until moribund, whichever came first, when they were euthanized using carbon dioxide before harvesting blood (by cardiac puncture) and tissues. PPLE injection: These were done as previously described.[32] After shaving the abdominal surface and cleaning with 70% ethanol, Sterile (0.2 micron) Porcine pancreatic lipase (PPLE, Sigma Aldrich, St. Louis, MO) was injected into the abdominal fat under direct vision. Five units of PPLE in 0.2mL saline were injected hourly five times into alternating (right and left) fat pads of ob/ob mice as previously. [32] Lactated Ringer's (LR) was given subcutaneously (0.8 mL) three times per day. Mice were followed for 48 h or till moribund, whichever came first, and euthanized as above. Troponin-I was measured on tail vein samples at baseline or before euthanasia.

Assays on *in vivo* models: Blood samples were collected during the necropsy. Visceral adipose tissue was harvested, and pancreatic lipases, amylase, and phospholipase activities were measured in the homogenate. Other viscera, including the pancreas, were collected at the same time. The number of samples included in the final analysis is based on the availability of samples and blood volume available after execution of prior serum assays and I-Stat measurements (Abbott Laboratories) for blood creatinine. The remaining methods are detailed in the supplementary section.

CK-MB and troponin I assay

Measurements of cardiac troponin I (CtnI, 03P90-25, Abbott Vascular Inc) using an I-Stat cartridge and CK-MB (Pointe scientific C7562-65) were done following the company instructions. Abbott Vascular Inc).

Echocardiography

Mice (Ob/ob, ob/ob PNLIPKO) were sedated with isoflurane 1–2%, shaved, and hair was removed from the ventral thorax using a depilatory cream. Heart function was evaluated by echocardiography using a high-frequency small animal ultrasound system (Vevo 3100, FUJIFILM Visual Sonics Inc., Toronto, ON, Canada). It had a 15–30 MHz center frequency linear transducer (MX250, FUJIFILM Visual Sonics Inc.). The transducer was positioned parallel to the short and long axis of the Left ventricle (LV). Images were acquired using the Vevo LAB analysis software (v3.0). M-mode views of the heart were used to outline the endocardial and epicardial borders of the LV. The LV chamber was defined by the endocardial border. The LV myocardium was the space between the endocardial and epicardial borders. Diastolic, stroke volume (μL), and cardiac output were measured at M-mode in both the short and long axis for at least 2 consecutive measurements before and after the Doxo, PPLE, and PPLE + orlistat injections.

Histology and immunohistochemical studies

The pancreas and visceral fat of mice were fixed with 10% neutral buffered formalin (Fisher Scientific), embedded in paraffin, and sectioned. Whole pancreas paraffin section slides stained by hematoxylin & eosin (H&E) were used to determine pancreas and peri-fat acinar necrosis (PFAN). [26,27] Myeloperoxidase (MPO), PNLIP, and perilipin-1 were immunostained in paraffin-embedded sections of human autopsy tissues as previously. [26,32] Briefly, after deparaffinization and antigen epitope retrieval, tissues were incubated with a primary rabbit polyclonal antibody against MPO (dilution 1:50; ab208670, Abcam, Cambridge, MA), perilipin-1 (Cell Signaling D418; 1:50) or PNLIP (1:200, ABS547, Millipore), followed by application of horseradish peroxidase-conjugated (dilution 1:1000; Millipore Corp) secondary antibody. Staining was completed with chromogen incubation with a 3–4 amino-9-ethyl carbazole substrate kit for peroxidase and hematoxylin QS nuclear counterstain (Vector Laboratories, Burlingame, CA).

Injury parameters

Regarding the histopathologic features related to heart failure, the H&E slides of the pancreas and fat pad of mice treated with doxorubicin were reviewed, and the pathologic assessment, including fat necrosis and edema, was scored. A score of 0 (none), 1 (mild), 2 (moderate), or 3 (severe) was given in each condition. All sections were analyzed, and the final score for each abnormality per case represented the mean of the three scores. Scoring of histologic findings in this way has been employed by many pathologists and used large animal models of heart failure in our research.[98]

Terminal deoxynucleotidyl transferase dUTP nick end labeling (TUNEL)

The staining was done on paraffin sections of the lungs and kidneys to identify apoptosis, as described previously.[99] Digital images of sections were captured with a digital microscope (Axio Imager. M2 or Axio Observer.Z1; Carl Zeiss).

Cell lines culture and use

3T3-L1 cells were obtained from the American Type Culture Collection (ATCC, CCL-2) and were cultured in Preadipocyte Medium (PM-1-L1, ZenBio, Inc., USA) until 70% confluent in 20mm glass bottom 35mm dish compatible for confocal imaging (Cellvis, CA, USA) at 37°C in a humidified atmosphere 95% air and 5% CO2. After that, cells were differentiated for three days in a differentiation medium (DM-2-L1, ZenBio, Inc., USA). Differentiated cells were maintained in adipocyte maintenance medium (AM-1-L1, ZenBio, Inc., USA) for 3–4 days until the desired LD formed. HeLa cells (ATCC) were cultured in DMEM at 37°C in a humidified atmosphere (95% air and 5% CO2) until 70% confluent in a 20mm glass-bottom 35mm dish compatible for confocal imaging (Cellvis, CA, USA). Cells were also cultured in 24-well plates in HEPES media pH 7.4, exposed to different proteins (50 μg mL^{-1}), monitored for 8 h, and harvested at 1000×g. Pellets and supernatants were used for western blotting, LDH, and FFA measurements. All experiments in cell lines were separately done 3–5 times.

Confocal microscopy

HeLa cells or adipocytes were prepared and stained as recommended. Stained adipocytes were pipetted onto a glass slide, and a coverslip was placed on top. Before starting live cell imaging, cells were washed 3 times with PBS, followed by staining for LDs with HCS LipidTOX Green neutral lipid stain (Ex/Em ~488/510, Thermo Fischer Scientific, USA) and TO-PRO-3 (Ex/Em~642/661, Thermo Fisher, USA). Live images were captured at 3-min intervals for the indicated periods using a 20x objective of a laser scanning confocal microscope (LSM 800 ZEISS) and combined as a time-lapse movie using Zen software. Cells were treated depending on the experiment by purified PNLIPmcherry (50 μg mL^{-1}), Colipase (5 μg), HbPLA2 (50 μg of 600–2400 units/mg stock, Sigma-Aldrich, USA), PNLIPRP2mcherry (50 μg.mL-1), or PPLE (0.5 mg.mL-1, Sigma Aldrich).

Calcium oscillation and contractions of cardiomyocytes

Using confocal microscopy (LSM 800), we studied the calcium oscillations and contractions in the cardiomyocytes loaded with Fluo-4 a.m. (F14201, Thermo Fisher). These parameters were measured after treatment of isolated cardiomyocytes with 150μM LA for 800s in HEPES media pH 7.4. Live images were captured at 10-s intervals using 63x Plan-Fluor oil immersion objective and combined as a time-lapse movie using Zen software. Measurement and quantification of cardiomyocyte or cardiac contractions were processed with MYOCYTER.[100] Images were analyzed using FIJI ImageJ.

Cell injury markers and LD loss

LDH release in the media, indicating cell death, was quantified using a colorimetric cytotoxicity assay. Briefly, absorbance at 490 nm and background absorbance at 620 nm were measured in the HEPES buffer, pH 7.4, according to the LDH assay kit (Roche Applied Sciences, Indianapolis, IN) after different treatments. Results were expressed as a percentage of total LDH leakage normalized to the control (cells lysed with 1% Triton X-100). TO-PRO-3 uptake and LD loss, as a measure of dead necrotic cells, and lipolysis of adipocyte fat were quantified using ImageJ Fiji[101] as previously described. [32] The dsDNA was measured from the mouse serum, the media from treated HeLa and adipocytes thawed on ice, and a 15 μL aliquot of different samples was incubated with PicoGreen 101 and mixed by gently shaking the plate, after which fluorescence intensity was read by a FlexStation at Ex/Em ~490/515.

Luminex assays

Resistin, IL-6, and S100A8 levels were assayed from mice plasma treated with IL12, 18 and PPLE. IL-6 and S100A8 were determined from cell supernatants treated by recombinant proteins. Assays were performed with a MILLIPLEX MAP Mouse Cytokine/Chemokine Magnetic Bead Panel assay (Millipore) according to the manufacturer's recommendations on a Luminex 200 System (Invitrogen, Carlsbad, CA) and analyzed using xPONENT software.

Western blot analysis

Visceral fats were homogenized in RIPA buffer supplemented by proteases inhibitors cocktail (Complete, EDTA Free; Roche, Mannheim, Germany), and lysates boiled in 1X Laemmli sample buffer with SDS and beta-mercaptoethanol, protein concentrations were measured with a Pierce protein assay kit (Thermo Fisher Scientific, Rockford, IL) and equal protein amounts were loaded and electrophoresed in a 4%–20% SDS-PAGE gel (Bio-Rad) and transferred to nitrocellulose membranes (Bio-Rad). Membranes were blocked

with a 5% blocking grade blocker (Sigma-Aldrich) with 0.5% Tween 20 (TBST) for 1 h. Supernatant and pellets from 3T3-L1 and HeLa cells exposed to recombinant enzymes (PNLIPmcherry, Colipase, $HbPLA_2$) or PPLE for 8 h, were lysed with RIPA buffer, and the protein concentration of the samples was determined by the by Pierce protein assay kit (Thermo Fisher Scientific, Rockford, IL). Equal amounts of protein were treated, as mentioned above. Western blot analysis was performed by incubation with primary antibodies detailed below: anti-adiponectin (1:1000, MAB10652, R&D Systems), anti-ATGL (1:1000; PA5-17436, Thermo Fisher Scientific), anti-PNLIP/PNLIPRP2 (1:10,000; a kind gift from Dr. Mark Lowe, University of Pittsburgh; this gives a 50kDa band for PNLIP and 52kDa bands for PNLIPRP2[66]), anti-Perilipin-1 (1:200, D418, Cell signaling technologies), anti-HSL (1:1000; 4107S, Cell signaling), anti-PLA_2 (1:1000, 15843-1-AP, Proteintech), CLPS (STJ28447,St John's) and appropriate horseradish peroxidase-labeled secondary antibodies at a concentration of 1:10,000 were used to detect the signal using ECL2 western blotting substrate (Thermo Fisher Scientific). Bands were visualized by chemiluminescence using electrogenerated chemiluminescence Pierce ECL Plus Western Blotting Substrate (Thermo Fisher Scientific).

Lipid extraction and thin-layer chromatography

Fat samples were homogenized in PBS (1:10 ratio). Homogenate was sonicated, and 50μL was taken for lipid extraction by the Folch method. [102] Similarly, lipids were extracted from cells (HeLa, 3T3-L1) treated by PPLE or recombinant enzymes. Pellets were sonicated, and 100μL taken for lipid extraction by the Folch method. Lipid extracts were evaporated under nitrogen and brought to a final volume of 100μL in chloroform. Woelm Silica gel G, 250-micron plates (Analtech, P16011) were prepared as previously described. [32] 20μL from different extractions were spotted on a line 1 inch from the bottom of a prewashed plate. The plate was placed in the saturated tank and ran until the solvent front reached 1 inch from the top. The plate was air dried (5 min), sprayed thoroughly with primuline solution from an all-glass atomizer, and allowed to air dry again before visualizing under UV light.

Materials

Varespladib sodium (LY315920NA/S-5920) was purchased from Chemietek (Indianapolis, USA, 99.99% purity). Orlistat was purchased from Cayman Chemical (Ann Arbor, MI). A protease inhibitor cocktail (Complete, EDTA free; Mannheim, Germany) was purchased from Roche. The soybean trypsin inhibitor was from Life Technologies Corp. Triton X-100, Tween 20, and imidazole was purchased from Sigma-Aldrich (St. Louis, MO). Specific reagents for cell culture, transfection, and viability assays are described under the specific methods.

Lipids

1,2-dioleoyl-*sn*-glycerol-3-phosphocholine (DOPC), 1,2-dioleoyl-*sn*-glycero-3-phospho-(1′-rac-glycerol) (sodium salt) (DOPG) were purchased from Avanti Lipids (Alabaster, USA). Glyceryl Trilinoleate (GTL), triolein (GTO), Triton X-100, and Tween 20 were purchased from Sigma-Aldrich (St. Louis, MO). Just before use, triglycerides were sonicated into the media in a two-step manner to ensure the lipids stayed in the solution.

Proteins

Recombinant $HbPLA_2$ and PPLE were purchased from Sigma-Aldrich (St. Louis, MO). Recombinant Human PNLIPmcherry, Human $PNLIP^{S152G}$mcherry (inactive PNLIP), and human CLPS were produced in Expi293 cells (Thermo-Fisher Scientific, A14635). Briefly, cells were cultured in Expi293 expression medium in a humidified atmosphere at 37°C and 8% CO_2 for 24h. 3×10^6 cells were transfected by Human PNLIPmcherry plasmid (VB180328-1041npk), Human $PNLIPS^{152}$G-mcherry (VB210209-1179yxs), and Human CLPS (VB210120-1163efc), respectively for 20h. Proteins were induced according to the Thermo-Fisher protocol (Thermo-Fisher Scientific, A14635). Collected media were pooled and purified using HisPur Cobalt Resin according to the manufacturer's protocol (Thermo-Fisher Scientific, 89964). Elution of tagged proteins was done with imidazole, and the pure proteins were gathered and concentrated in DPBS using Amicon Ultra-15 Centrifugal Filter Units (Millipore, C7715). The *Pichia pastoris* expressing human PLA_2 (VB200813-1694tng) was cultured for 72 h, and the PLA_2 secretion was induced using 2% methanol every 24h. The media was collected, the secreted PLA2s were loaded on cobalt affinity, and SDS-PAGE was used to check the protein quality.

Enzymatic activities

Serum lipase and amylase activities were measured according to the manufacturer's protocol (Pointe Scientific, Canton, MI). The lipase assay is described in detail elsewhere [103] measures pancreatic lipase activity, which is colipase and bile salt-dependent.

PLA_2 activity assays from mice fat pads were conducted using the EnzCheckPhospholipase A_2 Assay Kit (Life Technologies Corporation, California, USA). The assay kits are a simple, fluorometric method designed to continuously monitor PLA_2 activity using a Flex Station 3 microplate reader (Molecular Devices, Sunnyvale, CA, USA) according to the manufacturer's protocol. The substrates are specific for each enzyme (400 μg mL^{-1}) and are dye-labeled glycerophosphoethanolamine and glycerophosphocholine with a BODIPY(R) FL dye-labeled acyl chain at the *sn-1* or the *sn-2* position. The results are a PLA_2-dependent increase in BODIPY(R) FL fluorescence emission detected at approximately 515 nm.

A modified assay was used to measure the PLA_2 activity of PPLE and $HbPLA_2$[72,104] using 10 mM DOPC or DOPG as substrates. Briefly, a PLA_2 substrate consisting of 10 mM DOPC was prepared in 20 mM NaCl, 2 mM $CaCl_2$, 10 mM Tris–HCl, and pH 8.0. The enzyme (1 μg mL^{-1}) was added to 96-well plates, and PLA_2 activity was measured by NEFA kit.

Trypsin activity was measured fluorometrically using Boc-Gln-Ala-Arg-MCA (Peptides International, Louisville, KY) as the substrate according to the method of Kawabata et al.. [105] The PPLE homogenate in PBS, pH 7.4, was centrifuged at 10,000g for 10 min. The supernatant was taken and assayed. Briefly, the supernatant was added to a black 96-well microplate with a clear bottom (Corning, NY). Trypsin substrate was added to an assay buffer containing 50 mM Tris-HCl, 150 mM NaCl, 1 mM CaCl2, and 0.1 mg/mL bovine serum albumin. The mixture (195 μL) was added into the microplate, and the fluorescence emitted at 440 nm after excitation at 380 nm was monitored. The enzyme activity was calculated as an increasing amount of fluorescent product formation per minute (ΔFlu/min = Fluorescence at that time – Previous minute Fluorescence).

Pharmacological inhibition

Varespladib sodium (var-Na) is a potent, low molecular weight, specific inhibitor of $sPLA_2$.[106] The var-Na was dissolved in water for the biological assays. To investigate whether var-Na was capable of inhibiting PLA_2 activity, PPLE was incubated with 50 μM of the var-Na for 15 min, and the PLA_2 activity was measured using 10 mM DOPC as substrate in Tris-HCl, pH 8, 1 mM $CaCl_2$, and 10 mM NaCl at 37°C. Similarly, orlistat (50μM) was used to determine its lipase inhibitory effect on PPLE using GTL as a substrate in PBS buffer at 37°C. The var-Na or orlistat was added to each well of 3T3-L1 cells treated by PPLE, and the NEFA release was measured using the NEFA kit.

QUANTIFICATION AND STATISTICAL ANALYSIS

Statistical analyses were performed using GraphPad Prism 9. Independent variables for *in vivo* and *in vitro* studies are shown as bar graphs reported as mean ± SD. Each point is shown. Line graphs were used for continuous variables. Significance levels were evaluated at $p < 0.05$. Data for multiple groups were compared to ANOVA versus controls, and values significantly different from controls are indicated with asterisks. The number of mice and sequences for each experiment are shown in the figures and figure legends.

Cell Reports Medicine, Volume *6*

Supplemental information

Visceral fat lipolysis by pancreatic lipases worsens heart failure

Nabil Smichi, Biswajit Khatua, Sergiy Kostenko, Cristiane de Oliveira, Bara El Kurdi, Kalpit Himmatbhai Devani, Shubham Trivedi, Megan Summers, Bryce McFayden, Sarah Navina, Krutika Patel, Sarah Jahangir, Marek Belohlavek, and Vijay P. Singh

Supplementary figures

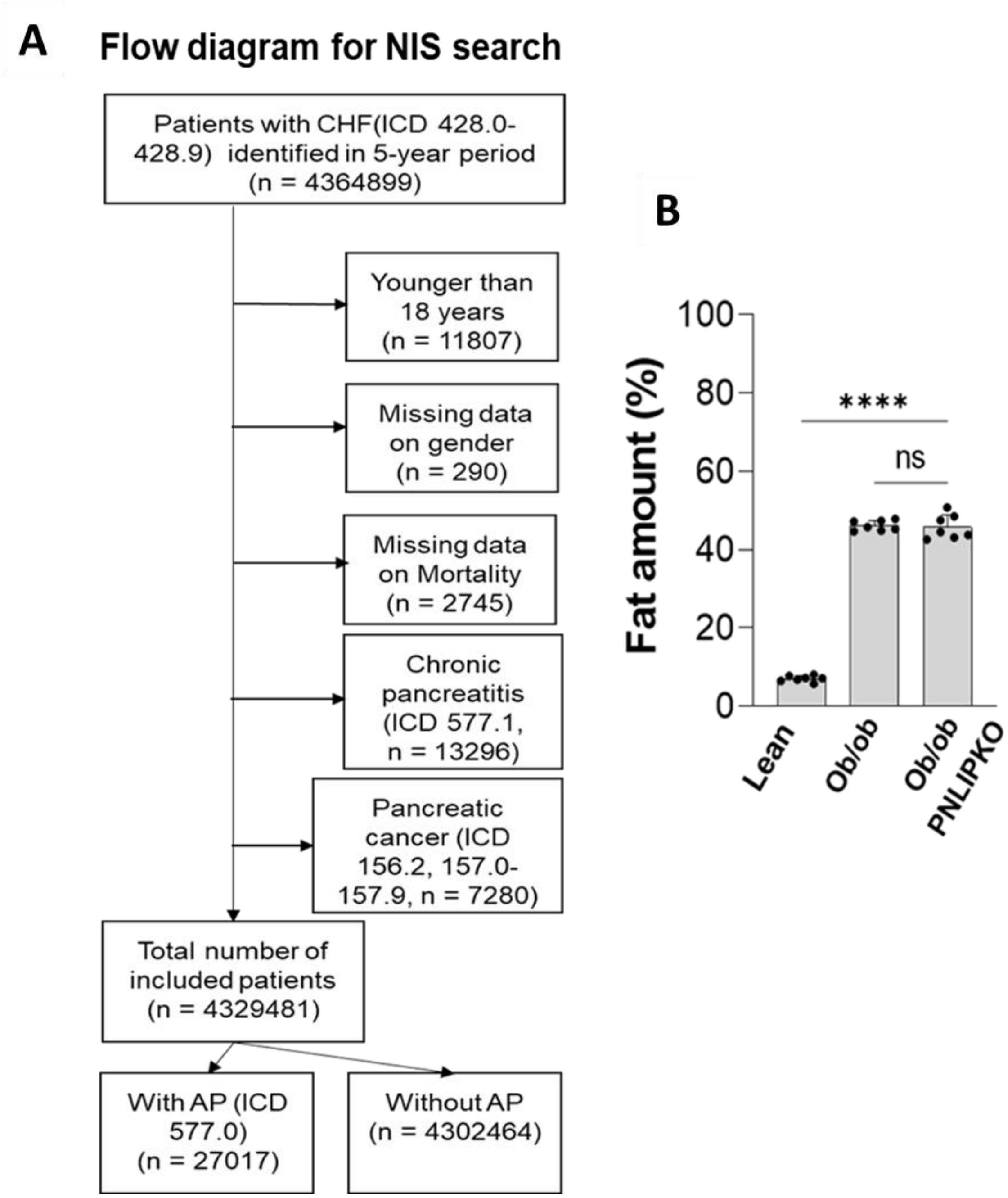

Figure S1: (A) The flow diagram for NIS search. **(B**) NMR data comparing the fat percentage in lean and ob/ob mice. Related to Figure 1.

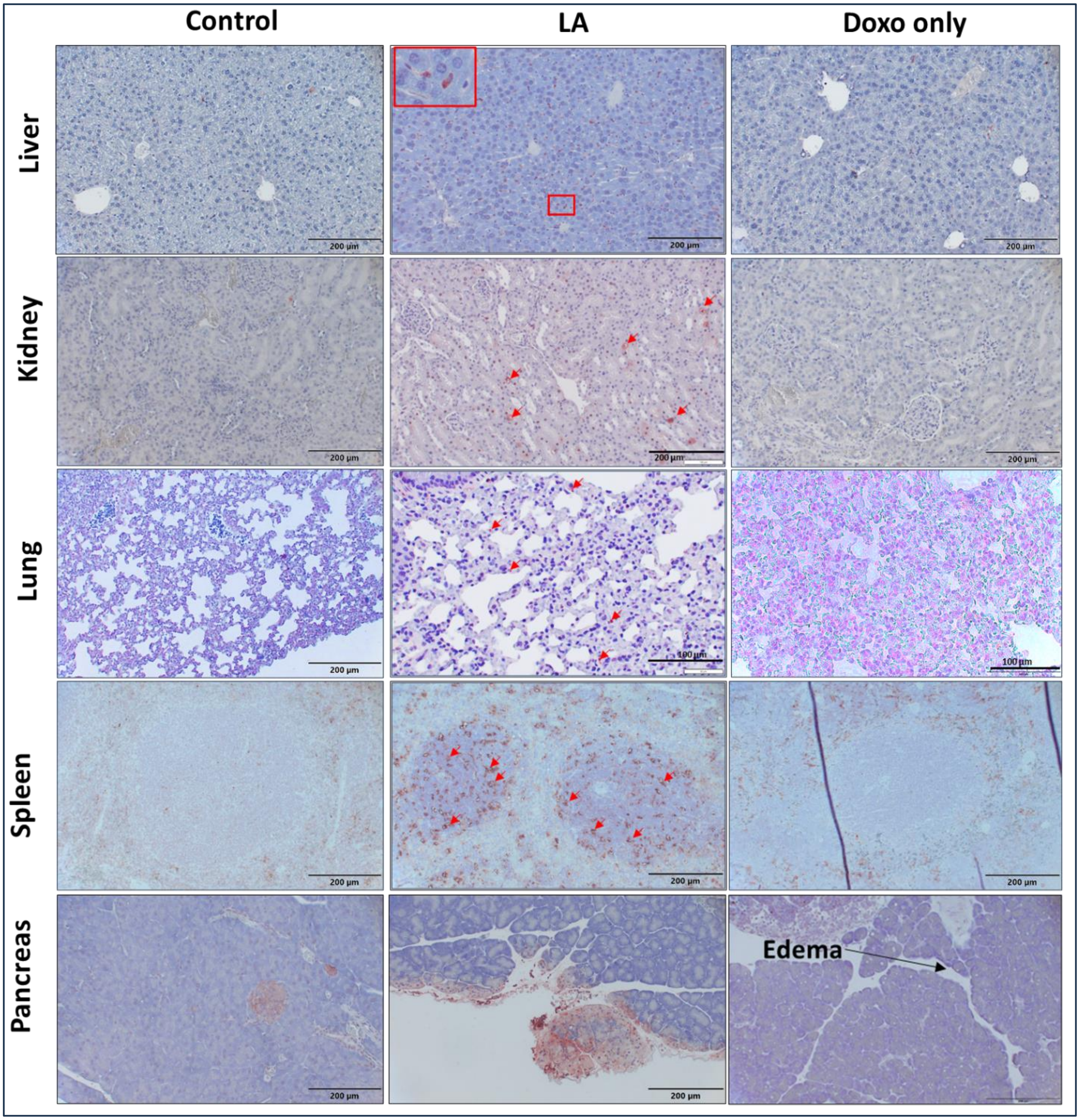

Figure S2: TUNEL stained images of the liver, kidney, lungs, spleen, and pancreas from different groups of mice. Related to Figure 1. The tissues represented in the image are mentioned on the left side, and the treatment group is above each column of images. The brown staining, emphasized with red arrows, shows the TUNEL positivity. Magnification was 20x.

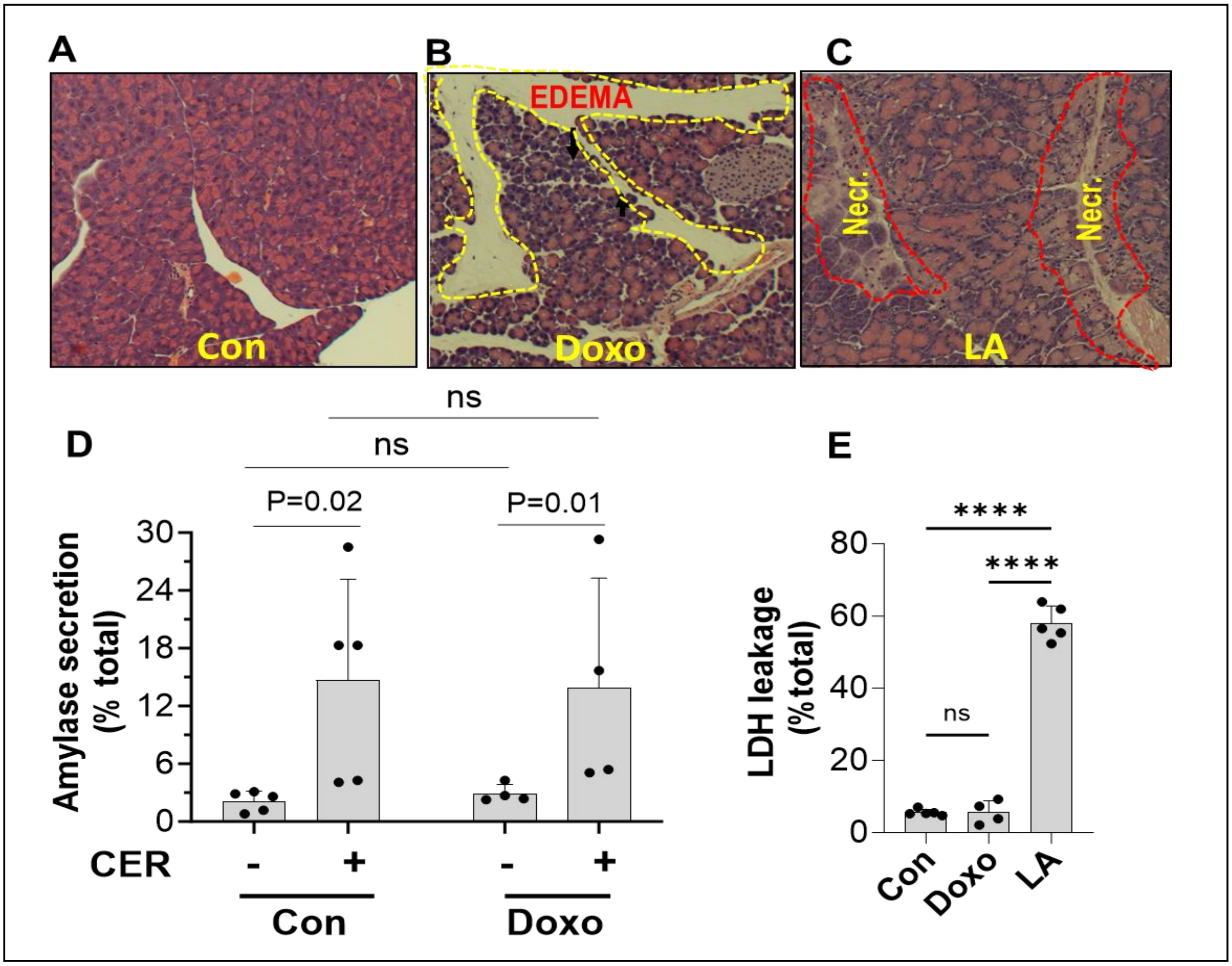

Figure S3: Effect of doxorubicin on pancreatic injury *in-vivo* and acinar function and injury *ex-vivo*. Related to Figure 1. Pancreas H&E staining images (10X) of untreated (**A**), Doxo (15mg/kg BW, B), and LA (0.2% BW, C) treated mice. LA-induced acinar necrosis [Necr.; Red dashed outline in (**C**)]but Doxo causes interstitial edema [yellow dashed outline; (**B**)] consistent with heart failure. (**D**) Physiological (10^{-10}M) caerulein (CER)-induced amylase secretion measured as % total is similar in Doxo ($1mg.mL^{-1}$) treated and untreated acini. (**E)** Doxo ($1mg.mL^{-1}$) treated acini showed similar LDH leakage compared to untreated acini over 4hrs, while LA (100mM) induced 58±5%leakage.**p<0.01, ***p<0.001; ****p<0.0001 indicate a significant difference between these groups compared to the control as determined by 1-way ANOVA. Data are represented as mean/± SD.

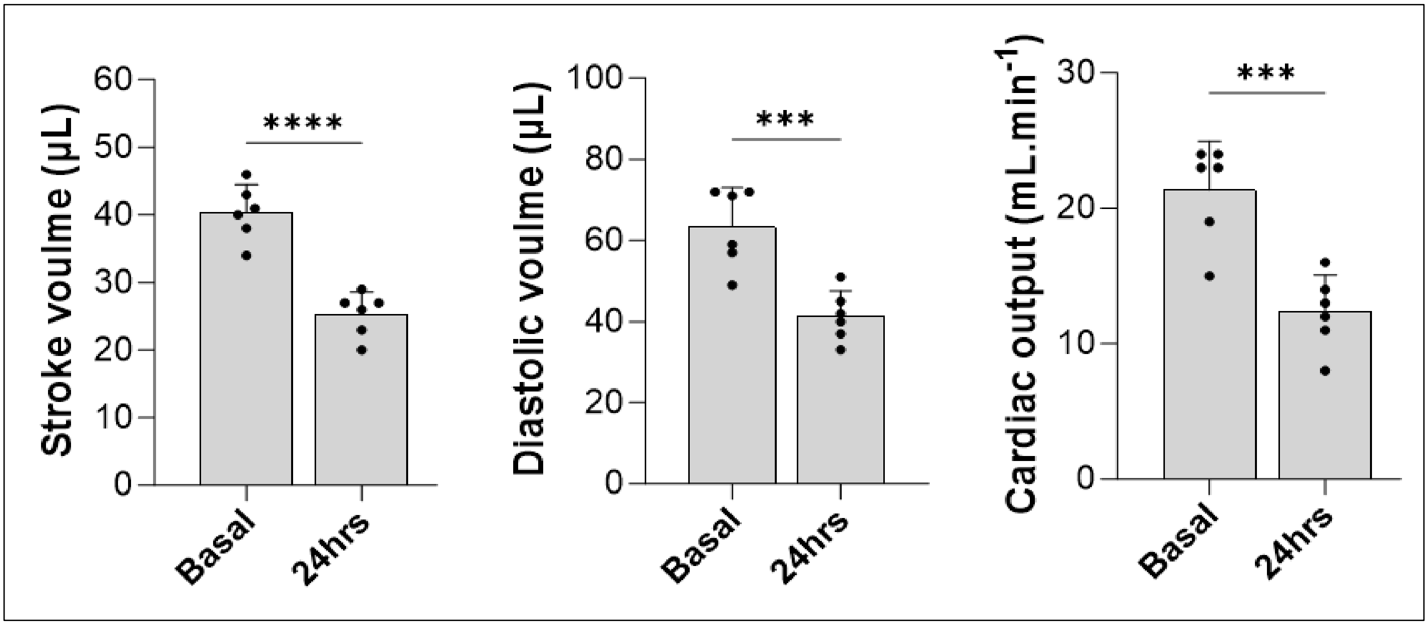

Figure S4: Echocardiographic parameters in mice with Doxorubicin (Doxo) induced heart failure measured at baseline and 24 hours after Doxo. Related to Figure 1. From left to right, left ventricular stroke volume (µL), end-diastolic volume (µL), and cardiac output (mL.min^{-1}). The ***p<0.001 and ****p<0.0001 indicate a significant reduction at 24 hours compared to the baseline. Data are represented as mean/± SD. The parameters were collected in the short-axis mode.

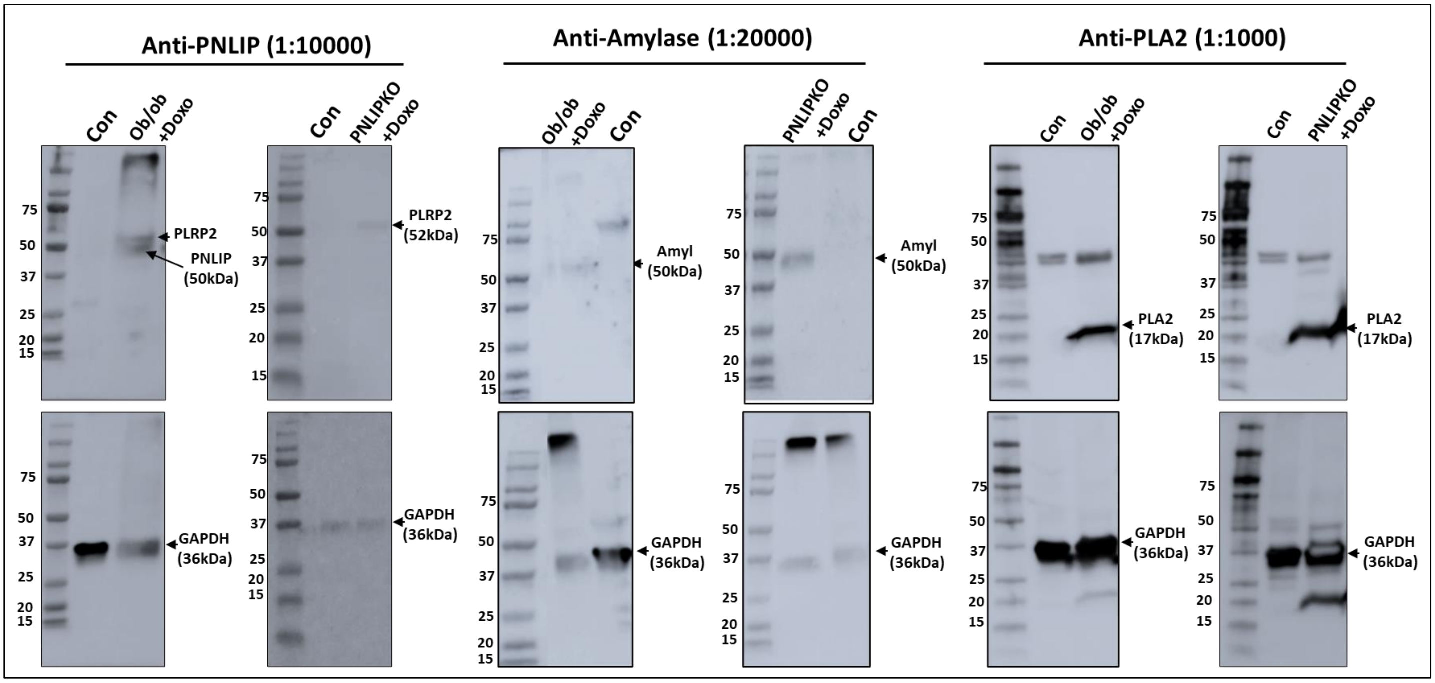

Figure S5: Western blot full gel images showing the leakage of PNLIP (50kDa), PNLIPRP2 (52kDa), PLA_2 (17kDa), and amylase (50kDa) from the pancreas into the fat pad of ob/ob and ob/ob PNLIP KO mice treated by doxorubicin (Doxo). The GAPDH (36kDa) was used as a control. Related to Figure 1.

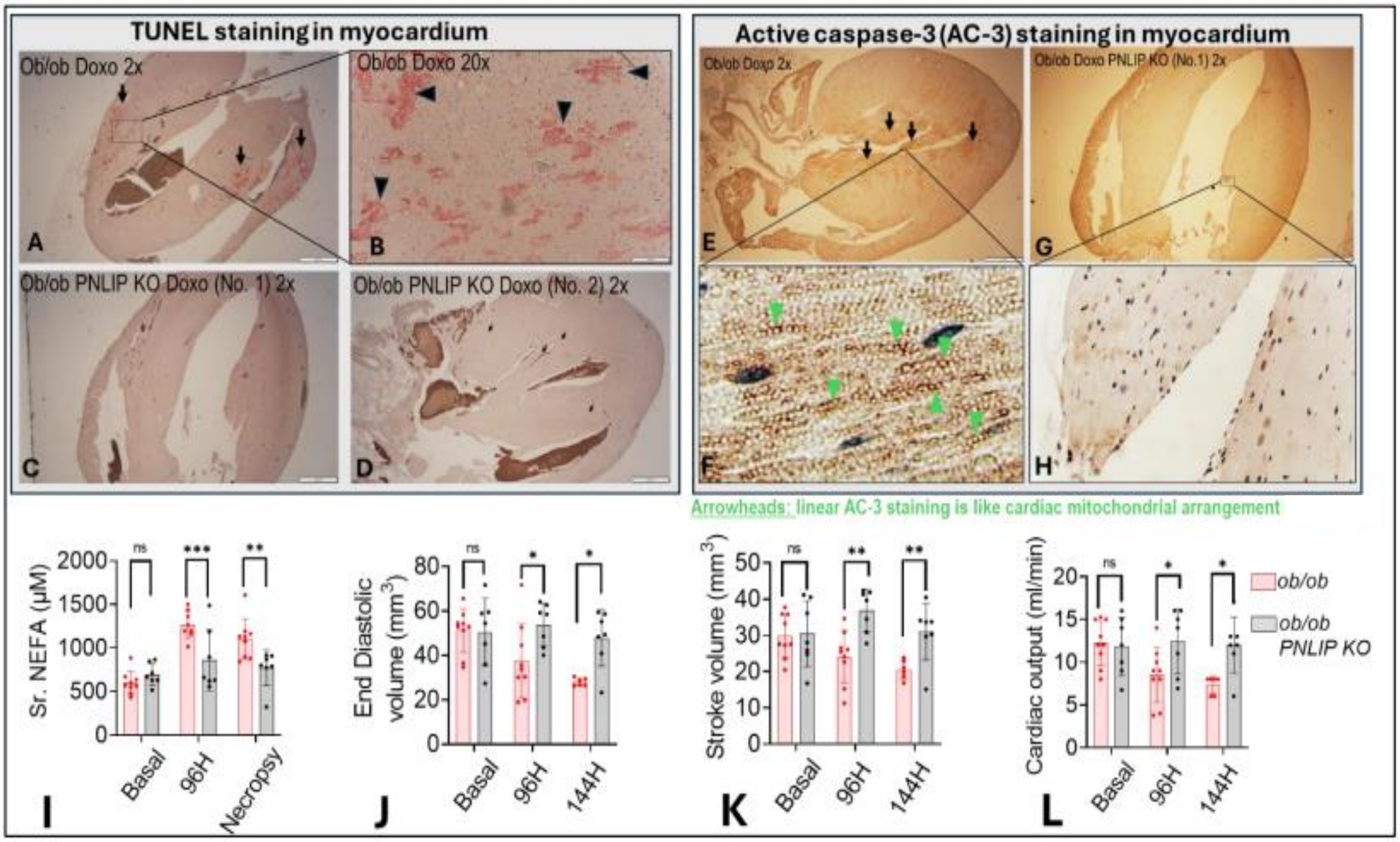

Figure S6: Effects of genetic deletion of PNLIP (PNLIP KO) on doxorubicin (Doxo) induced Cardiac injury parameters and serum NEFA in genetically obese ob/ob mice. Related to Figure 1. (**A-H)** Histologic images of the heart stained for TUNEL (**A-D**) or active caspase-3 (**E-H**). The treatment, genetic background and magnification are mentioned on the top. **B, F**, and **H** are zooms of the corresponding insets shown. Black arrowheads in **B** point to strong TUNEL staining in the heart of the ob/ob mouse treated with doxorubicin. Green arrows in **F** point to magnified views showing a strong linear round pattern of active caspase-3 (AC-3) staining in the heart of an ob/ob mouse treated with doxorubicin. This strong linear pattern resembles mitochondrial arrangement in mouse heart muscle, shown in the transmission electron microscopy (TEM) image below. These sections are being processed for TEM. (**I-L)** Bar graphs show a comparison of parameters for ob/ob mice (red) vs. ob/ob PNLIP KO mice (gray) at baseline (basal), day 4 (96 hrs), day 6 (144hrs) or necropsy. These are NEFA (**I**) and Echocardiography parameters: end-diastolic volume (**J**), stroke volume (**K**), and cardiac output (**L**) measured in the long-axis mode.*p<0.05, **p<0.01, ***p<0.001; indicate a significant difference between these groups compared to the control as determined by 1-way ANOVA. Data are represented as mean/± SD.

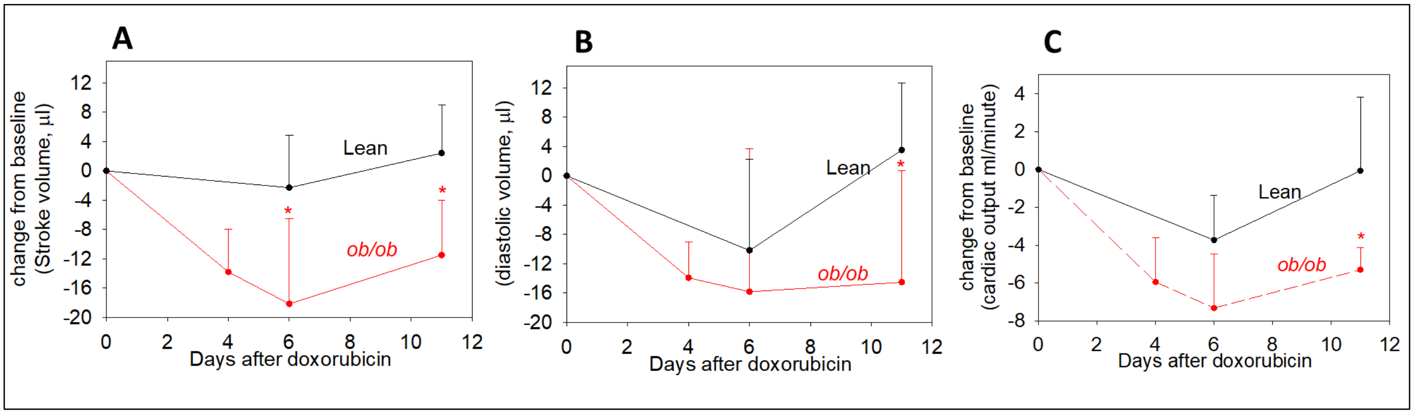

Figure S7: Effect of low-dose doxorubicin (2.5mg/kg/day IP) model on change in cardiac parameters (mentioned on Y-axis) in lean (C57bl/6, black color) and obese mice (ob/ob, red color). Related to Figure 1. Data are represented as mean/± SD.

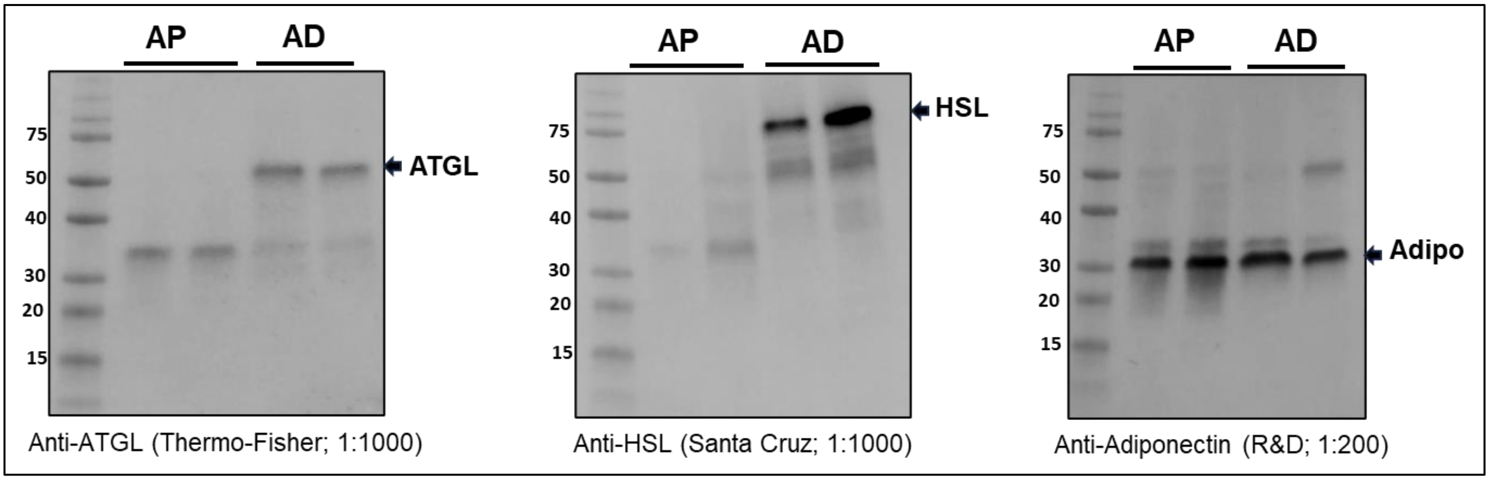

Figure S8: Western blot full gel images comparing detectable ATGL, HSL, and adiponectin (Adipo) bands in fat from human pancreatitis (AP) and diverticulitis (AD) samples. Related to Figure 2.

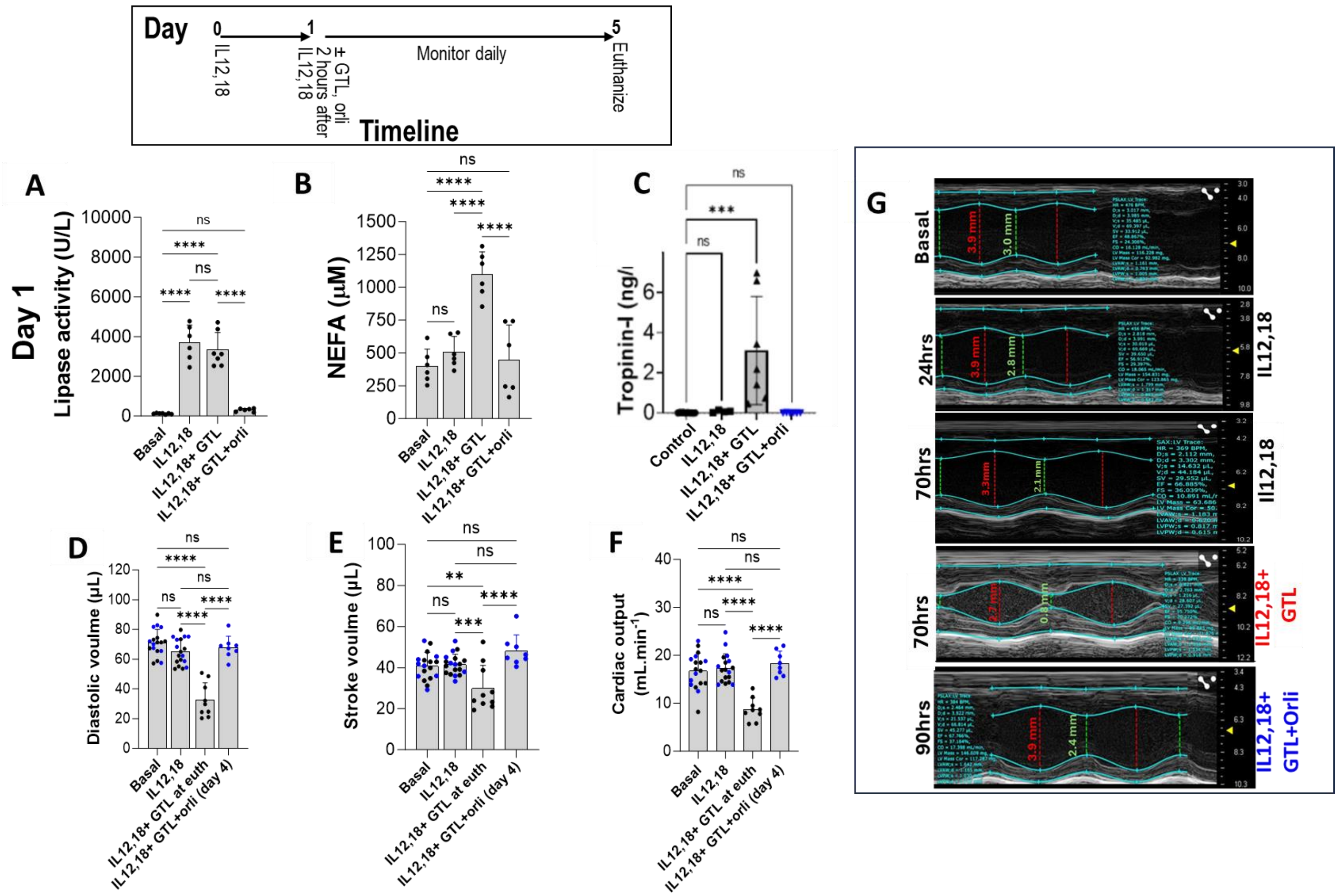

Figure S9: **Timeline and parameters of IL12,18 pancreatitis in lean mice.** Related to Figure 3. (**A**) Serum lipase and NEFA (**B**) in control mice (basal) compared to IL12,18, IL12,18 +GTL, and IL12,18+orlistat (Orli) ones, and IL12,18 AP. (**C**) The troponin-I levels in the tail vein prior to euthanasia of these mice. **D-F:** Show echocardiography parameters at baseline or prior to euthanasia. These are end-diastolic volume (**D**), stroke volume (**E**), and cardiac output (**F**) measured in the long-axis mode. (**G)** shows representative M-mode Echocardiographic images at baseline (top panels), and after inducing IL12, 18 pancreatitis (other panels) at time points mentioned on the left side of the corresponding treatment panel. **p<0.01, ***p<0.001; ****p<0.0001 indicate a significant difference between these groups compared to the control as determined by 1-way ANOVA. Data are represented as mean/± SD.

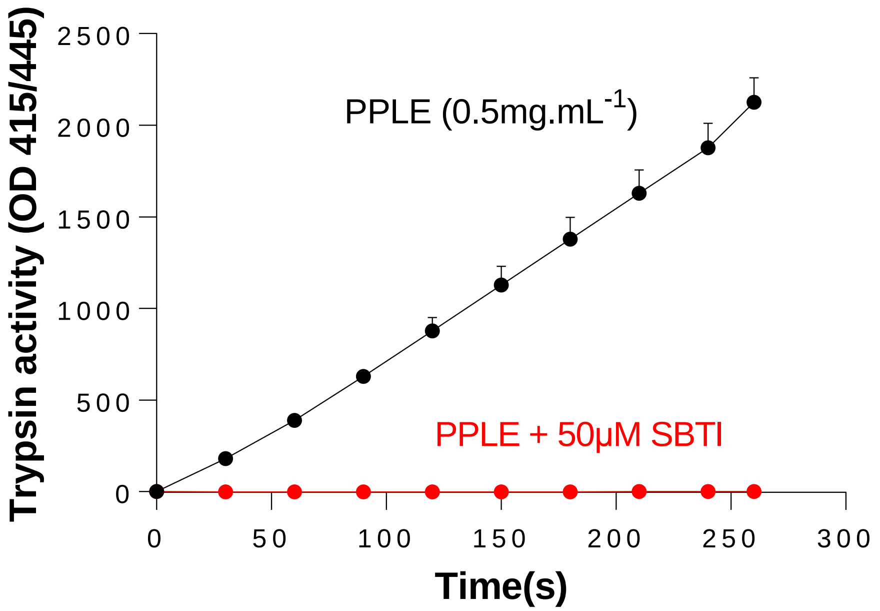

Figure S10: Trypsin activity in the PPLE Extract and its inhibition by soybean trypsin inhibitor prior to injection into fat pads. Related to Figure 3. The trypsin activity of 0.5 mg. mL^{-1} was measured at 37°C in the presence of 50 µM of the soybean trypsin inhibitor (Trypsin inhibitor). **p<0.01. indicate a significant difference between different groups using the T-test. Data are represented as mean/± SD.

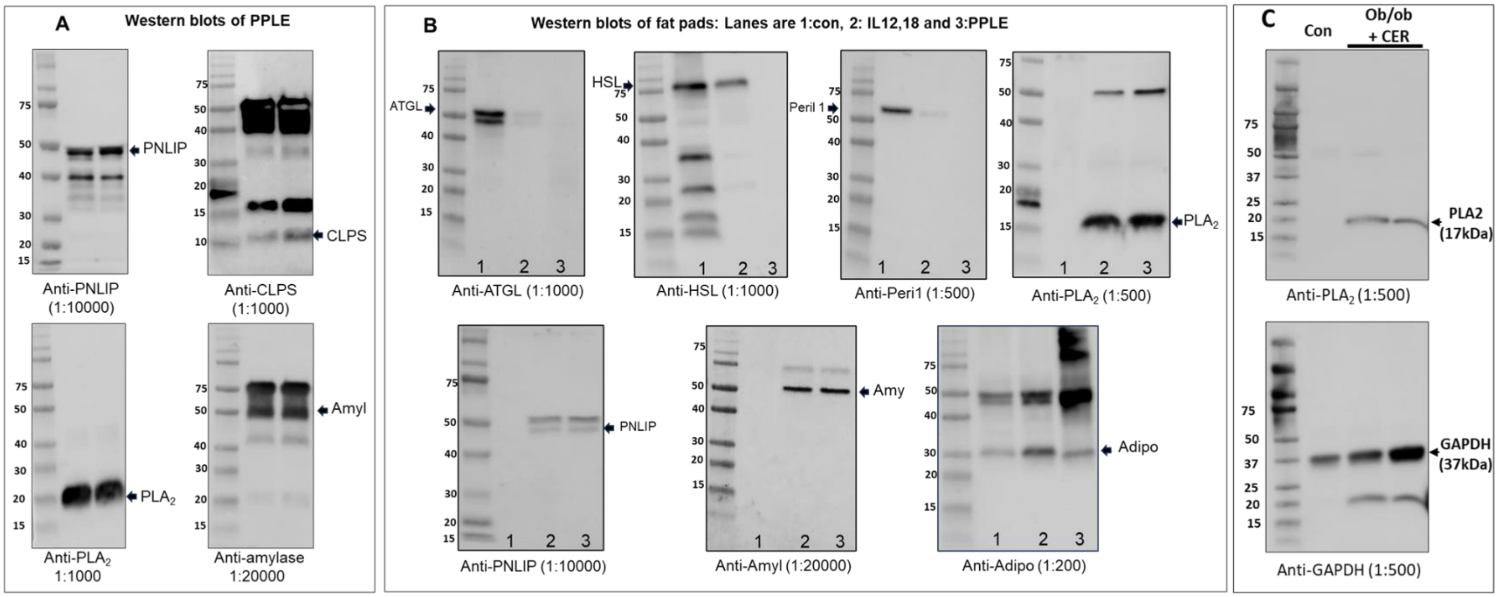

Figure S11: Western blot full gel images showing detectable pancreatic enzymes in PPLE, fat pads of mice. Related to Figure 3. **(A)** PNLIP, CLPS, PLA_2, and amylase in porcine pancreatic extract (PPLE). (**B**) Western blots for ATGL, HSL, perilipin 1 (Peri1), PNLIP, amylase (Amyl), and adiponectin (Adipo) bands in the fat pad of mice from control (Lane 1), treated with IL12;18 (Lane 2), and PPLE (Lane 3) models. (**C**) Western blot full gel image showing the leakage of PLA_2 into the fat pad of ob/ob treated with caerulein, the GAPDH was used as a control.

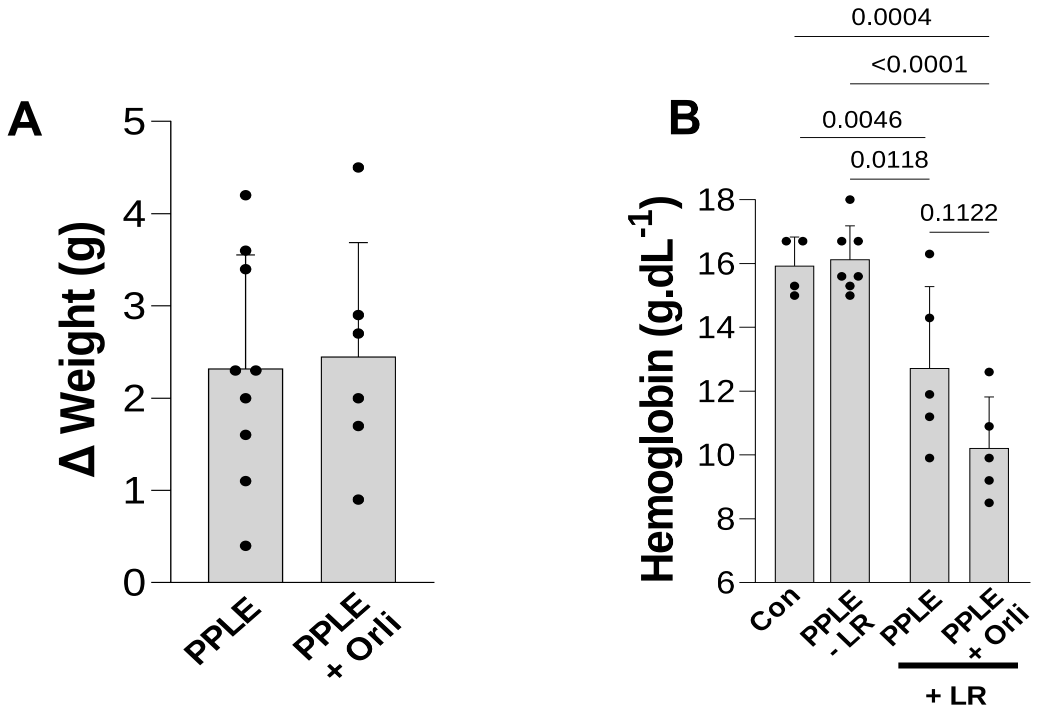

Figure S12: Comparison of the difference in weights from baseline (A) and blood Hemoglobin at the time of necropsy (B) of the PPLE group vs. PPLE + orlistat group receiving Lactated Ringer's (LR) fluid supplementation. Related to Figure 3. p-values show the significance between different groups. Note that both groups had a similar increase in weight and reduction in hemoglobin consistent when receiving LR, while the PPLE-LR group (B) had no reduction in hemoglobin. Thus, LR increased blood volume and caused hemodilution in both groups. Data are represented as mean/± SD.

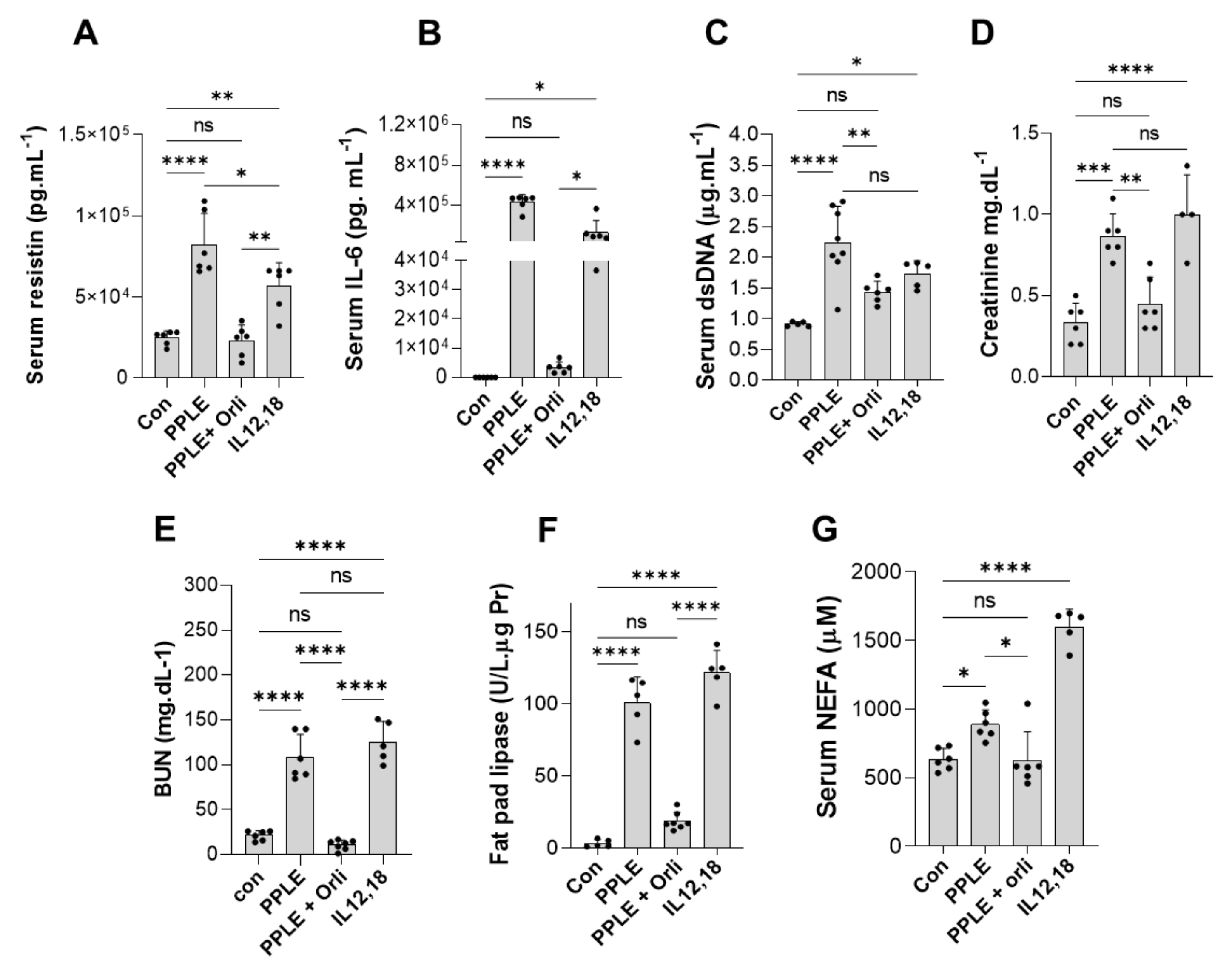

Figure S13: (A-E) Serum biochemical parameters, cytokines, and organ failure parameters in mice given IL12/18 or PPLE (+/-orlistat). Related to Figure 3. Serum Resistin (**A**), IL-6 (**B**), Serum dsDNA (**C**), creatinine (**D**), and BUN (**E**) in controls (con), and other mice treated by IL12/18 and PPLE (+/-orlistat). Pancreatic lipase activity (**F**) in fat pads and serum NEFA (**G**) of these mice at the time of necropsy. *p<0.05, **p<0.01, ***p<0.001; **** p<0.0001 indicate a significant difference between different groups using 1-way-ANOVA. Data are represented as mean/± SD.

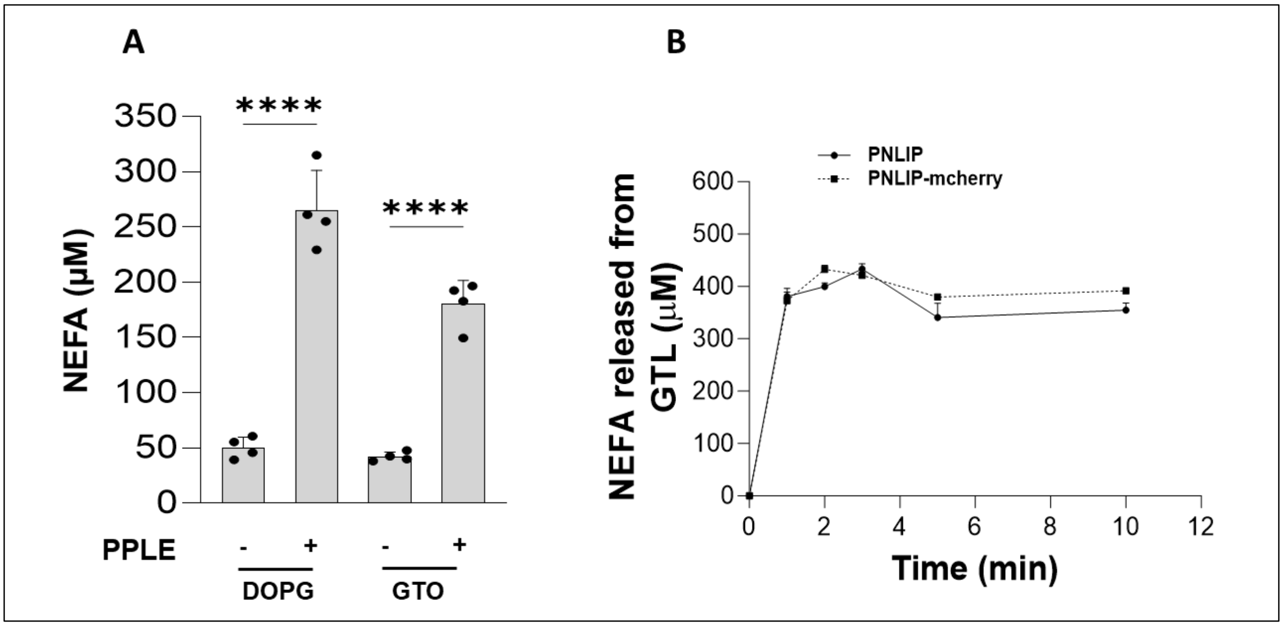

Figure S14: NEFA release from lipids after different treatments. Related to Figures 4 and 5. **(A)** NEFA release from 10 mM DOPG and 600µM GTO hydrolyzed by PPLE (0.5 mg.mL^{-1}). (**B**) Activity of PNLIP and PNLIPmcherry on GTL(600uM) as substrate. ****P<0.0001, indicates a significant difference between different treatments and significance determined by 1- way ANOVA. Data are represented as mean/± SD.

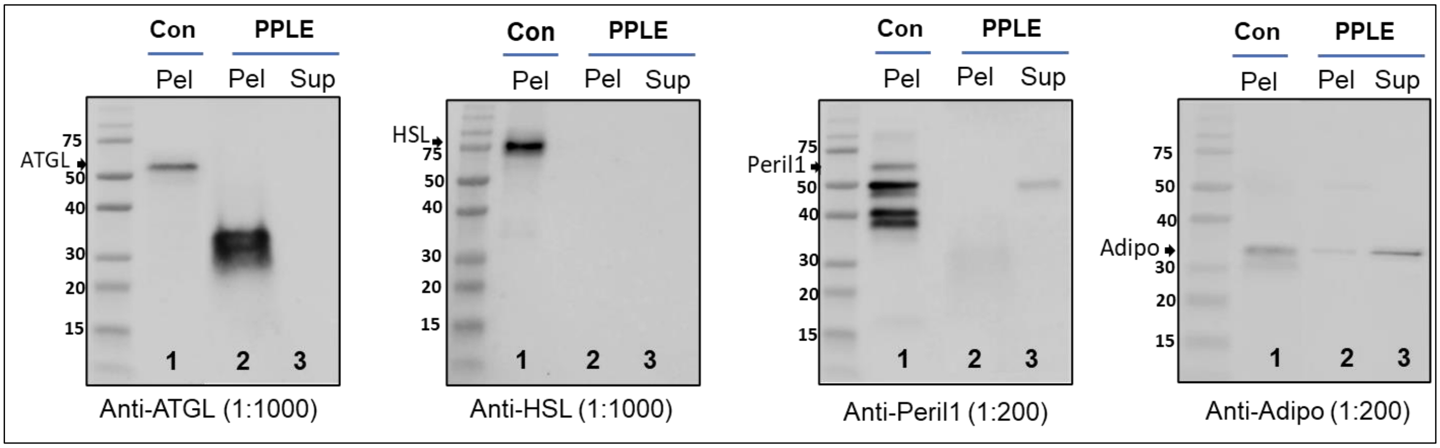

Figure S15: Western blot (Full gel) comparing adipocyte proteins in different fractions: Related to Figure 6. From left to right, these show ATGL, HSL, perilipin-1 (peri1), and adiponectin (Adipo) bands in 3T3-L1 pellet **(Pel)** and supernatant **(Sup).** Lane 1: cell lysate pellet, **Lane 2**: pellet of 3T3-L1 treated by PPLE, **Lane 3**: supernatant of 3T3-L1 treated by PPLE.

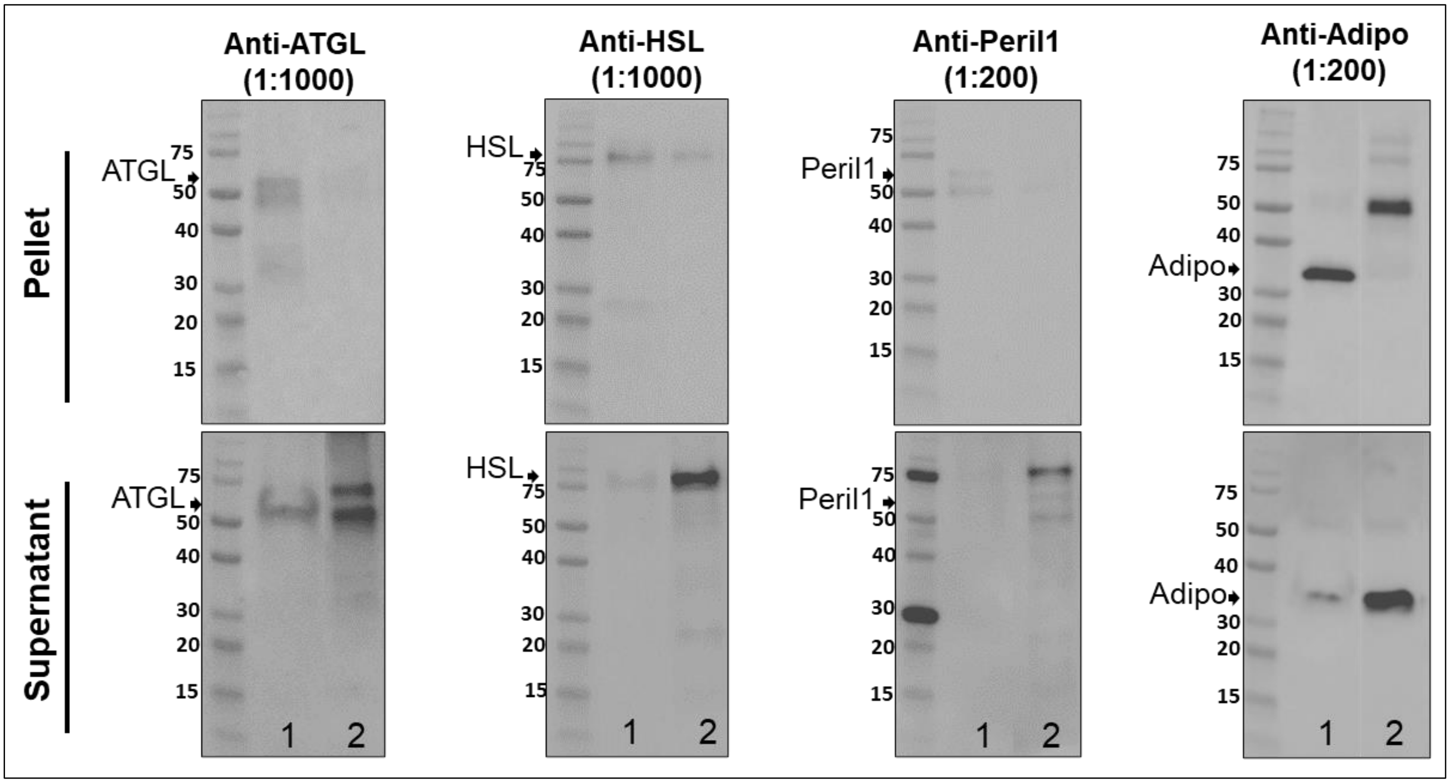

Figure S16: Western blot full gel images comparing detectable ATGL, HSL, Perilipin 1 (Peri-1), and adiponectin (Adipo) bands in the 3T3-L1 pellet and supernatant from 3T3-L1 control (**Lane 1**) and cells exposed to HbPLA$_2$ (**Lane 2**). Related to Figure 7. The arrows (➧) indicate the corresponding proteins.

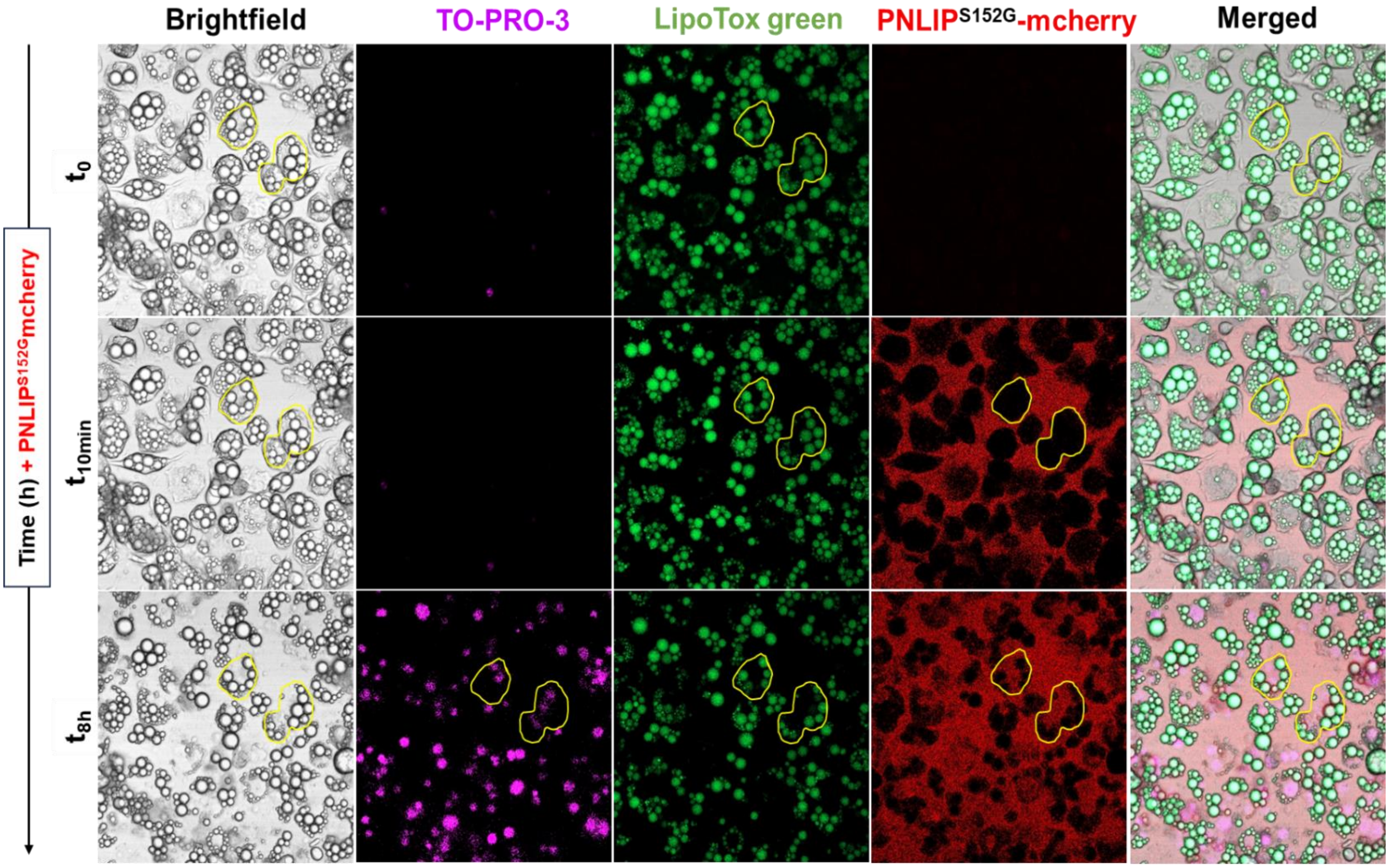

Figure S17: Live imaging representative of 5 different experiments showing the cell death of 3T3-L1 cells by HbPLA$_2$ and the absence of lipolysis in the presence of the PNLIP inactive form (PNLIP^{S152G}mcherry). Related to Figure 7. Cells were treated by these enzymes for 8h in HEPES media (pH 7.4) at 37°C.
